# Proteomic analysis of chicken embryonic trachea and kidney tissues after infection *in ovo *by avian infectious bronchitis coronavirus

**DOI:** 10.1186/1477-5956-9-11

**Published:** 2011-03-08

**Authors:** Zhongzan Cao, Zongxi Han, Yuhao Shao, Heyuan Geng, Xiangang Kong, Shengwang Liu

**Affiliations:** 1 State Key Laboratory of Veterinary Biotechnology, Harbin Veterinary Research Institute, the Chinese Academy of Agricultural Sciences, Harbin 150001, China

## Abstract

**Background:**

Avian infectious bronchitis (IB) is one of the most serious diseases of economic importance in chickens; it is caused by the avian infectious coronavirus (IBV). Information remains limited about the comparative protein expression profiles of chicken embryonic tissues in response to IBV infection *in ovo*. In this study, we analyzed the changes of protein expression in trachea and kidney tissues from chicken embryos, following IBV infection *in ovo*, using two-dimensional gel electrophoresis (2-DE) coupled with matrix-assisted laser desorption/ionization time-of-flight tandem mass spectrometry (MALDI-TOF-TOF MS).

**Results:**

17 differentially expressed proteins from tracheal tissues and 19 differentially expressed proteins from kidney tissues were identified. These proteins mostly related to the cytoskeleton, binding of calcium ions, the stress response, anti-oxidative, and macromolecular metabolism. Some of these altered proteins were confirmed further at the mRNA level using real-time RT-PCR. Moreover, western blotting analysis further confirmed the changes of annexin A5 and HSPB1 during IBV infection.

**Conclusions:**

To the best of our knowledge, we have performed the first analysis of the proteomic changes in chicken embryonic trachea and kidney tissues during IBV infection *in ovo*. The data obtained should facilitate a better understanding of the pathogenesis of IBV infection.

## Background

Avian infectious bronchitis (IB) is one of the most serious diseases of chickens. It is of economic importance in the poultry industry worldwide and is associated with respiratory disease, reduction in weight gain, poor egg production and quality, and decreased feed conversion efficiency. Its etiologic agent is the avian infectious bronchitis coronavirus (IBV), which is a *Gamma *coronavirus of the *coronavirus *genus and replicates primarily in the upper respiratory tract, kidney, and oviduct of chickens [1-3].

Knowledge of the interactions between virus and host is critical in order to understand the pathogenesis of viral infection. On the one hand, the virus usurps the biological processes of the host to evade the innate immune response of the host; on the other hand, the host mounts a variety of defensive responses against the viral infection. These virus-host interactions can cause changes in the level of expression of host genes. Alteration of gene expression in the host after infection with coronavirus (CoV) has been investigated mainly with regard to infection with mouse hepatitis virus (MHV) and severe acute respiratory syndrome coronavirus (SARS-CoV) [4]. Limited studies have been performed to analyze host gene expression in response to IBV infection at the transcriptional level using microarray technology [5,6]. However, the altered levels of transcription do not reflect the proteomic changes that follow viral infection completely. Therefore, information about proteome changes in the host following IBV infection may be crucial in order to understand the host response to the virus and viral pathogenesis.

In the post-genome era, proteomic analysis can provide insights into the complexity of virus-host interactions. Proteomic approaches have been utilized to investigate the proteome changes in cells infected *in vitro *with classical swine fever virus [7], infectious bursal disease virus [8], porcine circovirus [9], and SARS-CoV [10]. Moreover, proteomic approaches have been used widely to study the mechanisms of viral infection through the comparative analysis of proteome changes in host tissue in response to infection *in vivo *by Marek's disease virus [11] and yellow head virus [12]. More recently, two-dimensional gel electrophoresis (2-DE) was used to compare the potential effect of several different enveloped RNA virus such as Influenza virus, respiratory syncytial virus (RSV), parainfluenza (PIV) and human metapneumovirus (hMPV) on the host cell proteome [13-16]. In addition, Edward Emmott revealed changes in the cytoplasmic, nuclear and nucleolar proteomes in Vero cells and DF-1 cells infected with IBV using Stable Isotope Labeling by Amino acids in Cell culture (SILAC) technique [17,18]. Study on identification of the incorporated host proteins in purified IBV particles has also been reported [19]. Whereas, no studies have been reported to date of the comparative protein expression profiles of chicken embryonic tissues in response to IBV infection either *in vitro *or *in vivo*.

In the present study, we made use of two-dimensional gel electrophoresis (2-DE) coupled with matrix-assisted laser desorption/ionization time-of-flight tandem mass spectrometry (MALDI-TOF-TOF MS) analysis to observe changes of protein expression in the trachea and kidney tissues of chicken embryos after IBV infection *in ovo*. The results may provide the clues that will increase our understanding of the IBV-host interaction and the pathogenesis of IBV.

## Results

### IBV infection in chicken embryos

**Figure 1:**
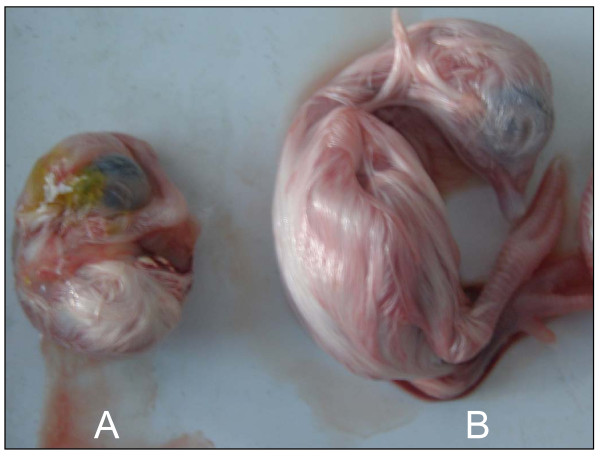
**Pathological characteristics of IBV-infected chicken embryos compared with mock-infected chicken embryos**. (A) The chicken embryos in the IBV-infected group showed obvious sign of IBV infection, such as dwarfing, stunting, curling and embryo death at 72 h after inoculated with IBV. (B) The mock-infected chicken embryos were normal.

Seventy-two hours after inoculation with the IBV vaccine H_120 _strain, all IBV-infected chicken embryos showed obvious signs of IBV infection, such as dwarfing, stunting, curling, and embryonic death. In contrast, the mock-infected chicken embryos were healthy (Figure 1). Analysis of allantoic fluids from embryos in the IBV-infected group by EM showed the presence of virus particles with typical Coronavirus morphology; these were not detected in the mock-infected group (data not shown). Using RT-PCR amplification of the majority of the N gene and parts of the 3'-UTR, the expected 1600bp band was observed only in samples from the IBV-infected group; samples from the mock-infected group were negative, as described previously [20] (data not shown). These results indicated that chicken embryos in the IBV-infected group were infected successfully by IBV.

### Comparison of differential protein expression in trachea and kidney tissues between IBV-infected and mock-infected chicken embryos

**Figure 2:**
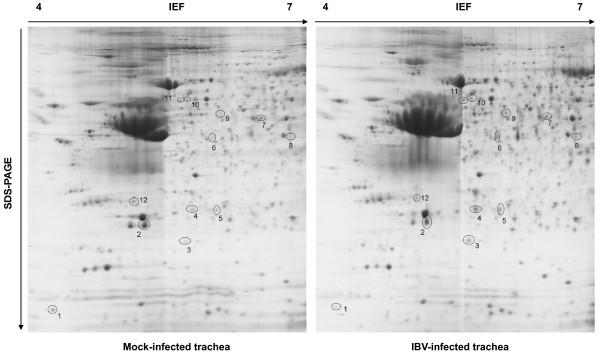
**Analysis using 2-DE of chicken embryo tracheal tissues from the IBV-infected group compared with the mock-infected group using the pH 4-7 range**. Protein samples were separated on 13 cm pH 4-7 IPG strips, followed by SDS-PAGE, and stained with Coomassie Blue R-350. The images were analyzed using Image Master 2D Platinum 6.0 software. The different protein spots identified were marked with a circle and a number. The numbers assigned to the mapped protein spots correspond to the proteins listed in Table 1.

**Figure 3:**
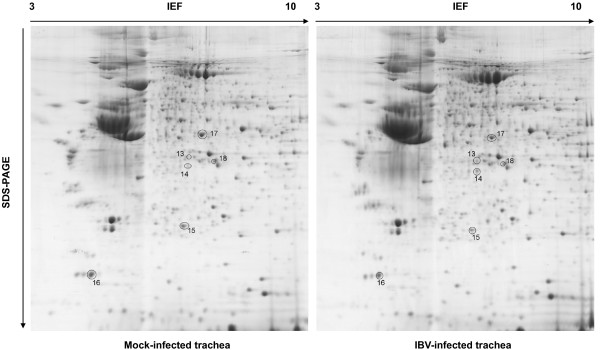
**Analysis using 2-DE of chicken embryo tracheal tissues from the IBV-infected group compared with the mock-infected group using the pH 3-10 range**. Protein samples were separated on 13 cm linear pH 3-10 IPG strips, followed by SDS-PAGE, and stained with Coomassie Blue R-350. The images were analyzed using Image Master 2D Platinum 6.0 software. The different identified protein spots were marked with a circle and a number. The numbers assigned to the mapped protein spots correspond to the proteins listed in Table 1.

**Figure 4:**
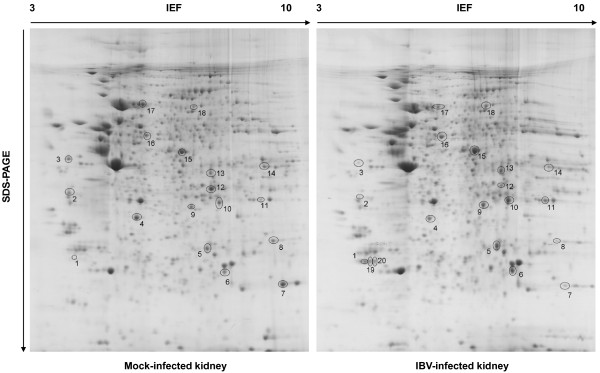
**Analysis using 2-DE of chicken embryo kidney tissues from the IBV-infected group compared with the mock-infected group using the pH 3-10 range**. Protein samples were separated on 13 cm linear pH 3-10 IPG strips, followed by SDS-PAGE, and stained with Coomassie Blue R-350. The images were analyzed using Image Master 2D Platinum 6.0 software. The different identified protein spots were marked with a circle and a number. The numbers assigned to the mapped protein spots correspond to the proteins listed in Table 2.

In order to investigate the proteomic changes in trachea and kidney tissues in response to IBV infection, 2-DE analysis was carried out of the total proteins from trachea and kidney tissues of IBV-infected and mock-infected chicken embryos. Figure 2 shows representative gels of tracheal tissue proteins resolved on 13 cm pH 4-7 IPG strips followed by SDS-PAGE: 1035 ± 47 and 1030 ± 61 protein spots were detected in 2-DE gels from the IBV-infected group and mock-infected group, respectively. Figure 3 shows representative gels of tracheal tissue proteins resolved on 13 cm linear pH 3-10 IPG strips followed by SDS-PAGE: 1248 ± 28 and 1060 ± 18 protein spots were detected in 2-DE gels from the IBV-infected group and mock-infected group, respectively. Thirty protein spots showed statistically significant changes in expression in chicken embryonic tracheal tissues from the IBV-infected group compared with those of the mock-infected group using Image Master Software analysis. Figure 4 shows representative gels of kidney tissue proteins resolved on 13 cm linear pH 3-10 IPG strips followed by SDS-PAGE: 1125 ± 32 and 1074 ± 116 protein spots were detected in 2-DE gels from the IBV-infected group and mock-infected group, respectively. Quantitative analysis revealed that 23 protein spots showed statistically significant changes in expression in chicken embryonic kidney tissues from the IBV-infected group compared with those from the mock-infected group.

**Figure 5:**
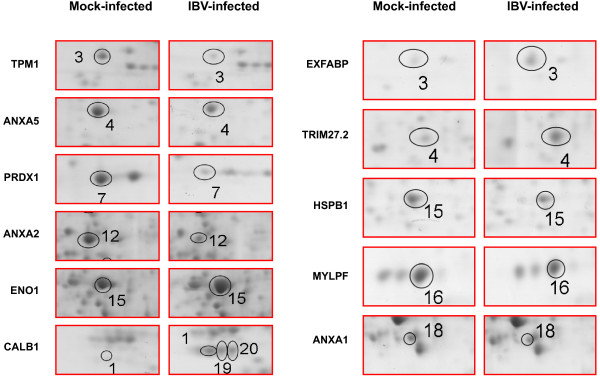
**Comparison of enlarged images of representative differentially expressed protein spots**. The enlarged images of ANXA1, HSPB1, MYLPF, TRIM27.2, EXFABP, ANXA5, PRDX1, TPM1, ENO1, ANXA2 and CALB protein spots are shown. The numbers assigned to the mapped protein spots correspond to the proteins listed in Table 1 or Table 2.

A magnified comparison of eleven differentially expressed protein spots, representing ANXA1, HSPB1, MYLPF, TRIM27.2, EXFABP, ANXA5, PRDX1, TPM1, ENO1, ANXA2 and CALB, is shown in Figure 5.

### Identification of differentially expressed proteins by MALDI-TOF-TOF MS and MS/MS analysis

All protein spots that showed differential changes in expression of 1.5 fold or greater (p < 0.05) were analyzed by MALDI-TOF-TOF MS and MS/MS analysis. The PMF and MS/MS spectral data were searched against the NCBInr database using MASCOT. As shown in Table 1, 17 proteins from tracheal tissues were identified successfully. Proteins increased in abundance included extracellular fatty acid-binding protein (EXFABP), a putative uncharacterized protein (TRIM27.2), ubiquitin carboxyl-terminal esterase L1 (UCHL1), replication factor C (activator 1) 2, a cholinergic receptor (nicotinic, gamma polypeptide precursor), ARP2 actin-related protein 2 homolog, ovotransferrin, a second cholinergic receptor (nicotinic, alpha 7 precursor), chaperonin containing TCP1 subunit 8, and ovoinhibitor precursor. Proteins decreased in abundance included myosin light chain 3 (skeletal muscle isoform), myosin light chain 1 (skeletal muscle isoform), myosin light chain type 2 (MYLPF), heat shock 27 kDa protein (HSPB1), creatine kinase M chain, annexin A1 (ANXA1) and Rho GDP dissociation inhibitor (GDI) alpha. Table 2 shows the 19 proteins identified from kidney tissues. Of these, proteins increased in abundance included carbonic anhydrase II, glutathione S-transferase 2, a protein of the sulfotransferase family, L-lactate dehydrogenase B, L-3-hydroxyacyl-coenzyme A dehydrogenase, cystathionase, enolase 1 (ENO1), CNDP dipeptidase 2, phosphoenolpyruvate carboxykinase, and calbindin-D_28 k _(CALB1). Proteins decreased in abundance included tropomyosin beta chain (TPM2), tropomyosin 1 alpha (TPM1), annexin A5 (ANXA5), peroxiredoxin-1 (PRDX1), carbonyl reductase 1, NADP-dependent isocitrate dehydrogenase, annexin A6 (ANXA6), and MHC class I antigen. Annexin A2 (ANXA2) was identified in two spots (Figure 4, spots 1 and 12). The abundance of spot 1 was increased, but spot 12 was decreased. Calbindin-D_28 k _and MHC class I antigen were detectable only in the IBV-infected group because the spots in the mock-infected group were too weak.

**Table 1:** List of differentially expressed protein spots in tracheal tissues identified by MALDI-TOF-TOF MS and MS/MS analysis

Spot^a^	Accession Number^b^	Protein Description	Mr(KDa)/ p*I*	Score	Coverage (%)^d^	Normalized spot volume (vol%)^e^		*p*	Ratio (infected/mock-infected)	Protein functions	Other viruses found in^f^
										
						Mock-infected	IBV-infected				
*Cytoskeletal and calcium ion-binding proteins*											

1	gi|55584150	Myosin light chain 3, skeletal muscle isoform [*Gallus gallus*]	16.7/4.52	81^c^	38	0.1007 ± 0.0245	0.0452 ± 0.0118	0.024	0.45	Motor activity. Calcium ion binding.	IAV, RSV, CVB3
2	gi|212347	Myosin light chain 1, skeletal muscle isoform [*Gallus gallus*]	19.5/4.96	192	53	0.6040 ± 0.0519	0.3576 ± 0.0656	0.007	0.59	Motor activity. Calcium ion binding.	
16	gi|50403707	myosin light chain type 2 (LC2f) [*Gallus gallus*]	18.9/4.77	220	33	0.4716 ± 0.0545	0.2784 ± 0.0257	0.005	0.59	Motor activity. Calcium ion binding.	VHSV
18	gi|46195459	annexin A1 [*Gallus gallus*]	38.5/7.05	142^c^	39	0.1277 ± 0.0106	0.0596 ± 0.0118	0.002	0.47	Calcium/phospholipid-binding protein. Promotes membrane fusion. Involved in exocytosis. Regulates phospholipase A2 activity. Inflammation response	CSFV, PRRSV, HBV, RSV, VHSV, WNV

*Carbohydrate and lipid metabolic proteins*											

3	gi|20178282	Extracellular fatty acid-binding protein [*Gallus gallus*]	20.1/5.56	361	46	0.0495 ± 0.0094	0.1058 ± 0.0221	0.015	2.14	Fatty acid binding and transporting. Inflammatory response.	
17	gi|45382875	Creatine kinase M chain [*Gallus gallus*]	43.3/6.5	287	50	0.5632 ± 0.0122	0.2546 ± 0.0488	< 0.001	0.45	Nucleotide binding. Catalytic activity. Creatine kinase activity	HBV, VHSV

*Stress response protein*											

15	gi|45384222	Heat shock 27 kDa protein [*Gallus gallus*]	21.7/5.77	166	61	0.2906 ± 0.0637	0.1531 ± 0.0432	0.036	0.53	Response to stress. Anti-apoptosis	CSFV, PRRSV, PCV2, ASFV, AIV, MDV, IBDV, REOV, 1AV, CVB3

*Protein and nucleotide processing*											

5	gi|122692295	ubiquitin carboxyl-terminal esterase L1 [*Gallus gallus*]	25.1/5.74	142	31	0.0563 ± 0.0019	0.0980 ± 0.0071	0.001	1.74	Ubiquitin binding, Protein deubiquitination	DHBV, IBDV
6	gi|45382983	replication factor C (activator 1) 2, 40 kDa [*Gallus gallus*]	40.1/5.68	87^c^	45	0.0542 ± 0.0128	0.1931 ± 0.0297	0.002	3.56	Nucleotide binding. ATP binding	
10	gi|52138673	chaperonin containing TCP1, subunit 8 (theta) [*Gallus gallus*]	59.5/5.35	300	37	0.0403 ± 0.0031	0.0760 ± 0.0047	0.001	1.89	Protein binding. Nucleotide binding	PRRSV, EV71, HPV8, IAV
11	gi|52138673	chaperonin containing TCP1, subunit 8 (theta) [*Gallus gallus*]	59.5/5.35	356	47	0.0559 ± 0.0021	0.1228 ± 0.0375	0.037	2.20	Protein binding. Nucleotide binding	PRRSV, EV71, HPV8, IAV
4	gi|150247116	Putative uncharacterized protein TRIM27.2 (Tripartite motif-containing) [*Gallus gallus*]	27.2/5.25	289^c^	46	0.0792 ± 0.0116	0.2098 ± 0.0196	0.001	2.65	Protein binding. Metal ion binding	
9	gi|45383758	cholinergic receptor, nicotinic, alpha 7 precursor [*Gallus gallus*]	56.9/5.47	159^c^	29	0.0323 ± 0.0055	0.0723 ± 0.0182	0.022	2.24	Acetylcholine receptor activity. Activation of MAPK activity. Cellular calcium ion homeostasis	
7	gi|71896049	cholinergic receptor, nicotinic, gamma polypeptide precursor [*Gallus gallus*]	59.6/5.53	142^c^	26	0.0701 ± 0.0132	0.1207 ± 0.0039	0.003	1.72	Nicotinic acetylcholine-activated cation-selective channel activity. Ion channel activity	
8-1	gi|45382569	ARP2 actin-related protein 2 homolog [*Gallus gallus*]	45.0/6.3	157^c^	51	0.0747 ± 0.0027	0.1336 ± 0.0187	0.006	1.79	ATP binding. Actin binding. Protein binding	
13	gi|71895337	ovoinhibitor precursor [*Gallus gallus*]	54.4/6.16	128^c^	39	0.0334 ± 0.0139	0.1046 ± 0.0364	0.034	3.13	Serine-type endopeptidase inhibitor activity. Peptidase inhibitor activity.	
14	gi|71895337	ovoinhibitor precursor [*Gallus gallus*]	54.4/6.16	291	40	0.0545 ± 0.0228	0.1074 ± 0.0119	0.029	1.97	Serine-type endopeptidase inhibitor activity. Peptidase inhibitor activity.	

*Signal transduction*											

12	gi|124249432	Rho GDP dissociation inhibitor (GDI) alpha [*Gallus gallus*]	23.3/5.22	264	60	0.1091 ± 0.0127	0.0619 ± 0.0131	0.011	0.57	Rho GDP-dissociation inhibitor activity. Signal transduction	CSFV, RSV, WSSV, YHV, IBDV

*Metal ion binding*											

8-2	gi|17942831	Chain A, Ovotransferrin, C-Terminal Lobe, Apo Form	39.4/6.31	93^c^	49	0.0747 ± 0.0027	0.1336 ± 0.0187	0.006	1.79	Metal ion binding. Iron ion transport	

a) Spot ID: is the unique number which refers to the labels in Figure 2 and Figure 3

b) Accession Number: gi number in NCBI.

c) Score: Protein score based only on MS spectra by MALDI-TOF, other spots based on combined MS and MS/MS spectra from MALDI-TOF-TOF identification, a protein score greater than 83 is significant in this study (p < 0.05).

d) Coverage (%): Percentage of identified protein sequences covered by matched peptides.

e) Values are presented as mean ± SD. n = 3; vol % was defined as the ratio of the intensity volume of each spot to that of all spots calculated by the software. The SD represents standard deviation of the vol % in three biological replicates.

f) IAV, Influenza A virus; RSV, Human respiratory syncytial virus; CVB3, coxsackievirus B3; VHSV, Viral haemorrhagic septicemia virus; CSFV, classical swine fever virus; PRRSV, Porcine reproductive and respiratory syndrome virus; PCV2, Porcine circovirus type 2; ASFV, African swine fever virus; HBV, Hepatitis B virus; AIV, Avian influenza virus; EV71, Enterovirus 71; MDV, Marek's disease virus; WSSV, White spot syndrome virus; DHBV, Duck heptatitis B virus; YHV, yellow head virus; HPV8, Human papillomavirus type 8; REOV, Reovirus; IBDV, Infectious bursal disease virus.

**Table 2:** List of differentially expressed protein spots in kidney tissues identified by MALDI-TOF-TOF MS and MS/MS analysis

Spot^a^	Accession Number^b^	Protein Description	Mr(KDa)/ *p*I	Score	Coverage (%)^d^	Normalized spot volume (vol%)^e^		*p*	Ratio (infected/mock-infected)	Protein function	Other viruses found in^f^
										
						Mock-infected	IBV-infected				
*Cytoskeletal proteins*											

2	gi|515694	Tropomyosin beta chain [*Gallus gallus*]	28.5/4.65	123	31	0.2134 ± 0.0092	0.0524 ± 0.0162	< 0.001	0.25	Actin binding	HBV, HCV, WSSV
3	gi|45382323	Tropomyosin 1 alpha [*Gallus gallus*]	32.9/4.73	307	57	0.1907 ± 0.0219	0.0474 ± 0.0188	0.001	0.25	Actin binding	PRRSV, RSV, HPV8, VHSV, CVB3

*Calcium ion-binding proteins*											

1	gi|45382533	annexin A2 [*Gallus gallus*]	38.7/6.92	389	67	0.0388 ± 0.0177	0.2153 ± 0.0838	0.023	5.55	Phospholipase inhibitor activity. Calcium ion binding	CSFV, PRRSV, HBV, HIV, DHBV, WNV
12	gi|45382533	annexin A2 [*Gallus gallus*]	38.6/6.92	505	69	0.5186 ± 0.1260	0.1549 ± 0.0474	0.009	0.3	Phospholipase inhibitor activity. Calcium ion binding	
4	gi|71895873	annexin 5 [*Gallus gallus*]	36.2/5.6	402	71	0.4276 ± 0.0516	0.1898 ± 0.0334	0.003	0.44	Calcium ion binding. Calcium-dependent phospholipid binding	PRRSV, DV, DHBV, HBV, VHSV
17	gi|50982399	annexin A6 [*Gallus gallus*]	75.2/5.57	441	49	0.4317 ± 0.0389	0.1653 ± 0.0107	< 0.001	0.38	Calcium ion binding. Calcium-dependent phospholipid binding	
19	gi|45382893	calbindin 1, 28 kDa [*Gallus gallus*]	30.4/4.72	212	60	N/A	0.1485 ± 0.0614	0.014	N/A	Calcium ion binding. Vitamin D binding	
20-1	gi|45382893	calbindin 1, 28 kDa [*Gallus gallus*]	30.2/4.72	285	50	N/A	0.1527 ± 0.0545	0.008	N/A	Calcium ion binding. Vitamin D binding	

*Carbohydrate and lipid metabolic proteins*											

5	gi|46048696	carbonic anhydrase II [*Gallus gallus*]	29.4/6.56	354	75	0.2622 ± 0.0554	0.4806 ± 0.0850	0.020	1.83	Morphogenesis of an epithelium. One-carbon metabolic process	
8	gi|71895267	carbonyl reductase 1 [*Gallus gallus*]	30.5/8.5	357	82	0.1847 ± 0.0160	0.0909 ± 0.0220	0.004	0.49	Catalytic activity. Oxidoreductase activity	VHSV
10	gi|45383766	L-lactate dehydrogenase B [*Gallus gallus*]	36.3/7.07	306	40	0.3102 ± 0.0260	0.6368 ± 0.1152	0.009	2.05	Glycolysis. Oxidoreductase activity, acting on the CH-OH group of donors, NAD or NADP as acceptor	PRRSV, HBV, HIV, IBDV, REOV
11	gi|118090053	similar to L-3-hydroxyacyl-Coenzyme A dehydrogenase, short chain [*Gallus gallus*]	34.4/8.68	135	39	0.0814 ± 0.0048	0.2186 ± 0.0820	0.044	2.69	Catalytic activity. Oxidoreductase activity. Fatty acid metabolic process	
14	gi|118093509	PREDICTED: similar to cytosolic NADP-dependent isocitrate dehydrogenase [*Gallus gallus*]	46.6/8.02	412	47	0.2077 ± 0.0198	0.0942 ± 0.0092	0.001	0.45	Oxidoreductase activity	PRRSV, PCV2, HBV, RSV
15	gi|46048768	enolase 1 [*Gallus gallus*]	47.3/6.17	387	57	0.5718 ± 0.1537	1.2489 ± 0.0439	0.002	2.18	Glycolysis	PRRSV, PCV2, WSSV, HSV-1, RSV, DHBV, HIV, IBDV, HBV, VHSV, WNV, SARS-CoV
18	gi|110591367	Chain A, The Structure Of Chicken Mitochondrial Pepck	67.3/6.55	441	49	0.0866 ± 0.0138	0.1704 ± 0.0253	0.007	1.97	Gluconeogenesis	HBV, SARS-CoV

*Amino acid metabolic proteins*											

6	gi|2981970	glutathione S-transferases 2 [*Gallus gallus*]	25.8/7.0	437	68	0.1877 ± 0.0201	0.4192 ± 0.0767	0.007	2.23	Amino acid metabolic process	HBV
13	gi|118094764	PREDICTED: similar to cystathionase [*Gallus gallus*]	43.9/6.86	399	35	0.1704 ± 0.0390	0.3164 ± 0.0547	0.02	1.86	Cysteine biosynthetic process	

*Antioxidative stress proteins*											

7	gi|50751518	PREDICTED: similar to peroxiredoxin-1 [*Gallus gallus*]	22.3/8.24	252	42	0.5474 ± 0.0371	0.1253 ± 0.0600	< 0.001	0.23	Response to oxidative stress. Removal of superoxide radicals. Regulation of stress-activated MAPK cascade	CSFV, PRRSV, RSV, SARS-CoV, HBV, IAV

*Protein processing*											

16	gi|57530409	CNDP dipeptidase 2 [*Gallus gallus*]	53.1/5.71	403	52	0.1743 ± 0.0319	0.3196 ± 0.0759	0.038	1.83	Proteolysis	
9	gi|45382969	sulfotransferase family, cytosolic, 1C, member 3 [*Gallus gallus*]	36.2/6.68	245	47	0.1481 ± 0.0412	0.2923 ± 0.0578	0.024	1.97	Sulfotransferase activity. Detoxicating	

*Immune response proteins*											

20-2	gi|54606655	MHC class I antigen [*Gallus gallus*]	37.6/6.09	385^c^	71	N/A	0.1527 ± 0.0545	0.008	N/A	Immune response. Antigen processing and presentation	BVDV

a) Spot ID: is the unique number which refers to the labels in Figure 4.

b) Accession Number: gi number in NCBI.

c) Score: Protein score based only on MS spectra by MALDI-TOF, other spots based on combined MS and MS/MS spectra from MALDI-TOF-TOF identification, a protein score greater than 83 is significant in this study (P < 0.05).

d) Sequence coverage (%): Percentage of identified protein sequences covered by matched peptides.

e) Values are presented as mean ± SD. n = 3; vol % was defined as the ratio of the intensity volume of each spot to that of all spots calculated by the software. The SD represents standard deviation of the vol % in three biological replicates.

N/A: A indicates the spot was detectable on one of the gels, N indicates the spot was too weak to detect on one of the gels.

f) HCV, Hepatitis C virus; VSV, Vesicular stomatitis virus; HSV-1, Herpes simplex virus type-1; DV, Dengue virus; HIV, Human immunodeficiency virus; SARS-CoV, Severe acute respiratory syndrome -associated coronavirus; VHSV, Viral haemorrhagic septicemia virus; WNV, West Nile virus; BVDV, Bovine viral diarrhea virus.

According to the UniProtKB and the Gene Ontology databases, the identified proteins could be classified into several functional categories, including cytoskeletal proteins, calcium ion-binding proteins, proteins related to macromolecule metabolism, anti-oxidative proteins, protein and nucleotide processing, the ubiquitin-proteasome pathway, immune response and antigen processing and presentation, response to stress, signal transduction, and metal ion binding. Detailed information about the PMF and MS/MS search results is listed in Additional file 1, Additional file 2, Additional file 3 and Additional file 4.

### Analysis of identified proteins at the transcriptional level

**Figure 6:**
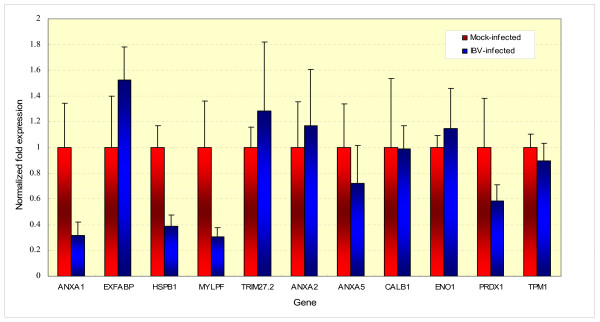
**Transcript alteration of eleven selected genes in chicken embryo tissues from the IBV-infected group compared with the mock-infected group**. Total RNA extracted from tracheal or kidney tissues was measured by real-time RT-PCR analysis; relative expression levels were calculated according to the 2^-ΔΔCT ^method, using GAPDH as an internal reference gene and the mock-infected group as calibrator (relative expression = 1). Error bar shows the standard deviation. Gene symbols indicating different genes refer to Table 1 or Table 2.

Alterations in expression of a protein may be due to a change in its mRNA level. In order to confirm the results of the proteomics analysis at the mRNA level, the transcriptional alterations in five selected proteins from tracheal tissues and six selected proteins from kidney tissues were measured by real-time RT-PCR. Figure 6 shows normalized fold changes of the mRNA of these genes in chicken embryos of the IBV-infected group and in mock-infected chicken embryos. In tracheal tissues, the mRNA level of ANXA1, HSPB1, and MYLPF was decreased in IBV-infected chicken embryos by 0.32, 0.39, 0.31 fold, respectively, compared with mock-infected chicken embryos. The mRNA level of TRIM27.2 and EXFABP was increased by 1.28 and 1.52 fold, respectively, and the trends of the changes in their mRNA levels were similar to the patterns of change in their corresponding proteins on 2-DE gels. For kidney tissues, the trends of change in the mRNA levels of ANXA5, PRDX1, TPM1, and ENO1 were consistent with the 2-DE results. Interestingly, ANXA2 had results that contrasted with those of the 2-DE methods. CALB1 was found to show no obvious difference between the IBV-infected group and the mock-infected group.

### Protein validation by Western blotting

**Figure 7:**
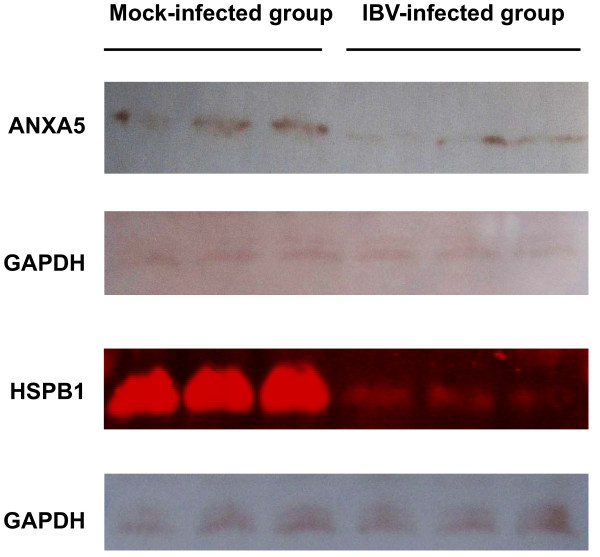
**Western blotting analysis confirmation of representative protein in IBV-infected and mock-infected chicken embryo tissues**.

To further confirm the protein alterations during IBV infection identified by 2-DE and MALDI-TOF/TOF mass spectrometry, the protein annexin A5 and HSPB1 were selected for Western blotting analysis and GAPDH as loading control. As shown in Figure 7, the abundance of annexin A5 was decreased in kidney tissues of IBV-infected group compared to mock-infected group, and the abundance of HSPB1 was decreased in tracheal tissues of IBV-infected group compared to mock-infected group. The results were consistent with the expression change shown by the 2-DE analysis.

## Discussion

Virus infection and the host response involve a complex interplay of host and viral networks in which many viruses attempt to subvert host cell processes to increase the efficiency of virus infection, and likewise the host employs a number of responses to generate an anti-viral state [17]. Coronavirus (CoV) infection can cause alterations in the transcription and translation patterns, cell cycle, cytoskeleton, and apoptosis pathways of the host cell [4]. The trachea and kidney are the primary target organs of IBV, investigation of the proteomic changes in these tissues after IBV infection *in ovo *helps to elucidate the IBV-host interaction and the pathogenic mechanisms of IBV. In this study, proteomic methods coupled with real-time RT-PCR and western blotting were applied to identify the differentially expressed proteins in trachea and kidney tissues of IBV-infected and mock-infected chicken embryos. We now attempt to interpret the possible functional roles of some proteins identified during IBV infection *in ovo*.

In our study, one of the major findings was that the abundances of some cytoskeletal proteins including TPM1 and MYLPF were decreased in the IBV-infected group. Their alterations were also confirmed at the mRNA level by real-time RT-PCR. Tropomyosin belongs to the family of actin-binding proteins that serves important functions in microfilament stabilization, regulation of microfilament branching, actin polymerization, and intracellular transport [21]. Myosins are a large superfamily of motor proteins that are involved in movement along actin filaments, the development of myriad cells, targeted organelle transport, endocytosis, chemotaxis, cytokinesis, and signal transduction [22]. Similar result was observed in the IBV-infected cells by using SILAC technique [17,18]. Changes in cytoskeleton proteins have been reported in other virus infection *in vitro*, including infectious bursal virus [8], H9N2 avian influenza virus [23], respiratory syncytial virus [16], and SARS-associated CoV [10]. During the process of virus infection, particularly in the stages of virus entry and virus budding, the cytoskeletal network of the host cell is involved in the transport of viral components within the cell. Moreover, some viral proteins can interact with the cytoskeletal transport machinery, such as actin-binding proteins or actin, and induce rearrangements of cytoskeletal filaments so that they can utilize them as tracks or push them aside when they represent barriers [24]. In the present study, several actin-binding proteins, including TPM1 and MYLPF, their abundance were found to be decreased in the IBV-infected group, which suggests that IBV may also manipulate the host cytoskeletal network for its own infectious processes and replication.

It is well known that Ca^2+ ^is one of the most universal and versatile signaling molecule, and involved in almost every aspect of cellular processes. The Ca^2+ ^plays important roles in virus entry, viral gene expression, posttranslational processing of viral proteins, and the maturation and release of virions. Viruses can utilize host cellular Ca^2+ ^and Ca^2+^-binding proteins to create a tailored cellular environment that meets their own demands for the replication cycle [25]. In this study, the level of expression of some calcium ion-binding proteins, including Calbindin-D_28 k_, annexin A1, annexin A2, annexin A5, and annexin A6 were altered after IBV infection *in ovo*. Calbindin-D_28 k _is a cytosolic calcium-binding protein that facilitates 1, 25 (OH)_2_D_3 _dependent transcellular calcium transport. It was also observed to protect against apoptosis in different cell types [26,27]. In this study, its abundance was remarkably increased in the IBV-infected group, which suggests that IBV might specially utilize calbindin-D_28 k _to perturb the cellular Ca^2+ ^homeostasis and Ca^2+^-signaling network for its own benefit. Annexins are a family of structurally related proteins that bind phospholipids and cellular membranes in a calcium-dependent manner [28]. Annexin A2 has been shown to take part in the initiation of membrane fusion in exocytosis, membrane trafficking, regulation of cell proliferation and apoptosis, and stabilization of membrane-associated protein complexes with the actin cytoskeleton [29,30]. In addition, Annexin A2 can promote the entry of human immunodeficiency virus (HIV) into monocyte-derived macrophages [29], and it was also identified to be a potential receptor for respiratory syncytial virus on human epithelial cells [31]. Annexin A2 on the lung epithelial cell surface was recognized by SARS-associated CoV spike domain 2 antibodies and identified as an autoantigen [32]. Annexin A5 was found to be involved in cytomegalovirus infection [33] and influenza virus infection [34]. Annexin A1 plays a critical role in a variety of cellular processes such as proliferation, differentiation, and apoptosis [35]. Its abundance was shown to be increased in HepaRG cells infected with hepatitis B virus (HBV) *in vitro *[36], and fish cells infected *in vitro *with infectious pancreatic necrosis virus [37]. Changes in the abundance of some annexins family proteins also were identified in IBV-infected DF-1 cells by Edward Emmott and co-workers [18]. In current study, the abundance of annexin A1, annexin A5, and annexin A6 were all decreased in the IBV-infected group. For annexin A2, two spots were identified in kidney tissue, the abundance of one spot was increased, and another spot was decreased. Of these, the decrease of annexin A5 was confirmed by real-time RT-PCR and western blotting analysis. These data suggested that they may play special roles during IBV infection or replication.

Remarkably, several stress response and anti-oxidative proteins were found to be changed significantly in the present study. HSPB1 is an important small heat shock protein (HSP) that is synthesized in response to a wide variety of stressful stimuli, including viral infection. It has diverse functions including chaperone activity, F-actin modulation, signal transduction, resistance to oxidant stress, regulation of translational initiation, and modulation of inflammation, inhibition of apoptosis, and cell differentiation and proliferation [38,39]. Enhanced levels of HSPB1 and/or phosphoHSPB1 can promote nuclear transport of adenovirus in MK2-deficient cells [40]. The abundance of HSPB1 has found to be increased in cells infected *in vitro *with H9N2 avian influenza virus [23], African swine fever virus [41], and infectious bursal disease virus [8]. In contrast, its abundance was found to be decreased in cells infected *in vitro *with mumps virus [42] and porcine circovirus type 2 [9], which suggests that HSPB1 may play different roles in different virus infections or different stages of infection. PRDX1 is the most ubiquitously expressed member of the peroxiredoxin family, which is involved in anti-oxidative processes, cell differentiation and proliferation, immune responses, regulation of apoptosis, and as a molecular chaperone [43]. PRDX1 participates in the apoptosis signal-regulating kinase 1 (ASK1)-mediated signaling pathway, and plays an inhibitory role in ASK1-induced apoptosis [44]. Its abundance was shown to be decreased in peripheral blood mononuclear cell (PBMC) following CSFV infection *in vivo *[45]. In our study, the abundance of HSPB1 and PRDX1 were shown to be decreased after IBV infection *in ovo *by 2-DE and real-time RT-PCR methods. Furthermore, the change of HSPB1 expression was confirmed by western blotting. This alteration may allow the infected cells to be eliminated by apoptosis, or serve as a form of host defense against IBV infection.

Viral replication requires energy and macromolecular precursors derived from the metabolic network of the host. In the present results obtained using 2-DE, the abundance of some proteins which are associated with carbohydrate, amino acid, and lipid metabolic processes were found to be differentially changed. Enolase-1 is a key enzyme of glycolysis and gluconeogenesis that catalyzes the dehydration of 2-phosphoglycerate to phosphoenolpyruvate [46]. Its abundance was found to be changed in many virus infections, such as white spot syndrome virus [47], and porcine reproductive and respiratory syndrome virus [48]. Phosphoenolpyruvate carboxykinase is another gluconeogenic enzyme; it catalyzes the GTP-driven conversion of oxaloacetate to phosphoenolpyruvate [49]. Increased expression of proteins related to energy metabolism was also found in HIV-infected peripheral blood mononuclear cells [50], chicken spleen tissue infected with Marek's disease virus [11], and human cytomegalovirus-infected human fibroblasts [51]. The abundance of L-lactate dehydrogenase B also found to be increased in IBV-infected cells by SILAC technique [17,18]. Up-regulation of proteins related to energy metabolism may meet the requirement for a large burst of oxygen and energy during rapid virus replication, and also may result from an attempt by the host to keep up with the energy demand during viral infection. Ex-FABP is 21 kDa lipocalin that is involved in fatty acid transport and lipid metabolism. It may play an important role in the protection of cells against the toxic effects of the accumulation of fatty acids. The expression of Ex-FABP is enhanced greatly in response to inflammatory stimuli and other stress conditions [52,53]. In this study, its abundance was significantly increased in tracheal tissue of IBV-infected chicken embryos, suggesting may serve as a response to the inflammation induced by IBV infection *in ovo*.

The abundance of several proteins which involved in the immune response and antigen processing and presentation were also observed to be changed in this study. TRIM protein is a member of a protein family that is based on a conserved domain architecture characterized by a RING finger domain, one or two B-box domains, a coiled-coil domain and a variable C-terminus. TRIM proteins are involved in a variety of cellular processes that include signal transduction, transcriptional regulation, cell proliferation, apoptosis, and immunity [54]. Many TRIM proteins, such as TRIM5α, TRIM11, TRIM22, TRIM28, TRIM31, and TRIM62, have been found to display antiviral activity or to be involved in processes associated with innate immunity [55-57]. An extended gene map revealed that TRIM27.2 lies within a sub-region of the chicken MHC-B that affects infectious disease [58]. The major histocompatibility complex (MHC) plays an important role in regulation of the immune response and antigen presentation. The transcription levels of the MHC class II-associated invariant chain and MHC class II β chain were observed to be increased in tracheal epithelial layers of chickens three days after infection with an attenuated IBV-Massachusetts strain [6]. In current study, the abundance of TRIM27.2 and MHC class I antigen were increased remarkably following IBV infection *in ovo*. According to our knowledge, TRIM27.2 has never been found in other virus analysis so far. This change might be induced specially by IBV infection.

## Conclusions

In summary, we have performed the first analysis of the proteomic changes in chicken embryonic trachea and kidney tissues during IBV infection *in ovo*. We identified a series of proteins that are related mainly to the cytoskeleton, calcium ion binding, the stress response, anti-oxidative stress, and macromolecular metabolism. Notably, some of the identified proteins have the ability to regulate apoptosis. Our study should facilitate a better understanding of the pathogenic mechanisms of IBV infection. Future work will focus on the analysis of the specific roles of some interesting proteins during IBV infection.

## Materials and methods

### Experimental animals and virus infection

Eighteen 13-day-old SPF chicken embryos (Harbin Veterinary Research Institute, China) were divided randomly into two groups, the IBV-infected group and the mock-infected group (nine chicken embryos in each group). Each chicken embryo in the IBV-infected group was inoculated with 100 μl chorioallantoic fluid containing IBV H_120 _strain (10^5^-10^6^EID_50_). As a control, each chicken embryo in the mock-infected group was inoculated in parallel with 100 μl sterile chorioallantoic fluid. The inoculated chicken embryos were incubated at 37°C and candled daily to check for embryonic viability.

Seventy-two hours after inoculation, the inoculated embryos were examined for characteristics of IBV infection. All the allantoic fluid was harvested from the IBV-infected and mock-infected chicken embryos to test for the presence of IBV using electron microscopy (EM), and for reverse transcriptase-polymerase chain reaction (RT-PCR) amplification of the majority of the N gene and parts of the 3'-UTR, as described previously [3,59]. Trachea and kidney tissues were removed quickly from the chicken embryos. Tissue samples from three randomly selected chicken embryos per group were pooled, and the pooled tissue samples were stored immediately at -80°C for 2-DE and real-time RT-PCR analysis.

### Protein sample preparation

The frozen tissues were rinsed in ice-cold PBS buffer, then placed in liquid nitrogen and ground thoroughly to a very fine powder. Tissue powder (100 mg) was dissolved in 500 μl lysing solution containing 7 M urea, 2 M thiourea, 4% CHAPS, 40 mM DTT, 2% IPG buffer pH 3-10 or pH 4-7, 1% Nuclease Mix and 1% Protease Inhibitor Mix (GE Healthcare), then incubated for 2 h at room temperature with vortexing once every 15 min, and centrifuged at 15 000 ×g for 1 h at 4°C. The supernatant was collected and purified with the PlusOne 2D Clean-up kit (GE Healthcare). The concentration of each protein sample was determined with the PlusOne 2D Quant Kit (GE Healthcare). Protein samples were aliquoted and stored at -80°C for 2-DE analysis.

### Two-dimensional electrophoresis

Three independent sample pools of each kind of tissue per group were used for 2-DE analysis respectively. For tracheal tissue samples, pH 4-7 and linear pH 3-10 IPG strips were used. For kidney tissue samples, only linear pH 3-10 IPG strips were used. Briefly, 400 μg protein (for pH 3-10 strips) or 550 μg (for pH 4-7 strips) were added to the rehydration solution (7 M urea, 2 M thiourea, 40 mM DTT, 2% CHAPS, 0.5% pH 3-10 or pH 4-7 IPG buffer, and 0.002% bromophenol blue) to make the final volume up to 250 μl, then loaded onto 13 cm, pH 4-7 or linear pH 3-10 IPG strips (GE Healthcare). Isoelectric focusing (IEF) was performed on an Ettan IPGphor 3 (GE Healthcare) using the following procedure: 12 h at 30 V, 30 min at 200 V, 2 h at 500 V, 1 h at 1000 V, 2 h of a linear gradient from 1000 V to 7000 V, and 7000 V to 55000 Vh. After IEF, the IPG strips were first equilibrated with gentle shaking for 15 min in an equilibration buffer containing 6 M urea, 50 mM Tris-HCl, pH 8.8, 30% glycerol, 2% SDS, 0.002% bromophenol blue, and 1% DTT, then for an additional 15 min in the same buffer except that the DTT was replaced by 2.5% iodoacetamide. For SDS-PAGE, the IPG strips were placed on 12.5% SDS-polyacrylamide gels, and electrophoresis was carried out using an SE600 Ruby system (GE Healthcare). The gels were stained with PlusOne Coomassie Blue R-350 (GE Healthcare) according to the manufacturer's protocol, and destained with 10% acetic acid solution.

### Image acquisition and analysis

The stained 2-DE gels were scanned with an ImageScanner III (GE Healthcare). Spot detection, matching and quantification analyses were performed with Image Master 2D Platinum software v6.0 (GE Healthcare). For image analysis, three independent gels from the different sample pools for each group were compared. Protein spot intensity was defined as the normalized spot volume (vol %), then the normalized volume values (vol %) of individual protein spots were subjected to Student's *t *test using SPSS statistical software package version 16.0. The criterion used to define differential expression of spots was that the ratio of the vol % in the IBV-infected group vs. the mock-infected group was more than 1.5 (p < 0.05) or less than 0.67 (p < 0.05). Differentially expressed protein spots were subjected to MS analysis.

### Protein identification by MALDI-TOF-TOF MS and MS/MS analysis

The differentially expressed protein spots were excised manually from the gels, dehydrated in acetonitrile (ACN), and dried at room temperature. Proteins were reduced with 10 mM dithiothreitol (DTT) /25 mM NH_4_HCO_3 _at 56°C for 1 h and alkylated with 55 mM iodoacetamide/25 mM NH_4_HCO_3 _in the dark at room temperature for 45 min *in situ*. Gel pieces were washed thoroughly with 25 mM NH_4_HCO_3_, 50% and 100% ACN, and dried in a Speedvac. Dried gel pieces were rehydrated with 2-3 μl of trypsin (Promega) solution (10 ng/μl in 25 mM ammonium bicarbonate) at 4°C for 30 min. Excess liquid was discarded and the gel plugs were incubated at 37°C for 12 h. Trifluoroacetic acid (TFA) was added to a final concentration of 0.1% to stop the digestive reaction.

The digests were spotted immediately onto 600 μm Anchorchips (Bruker Daltonics). Spotting was achieved by pipetting 1 μl of analyte onto the MALDI target plate in duplicate, then adding 0.05 μl of 2 mg/ml α-cyano-4-hydroxycinnamic acid in 0.1% TFA/33% ACN, which contained 2 mM ammonium phosphate. Bruker peptide calibration mixture was spotted down for external calibration. All samples were allowed to air dry at room temperature, and 0.1% TFA was used for on-target washing. All samples were analyzed in the positive-ion, reflectron mode, on a TOF Ultraflex II mass spectrometer (Bruker Daltonics).

Each acquired mass spectrum (m/z range 700-4000, resolution 15 000-20 000) was processed using the Flex Analysis software version 2.4 (Bruker Daltonics) with the following settings: peak detection algorithm set at SNAP (Sort Neaten Assign and Place), S/N threshold at 3, and Quality Factor Threshold at 50. The trypsin autodigestion ion picks (842.51, 1045.56, 2211.10, and 2225.12 Da) were used as internal standards to validate the external calibration procedure. Matrix and/or auto-proteolytic trypsin fragments, and known contaminants (e.g., keratins) were removed. The resulting peptide mass lists were used first to search the NCBInr database (release 16/01/2010, 10343571 sequences, 3528215794 residues) for Gallus sequences with MASCOT (v2.3) in the automated mode; the following parameters were used as criteria in the search: significant protein MOWSE score at 83 (p < 0.05), minimum mass accuracy at 100 ppm, trypsin as enzyme, one missed cleavage site allowed, cysteine carbamidomethylation, acrylamide modified cysteine, methionine oxidation, and the minimum sequence coverage at 15%. The samples identified by PMF were automatically submitted to MS/MS analysis. Three of the strongest peaks of the TOF spectra per sample were chosen for MS/MS analysis. For MS/MS spectra searching, the spectra were used to search the NCBInr database (release 16/01/2010, 10343571 sequences, 3528215794 residues) for Gallus sequences using MASCOT (v2.3). The search parameters for MS/MS data included 100 ppm for the precursor ion and 0.4 Da for the fragment ions. Cleavage specificity and covalent modifications were considered as described above, and the score was higher than the minimal significant (p < 0.05) individual ion score. All significant MS/MS identifications by MASCOT were verified manually for spectral quality and matching of the y and b ion series.

### Real-time RT-PCR

Specific primers were designed according to the corresponding gene sequences of MS-identified proteins using Beacon Designer software 7.5 (Primer Biosoft International). All the information on the primers is listed in Table 3. Total RNA was extracted using TRIzol Reagent (Invitrogen) according to the manufacturer's instructions. The concentration and purity of the RNA were measured using a spectrophotometer (GE Healthcare). Two micrograms of total RNA was reverse transcribed with 200 U M-MLV Reverse Transcriptase (Invitrogen) and 500 ng Oligo(dT)_18 _as the first strand primer in 20 μl reaction solution. Real-time PCR was carried out on the iCycler^® ^real-time PCR detection system (Bio-Rad Laboratory). Each 25 μl reaction volume contained 1 μl 10 μM (each) forward and reverse primers, 12.5 μl 2 × SYBR^® ^Premix Ex Taq™ II (Takara), and 2 μl 1:10 diluted cDNA products, and the final volume was adjusted using PCR-water. The following PCR program was used for amplification: 30 s at 95°C, 40 cycles of denaturation at 95°C for 10s, and annealing and extension at 55°C for 30 s. Three independent sample pools per group were analyzed. Quantitative analysis of the data was performed using the iCycler IQ^5 ^optical system software version 2.0 (Bio-Rad Laboratory) in a Normalized Expression (ΔΔCt) model, using the mock-infected group as calibrator (relative expression = 1) and GAPDH as an internal reference gene.

**Table 3:** The primers used for real-time RT-PCR

Gene symbol	Gene accession No.	Forward primer sequence (5'-3')	Reverse primer sequence (5'-3')	Amplicon size (bp)
ANXA2	NM_205351	CTGTGATTGACTATGAACTGATTG	TTAACTTCCTTCTTGATGCTCTC	196
ANXA5	NM_001031538	GGCTGGCACTGATGATGATACC	CCACCACAGAGGAGCAGGAG	171
PRDX1	XM_001233859	CTGCTGGAGTGCGGATTG	AGAGGGTAGAAGAAGAACACAAC	186
TPM1	NM_205401	CATTGCTGAAGAGGCTGAC	CGGACTTGGCTTTCTGATAG	114
CALB1	NM_205513	AGGCAGGCTTGGACTTAAC	GCTGGCACCTAAAGAACAAC	141
ENO1	NM_205120	AATGGATGGAACGGAGAAC	AGCAAGGTCAGCAATGTG	127
ANXA1	NM_206906	GGACAACCAGGAGCAGGAATG	TGGCTTCATCTACACCCTTTACAG	134
HSPB1	NM_205290	CTGGTGGTGAAGACTAAGGATAAC	GGGTGTATTTGCGGGTGAAG	106
MYLPF	M11030.1	CCTCCAATGTCTTCTCTATG	TCCAACTCCTCGTTCTTC	160
TRIM27.2	NM_001099359	GCAAGCACTGAAGGAAGAC	AGCCAGCAGGTGATGTTC	166
EXFABP	NM_205422	GCTGGACACGGACTACAAGAG	GCTCACCTCACGGCTTCTG	106
GAPDH	AF047874	GTGAAGGCTGCTGCTGATG	AGGTGGAGGAATGGCTGTC	100

### Western blotting analysis

Samples from IBV-infected and mock-infected chicken embryo kidney tissues and tracheal tissues were lysed and protein concentrations were determined as described above. Equivalent amounts of total protein were subjected to 12% SDS-PAGE and then transferred to nitrocellulose membrane. After blocking for one hour at 37°C, the membranes were incubated with mouse monoclonal antibody to annexin A5 (sc-32321, Santa Cruz Biotechnology, USA) and mouse monoclonal antibody to HSP27 (sc-51956, Santa Cruz Biotechnology, USA) for overnight at 4°C. The membranes were then separately incubated with horseradish peroxidase (HRP)-conjugated anti-mouse IgG (A2554, Sigma, USA) or IRDye700DX conjugated anti-mouse secondary antibody (610-130-121, Rockland, Gilbertsville, PA) for one hour at room temperature, finally visualized using 3,3-diaminobenzidine tetrahydrochloride (DAB) as the substrate or scanned on a LI-COR infrared imaging system using their Odyssey software (Li-Cor Bioscience, Lincoln, NE). HRP-conjugated monoclonal mouse anti-GAPDH (ab9482, Abcam, USA) was used as reference protein to check equal loading. Triplicates were performed.

## Abbreviations

ACN: acetonitrile; CHAPS:, 3-[(3-cholamidopropyl) dimethyl-ammonio] -1-propanesulfonate; DTT: dithiothreitol; GAPDH: glyceraldehyde-3-phosphate dehydrogenase; IBV: Infectious bronchitis coronavirus; IEF: isoelectric focusing; IPG: immobilized pH gradient; MALDI-TOF-TOF/MS: matrix-assisted laser desorption/ionization time-of-flight tandem mass spectrometry; PMF: peptide mass fingerprinting; RT-PCR: reverse transcriptase-polymerase chain reaction;SDS-PAGE: sodium dodecyl sulfate polyacrylamide gel electrophoresis; SPF: specific pathogen free; TFA: trifluoroacetic acid; 2-DE: two-dimensional gel electrophoresis.

## Competing interests

The authors declare that they have no competing interests.

## Authors' contributions

SL designed the study. SL and ZC drafted the manuscript. ZC, ZH and YS carried out virus infection and test for the presence of IBV. ZC and HG carried out the 2-DE experiments, image analysis, excised the protein spots, data analysis and interpretation, and confirmed the differential expression by real-time RT-PCR and Western blotting analysis. SL wrote the manuscript. XK revised the manuscript. All authors read and approved the final manuscript.

## Supplementary Material

Additional file 1 **Additional_file_1.doc containing the MALDI-TOF spectrum and MALDI-TOF-TOF spectrum of differentially expressed protein spots in IBV-infected chicken embryo tracheal tissues**. Click here for file

Additional file 2 **Additional_file_2.doc containing the PMF spectrum and Mascot database search results of differentially expressed protein spots in IBV-infected chicken embryo tracheal tissues**. Click here for file

Additional file 3 **Additional_file_3.doc containing the MALDI-TOF spectrum and MALDI-TOF-TOF spectrum of differentially expressed protein spots in IBV-infected chicken embryo kidney tissues**. Click here for file

Additional file 4 **Additional_file_4.doc containing the PMF spectrum and Mascot database search results of differentially expressed protein spots in IBV-infected chicken embryo kidney tissues**. Click here for file
