# Supplementary material for: Proteomic analysis of chicken embryonic trachea and kidney tissues after infection in ovo by avian infectious bronchitis coronavirus
Source: Proteome Sci. 2011 Mar 8;9:11. doi: 10.1186/1477-5956-9-11 (PMC3060854; doi:10.1186/1477-5956-9-11)
Supplement: Additional file 1 — Additional_file_1.doc containing the MALDI-TOF spectrum and MALDI-TOF-TOF spectrum of differentially expressed protein spots in IBV-infected chicken embryo tracheal tissues. [file 1477-5956-9-11-S1.DOC]

**Additional file 1**

This includes the PMF spectrum and confirmed MALDI-TOF-TOF spectrum of 10 differentially expressed protein spots in IBV-infected chicken embryo tracheal tissues. The MALDI-TOF spectra were given by annotating with masses (blue), and their peptide assignments were tabulated with masses. In tables, the peptides with statistically significant ion score (red and bold) was considered to be confidently identified by MALDI-TOF-TOF, and their MS/MS spectra were shown with masses detected and fragment assignments (red). The precursor mass and charge of peptides are shown in bracket, e.g. (1200.6983).

**Spot No. 2 gi|212347 myosin light chain 1f [Gallus gallus]**

| **Observed Mr** | **Mr(expt)** | **Mr(calc)** | **ppm** | **Start Seq.** | **End Seq.** | **Miss** | **Ion Score** | **Peptide sequence** |
| --- | --- | --- | --- | --- | --- | --- | --- | --- |
| 967.5464 | 966.5391 | 966.550 | -11.08 | 137 | 145 | 0 |  | R.HVLATLGEK.M |
| 1010.5368 | 1009.5295 | 1009.5233 | 6.20 | 40 | 47 | 0 |  | K.EAFLLFDR.T |
| **1200.6983** | **1199.6910** | **1199.6874** | **3.03** | **53** | **63** | **0** | **87** | **K.ITLSQVGDIVR.A** |
| 1200.6983 | 1199.6910 | 1199.6874 | 3.03 | 53 | 63 | 0 |  | K.ITLSQVGDIVR.A |
| 1233.5903 | 1232.5830 | 1232.5819 | 0.89 | 125 | 136 | 0 |  | K.EGNGTVMGAELR.H |
| 1369.7205 | 1368.7133 | 1368.6997 | 9.91 | 64 | 76 | 0 |  | R.ALGQNPTNAEINK.I |
| 1430.7349 | 1429.7276 | 1429.7235 | 2.92 | 77 | 89 | 1 |  | K.ILGNPSKEEMNAK.K |
| 1512.7011 | 1511.6938 | 1511.6893 | 2.99 | 108 | 120 | 0 |  | K.DQGTFEDFVEGLR.V |
| 1672.9082 | 1671.9010 | 1671.9156 | -8.73 | 48 | 63 | 1 |  | R.TGDAKITLSQVGDIVR.A |
| 1722.8471 | 1721.8399 | 1721.8407 | -0.47 | 121 | 136 | 1 |  | R.VFDKEGNGTVMGAELR.H |
| 1900.9082 | 1899.9009 | 1899.9003 | 0.34 | 33 | 47 | 1 |  | K.EQQDDFKEAFLLFDR.T |

**MS/MS Fragmentation of K.ITLSQVGDIVR.A**

**Spot No. 3 gi|45382221 Extracellular fatty acid-binding protein [Gallus gallus]**

| **Observed Mr** | **Mr(expt)** | **Mr(calc)** | **ppm** | **Start Seq.** | **End Seq.** | **Miss** | **Ion Score** | **Peptide sequence** |
| --- | --- | --- | --- | --- | --- | --- | --- | --- |
| 788.3896 | 787.3824 | 787.3833 | -1.19 | 129 | 134 | 0 |  | R.TLHMMR.L |
| 939.4987 | 938.4914 | 938.4862 | 5.57 | 83 | 89 | 1 |  | K.WETTFKK.T |
| 1027.5565 | 1026.5492 | 1026.5498 | -0.62 | 115 | 123 | 0 |  | K.SYAVIFATR.V |
| **1027.6434** | **1026.6362** | **1026.5498** | **84.10** | **115** | **123** | **0** | **61** | **K.SYAVIFATR.V** |
| 1221.6344 | 1220.6271 | 1220.6223 | 3.90 | 141 | 151 | 0 |  | R.EVSPTAMAIFR.K |
| **1221.7441** | **1220.7368** | **1220.6223** | **93.80** | **141** | **151** | **0** | **77** | **R.EVSPTAMAIFR.K** |
| 1349.7341 | 1348.7268 | 1348.7173 | 7.04 | 141 | 152 | 1 |  | R.EVSPTAMAIFRK.L |
| **1349.8563** | **1348.8490** | **1348.7173** | **97.60** | **141** | **152** | **1** | **64** | **R.EVSPTAMAIFRK.L** |
| 1464.7772 | 1463.7699 | 1463.7555 | 9.88 | 139 | 151 | 1 |  | R.SREVSPTAMAIFR.K |
| **1464.9020** | **1463.8947** | **1463.7555** | **95.10** | **139** | **151** | **1** | **66** | **R.SREVSPTAMAIFR.K** |
| 1721.7281 | 1720.7208 | 1720.6951 | 14.90 | 90 | 104 | 0 |  | K.TSDDGEVYYSEEAEK.T |
| 1815.9568 | 1814.9495 | 1814.9355 | 7.69 | 34 | 48 | 0 |  | K.WYIVALASNTDFFLR.E |
| 2191.1252 | 2190.1179 | 2190.1208 | -1.35 | 105 | 123 | 1 |  | K.TVEVLDTDYKSYAVIFATR.V |
| 2387.2367 | 2386.2294 | 2386.2321 | -1.13 | 28 | 48 | 1 |  | R.SEVAGKWYIVALASNTDFFLR.E |
| 2885.2654 | 2884.2581 | 2884.2662 | -2.79 | 90 | 114 | 1 |  | K.TSDDGEVYYSEEAEKTVEVLDTDYK.S |

**MS/MS Fragmentation of K.SYAVIFATR.V**

**MS/MS Fragmentation of R.EVSPTAMAIFR.K**

**MS/MS Fragmentation of R.EVSPTAMAIFRK.L**

**MS/MS Fragmentation of R.SREVSPTAMAIFR.K**

**Spot No.5 gi|122692295 ubiquitin carboxyl-terminal esterase L1 [Gallus gallus]**

| **Observed Mr** | **Mr(expt)** | **Mr(calc)** | **ppm** | **Start Seq.** | **End Seq.** | **Miss** | **Ion Score** | **Peptide sequence** |
| --- | --- | --- | --- | --- | --- | --- | --- | --- |
| 749.4080 | 748.4007 | 748.3980 | 3.64 | 132 | 137 | 1 |  | K.RFANNK.A |
| 871.4800 | 870.4727 | 870.4712 | 1.75 | 20 | 27 | 0 |  | R.LGVSPGWR.F |
| 937.4857 | 936.4784 | 936.4665 | 12.70 | 204 | 210 | 1 |  | R.QFTEREK.G |
| 1052.5426 | 1051.5353 | 1051.5233 | 11.40 | 201 | 208 | 1 |  | K.ICRQFTER.E |
| **1521.7342** | **1520.7269** | **1520.6994** | **18.10** | **117** | **129** | **0** | **91** | **K.FLDETADLSPEER.A** |
| 1521.7342 | 1520.7270 | 1520.6994 | 18.10 | 117 | 129 | 0 |  | K.FLDETADLSPEER.A |
| 1649.7910 | 1648.7837 | 1648.7944 | -6.48 | 116 | 129 | 1 |  | K.KFLDETADLSPEER.A |
| 1768.8240 | 1767.8167 | 1767.8322 | -8.76 | 138 | 153 | 0 |  | K.AIQEVHNSVAQEGQCR.V |
| 1852.9768 | 1851.9695 | 1851.9731 | -1.94 | 180 | 196 | 0 |  | R.LPFPVNHGTSSDDLLLK.D |

**MS/MS Fragmentation of K.FLDETADLSPEER.A**

**Spot No.10 gi|52138673 chaperonin containing TCP1, subunit 8 (theta) [Gallus gallus]**

| **Observed Mr** | **Mr(expt)** | **Mr(calc)** | **ppm** | **Start Seq.** | **End Seq.** | **Miss** | **Ion Score** | **Peptide sequence** |
| --- | --- | --- | --- | --- | --- | --- | --- | --- |
| 745.4211 | 744.4139 | 744.4031 | 14.50 | 319 | 323 | 1 |  | K.WDLRR.L |
| 818.4571 | 817.4499 | 817.4294 | 25.10 | 38 | 44 | 0 |  | K.ELAQTTR.T |
| 885.5278 | 884.5205 | 884.5080 | 14.20 | 327 | 335 | 0 |  | K.TVGATALPR.L |
| 908.5139 | 907.5066 | 907.4949 | 12.90 | 308 | 314 | 0 |  | K.YNLMIVR.L |
| 962.5384 | 961.5312 | 961.5055 | 26.70 | 8 | 16 | 0 |  | K.APGFAQMLK.E |
| 1064.5852 | 1063.5779 | 1063.5033 | 70.10 | 226 | 235 | 0 |  | K.ETEGDVTSVK.D |
| 1128.6754 | 1127.6682 | 1127.6662 | 1.69 | 510 | 520 | 0 |  | K.LATNAAVTVLR.V |
| 1150.5876 | 1149.5804 | 1149.5818 | -1.28 | 441 | 450 | 0 |  | K.FAEAFEAIPR.A |
| **1150.6900** | **1149.6827** | **1149.5818** | **87.80** | **441** | **450** | **0** | **70** | **K.FAEAFEAIPR.A** |
| 1158.6126 | 1157.6054 | 1157.5829 | 19.40 | 467 | 476 | 0 |  | K.LYAVHQEGNK.N |
| 1172.5833 | 1171.5760 | 1171.5509 | 21.40 | 172 | 181 | 0 |  | K.QYGNESFLSK.L |
| 1187.6685 | 1186.6613 | 1186.6557 | 4.66 | 368 | 378 | 0 |  | K.EDGAISTILIR.G |
| 1278.6850 | 1277.6777 | 1277.6768 | 0.75 | 440 | 450 | 1 |  | K.KFAEAFEAIPR.A |
| 1323.6284 | 1322.6211 | 1322.6255 | -3.31 | 21 | 31 | 0 |  | K.HYSGLEEAVYR.N |
| **1323.7450** | **1322.7377** | **1322.6255** | **84.90** | **21** | **31** | **0** | **54** | **K.HYSGLEEAVYR.N** |
| 1333.7403 | 1332.7331 | 1332.7401 | -5.31 | 63 | 74 | 0 |  | K.LFVTNDAATILR.E |
| **1333.8748** | **1332.8675** | **1332.7401** | **95.60** | **63** | **74** | **0** | **43** | **K.LFVTNDAATILR.E** |
| 1365.6171 | 1364.6098 | 1364.5878 | 16.10 | 379 | 390 | 0 |  | R.GSTDNLMDDIER.A |
| 1538.7596 | 1537.7523 | 1537.7698 | -11.36 | 491 | 504 | 0 |  | K.DMLEAGILDTYLGK.Y |
| 1540.7862 | 1539.7789 | 1539.7490 | 19.40 | 121 | 134 | 0 |  | R.MGLSVSEVIEGYEK.A |
| 1581.8573 | 1580.8501 | 1580.8522 | -1.34 | 365 | 378 | 1 |  | K.HEKEDGAISTILIR.G |
| 2114.0798 | 2113.0725 | 2113.0837 | -5.31 | 153 | 171 | 1 |  | K.NLRDVDEVASLLHTSVMSK.Q |
| 2439.2260 | 2438.2187 | 2438.1457 | 30.00 | 261 | 281 | 1 |  | K.NAEELMNFSKGEENLMDLQVK.A |

**MS/MS Fragmentation of K.FAEAFEAIPR.A**

**MS/MS Fragmentation of K.HYSGLEEAVYR.N**

**MS/MS Fragmentation of K.LFVTNDAATILR.E**

**Spot No. 11 gi|52138673 chaperonin containing TCP1, subunit 8 (theta) [Gallus gallus]**

| **Observed Mr** | **Mr(expt)** | **Mr(calc)** | **ppm** | **Start Seq.** | **End Seq.** | **Miss** | **Ion Score** | **Peptide sequence** |
| --- | --- | --- | --- | --- | --- | --- | --- | --- |
| 745.4194 | 744.4121 | 744.4031 | 12.10 | 319 | 323 | 1 |  | K.WDLRR.L |
| 818.4455 | 817.4383 | 817.4294 | 10.90 | 38 | 44 | 0 |  | K.ELAQTTR.T |
| 885.5177 | 884.5105 | 884.5080 | 2.81 | 327 | 335 | 0 |  | K.TVGATALPR.L |
| 908.5118 | 907.5045 | 907.4949 | 10.50 | 308 | 314 | 0 |  | K.YNLMIVR.L |
| 962.5232 | 961.5159 | 961.5055 | 10.80 | 8 | 16 | 0 |  | K.APGFAQMLK.E |
| 1031.5679 | 1030.5607 | 1030.5560 | 4.56 | 315 | 322 | 1 |  | R.LNSKWDLR.R |
| 1121.6094 | 1120.6021 | 1120.5876 | 12.90 | 75 | 84 | 0 |  | R.ELEVQHPAAK.M |
| 1128.6638 | 1127.6565 | 1127.6662 | -8.61 | 510 | 520 | 0 |  | K.LATNAAVTVLR.V |
| 1150.5734 | 1149.5661 | 1149.5818 | -13.70 | 441 | 450 | 0 |  | K.FAEAFEAIPR.A |
| **1150.5734** | **1149.5661** | **1149.5818** | **-13.66** | **441** | **450** | **0** | **80** | **K.FAEAFEAIPR.A** |
| 1158.5999 | 1157.5926 | 1157.5829 | 8.42 | 467 | 476 | 0 |  | K.LYAVHQEGNK.N |
| 1172.5709 | 1171.5636 | 1171.5509 | 10.80 | 172 | 181 | 0 |  | K.QYGNESFLSK.L |
| 1187.6533 | 1186.6460 | 1186.6557 | -8.22 | 368 | 378 | 0 |  | K.EDGAISTILIR.G |
| 1278.6686 | 1277.6613 | 1277.6768 | -12.09 | 440 | 450 | 1 |  | K.KFAEAFEAIPR.A |
| 1323.6128 | 1322.6055 | 1322.6255 | -15.09 | 21 | 31 | 0 |  | K.HYSGLEEAVYR.N |
| 1333.7295 | 1332.7222 | 1332.7401 | -13.46 | 63 | 74 | 0 |  | K.LFVTNDAATILR.E |
| **1333.7295** | **1332.7222** | **1332.7401** | **-13.45** | **63** | **74** | **0** | **52** | **K.LFVTNDAATILR.E** |
| 1354.7667 | 1353.7594 | 1353.7504 | 6.71 | 408 | 421 | 0 |  | R.LVPGGGATEIELAK.Q |
| 1365.602 | 1364.5947 | 1364.5878 | 5.04 | 379 | 390 | 0 |  | R.GSTDNLMDDIER.A |
| 1510.8416 | 1509.8343 | 1509.8515 | -11.35 | 407 | 421 | 1 |  | K.RLVPGGGATEIELAK.Q |
| 1540.7704 | 1539.7632 | 1539.7490 | 9.17 | 121 | 134 | 0 |  | R.MGLSVSEVIEGYEK.A |
| 1581.8382 | 1580.8309 | 1580.8522 | -13.44 | 365 | 378 | 1 |  | K.HEKEDGAISTILIR.G |
| 1708.8255 | 1707.8182 | 1707.8216 | -1.99 | 17 | 31 | 1 |  | K.EGAKHYSGLEEAVYR.N |
| 2114.0490 | 2113.0417 | 2113.0837 | -19.90 | 153 | 171 | 1 |  | K.NLRDVDEVASLLHTSVMSK.Q |
| 2122.0456 | 2121.0383 | 2121.0863 | -22.62 | 297 | 314 | 1 |  | K.VADMALHYANKYNLMIVR.L |
| 2298.2182 | 2297.2110 | 2297.2565 | -19.84 | 55 | 74 | 1 |  | K.MVINHLEKLFVTNDAATILR.E |
| 2412.0860 | 2411.0787 | 2411.0911 | -5.15 | 379 | 400 | 1 |  | R.GSTDNLMDDIERAVDDGVNTFK.V |
| 2439.1731 | 2438.1658 | 2438.1457 | 8.24 | 261 | 281 | 1 |  | K.NAEELMNFSKGEENLMDLQVK.A |
| 3000.6562 | 2999.6489 | 2999.6954 | -15.49 | 510 | 539 | 1 |  | K.LATNAAVTVLRVDQIIMAKPAGGPKPPSGK.K |

**MS/MS Fragmentation of K.FAEAFEAIPR.A**

**MS/MS Fragmentation of K.LFVTNDAATILR.E**

**Spot No. 12 gi|124249432 Rho GDP dissociation inhibitor (GDI) alpha [Gallus gallus]**

| **Observed Mr** | **Mr(expt)** | **Mr(calc)** | **ppm** | **Start Seq.** | **End Seq.** | **Miss** | **Ion Score** | **Peptide sequence** |
| --- | --- | --- | --- | --- | --- | --- | --- | --- |
| 752.3620 | 751.3547 | 751.3501 | 6.18 | 106 | 111 | 0 |  | K.EGVEYR.I |
| 763.4865 | 762.4792 | 762.4752 | 5.23 | 112 | 117 | 1 |  | R.IKISFR.V |
| **964.5007** | **963.4934** | **963.4926** | **0.81** | **128** | **134** | **0** | **54** | **K.YIQHTFR.K** |
| 964.5007 | 963.4934 | 963.4926 | 0.83 | 128 | 134 | 0 |  | K.YIQHTFR.K |
| 1092.5929 | 1091.5856 | 1091.5876 | -1.84 | 128 | 135 | 1 |  | K.YIQHTFRK.G |
| 1114.6346 | 1113.6273 | 1113.6506 | -20.89 | 118 | 127 | 1 |  | R.VNREIVSGLK.Y |
| 1259.5649 | 1258.5576 | 1258.5652 | -6.04 | 142 | 152 | 0 |  | K.TEYMVGSYGPR.A |
| 1438.7608 | 1437.7535 | 1437.7616 | -5.64 | 100 | 111 | 1 |  | K.QAFVLKEGVEYR.I |
| 1599.7534 | 1598.7461 | 1598.8127 | -41.61 | 139 | 152 | 1 |  | K.IVKTEYMVGSYGPR.A |
| 1783.8179 | 1782.8106 | 1782.8022 | 4.75 | 153 | 167 | 0 |  | R.AEEYEFLTPMEEAPK.G |
| 1917.9402 | 1916.9329 | 1916.9327 | 0.11 | 34 | 49 | 1 |  | K.SIQEIQELDKDDESLR.K |
| **1917.9402** | **1916.9329** | **1916.9327** | **0.13** | **34** | **49** | **1** | **97** | **K.SIQEIQELDKDDESLR.K** |
| 2179.2011 | 2178.1939 | 2178.1896 | 1.95 | 53 | 74 | 0 |  | K.EALLGAVTVTADPNAPNVVVTK.L |
| 2364.1071 | 2363.0998 | 2363.1070 | -3.04 | 181 | 199 | 1 |  | K.FTDDDKTDHLSWEWNLTIK.K |
| 2470.3398 | 2469.3325 | 2469.3479 | -6.24 | 51 | 74 | 1 |  | K.YKEALLGAVTVTADPNAPNVVVTK.L |

**MS/MS Fragmentation of K.YIQHTFR.K**

**MS/MS Fragmentation of K.SIQEIQELDKDDESLR.K**

**Spot No. 14 gi|71895337 ovoinhibitor precursor [Gallus gallus]**

| **Observed Mr** | **Mr(expt)** | **Mr(calc)** | **ppm** | **Start Seq.** | **End Seq.** | **Miss** | **Ion Score** | **Peptide sequence** |
| --- | --- | --- | --- | --- | --- | --- | --- | --- |
| 816.4412 | 815.4339 | 815.4324 | 1.87 | 172 | 178 | 0 |  | R.TLVACPR.I |
| **816.5010** | **815.4938** | **815.4324** | **75.30** | **172** | **178** | **0** | **28** | **R.TLVACPR.I** |
| 883.5038 | 882.4966 | 882.4195 | 87.30 | 72 | 79 | 0 |  | R.EHGANVEK.E |
| 913.5793 | 912.5720 | 912.5327 | 43.10 | 238 | 244 | 1 |  | K.LLVRCPR.I |
| 1120.4853 | 1119.4780 | 1119.4801 | -1.90 | 103 | 112 | 0 |  | R.DGNTMVACPR.I |
| 1136.4864 | 1135.4791 | 1135.4750 | 3.57 | 103 | 112 | 0 |  | R.DGNTMVACPR.I + Oxidation (M) |
| 1148.5147 | 1147.5074 | 1147.5081 | -0.57 | 38 | 47 | 0 |  | K.DGTSWVACPR.N |
| **1148.5917** | **1147.5845** | **1147.5081** | **66.60** | **38** | **47** | **0** | **46** | **K.DGTSWVACPR.N** |
| 1153.4976 | 1152.4903 | 1152.4870 | 2.89 | 80 | 88 | 0 |  | K.EYDGECRPK.H |
| 1716.8483 | 1715.8411 | 1715.8487 | -4.47 | 89 | 102 | 0 |  | K.HVMIDCSPYLQVVR.D |
| **1717.0044** | **1715.9972** | **1715.8487** | **86.50** | **89** | **102** | **0** | **82** | **K.HVMIDCSPYLQVVR.D** |
| 1732.8516 | 1731.8443 | 1731.8437 | 0.38 | 89 | 102 | 0 |  | K.HVMIDCSPYLQVVR.D + Oxidation (M) |
| 1763.7850 | 1762.7778 | 1762.7832 | -3.09 | 218 | 231 | 0 |  | R.QEIPEIDCDQYPTR.K |
| 1869.9492 | 1868.9419 | 1868.9190 | 12.30 | 152 | 168 | 1 |  | K.LEIGSVDCSKYPSTVSK.D |
| 1891.8790 | 1890.8717 | 1890.8782 | -3.41 | 218 | 232 | 1 |  | R.QEIPEIDCDQYPTRK.T |
| 1946.8955 | 1945.8882 | 1945.8874 | 0.44 | 145 | 161 | 1 |  | K.LHDGECKLEIGSVDCSK.Y |
| 2017.8924 | 2016.8852 | 2016.8959 | -5.33 | 72 | 88 | 1 |  | R.EHGANVEKEYDGECRPK.H |
| 2079.9141 | 2078.9068 | 2078.9150 | -3.94 | 216 | 231 | 1 |  | K.CRQEIPEIDCDQYPTR.K |
| 2778.1946 | 2777.1873 | 2777.2207 | -12.04 | 48 | 71 | 0 |  | R.NLKPVCGTDGSTYSNECGICLYNR.E |
| 3084.2787 | 3083.2714 | 3083.3172 | -14.85 | 179 | 205 | 0 |  | R.ILSPVCGTDGFTYDNECGICAHNAEQR.T |
| 3476.5107 | 3475.5034 | 3475.5595 | -16.16 | 245 | 275 | 0 |  | R.ILLPVCGTDGFTYDNECGICAHNAQHGTEVK.K |
| 3604.5916 | 3603.5843 | 3603.6545 | -19.48 | 245 | 276 | 1 |  | R.ILLPVCGTDGFTYDNECGICAHNAQHGTEVKK.S |
| 3642.5591 | 3641.5518 | 3641.6297 | -21.38 | 48 | 79 | 1 |  | R.NLKPVCGTDGSTYSNECGICLYNREHGANVEK.E |

**MS/MS Fragmentation of R.TLVACPR.I**

**MS/MS Fragmentation of K.DGTSWVACPR.N**

**MS/MS Fragmentation of K.HVMIDCSPYLQVVR.D**

**Spot No. 15 gi|45384222 Heat shock 27 kDa protein[Gallus gallus]**

| **Observed Mr** | **Mr(expt)** | **Mr(calc)** | **ppm** | **Start Seq.** | **End Seq.** | **Miss** | **Ion Score** | **Peptide sequence** |
| --- | --- | --- | --- | --- | --- | --- | --- | --- |
| 1088.5184 | 1087.5111 | 1087.5047 | 5.96 | 126 | 134 | 0 |  | K.QDEHGFISR.C |
| 1373.7232 | 1372.7159 | 1372.7351 | -13.94 | 140 | 152 | 0 |  | K.YTLPPGVEATAVR.S |
| **1373.7954** | **1372.7881** | **1372.7351** | **38.60** | **140** | **152** | **0** | **76** | **K.YTLPPGVEATAVR.S** |
| 1410.6332 | 1409.6259 | 1409.6517 | -18.25 | 45 | 56 | 0 |  | K.WPSGSAWPGYFR.L |
| 1501.8447 | 1500.8374 | 1500.8300 | 4.93 | 139 | 152 | 1 |  | R.KYTLPPGVEATAVR.S |
| 1611.7528 | 1610.7455 | 1610.7437 | 1.13 | 122 | 134 | 1 |  | K.HEEKQDEHGFISR.C |
| 1809.9695 | 1808.9622 | 1808.9673 | -2.77 | 95 | 110 | 0 |  | K.VTLDVNHFAPEELVVK.T |
| 1825.0011 | 1823.9938 | 1823.9781 | 8.59 | 57 | 74 | 0 | 2434.3  2266.2 | R.LLPSESALLPAPGSPYGR.A |
| 2109.9841 | 2108.9769 | 2108.9666 | 4.86 | 28 | 44 | 0 |  | R.LFDQSFGMPHIPEDWYK.W |
| 2612.2815 | 2611.2742 | 2611.3282 | -20.69 | 88 | 110 | 1 |  | R.QSADSWKVTLDVNHFAPEELVVK.T |
| 2712.2546 | 2711.2473 | 2711.2921 | -16.54 | 6 | 27 | 1 |  | R.VPFTFLTSPSWEPFRDWYHGSR.L |

**MS/MS Fragmentation of K.YTLPPGVEATAVR.S**

**Spot No.16 gi|50403707 myosin light chain type 2 (LC2f) [Gallus gallus]**

| **Observed Mr** | **Mr(expt)** | **Mr(calc)** | **ppm** | **Start Seq.** | **End Seq.** | **Miss** | **Ion Score** | **Peptide sequence** |
| --- | --- | --- | --- | --- | --- | --- | --- | --- |
| 882.4202 | 881.4130 | 881.4065 | 7.29 | 51 | 58 | 0 |  | R.ETFAAMGR.L |
| 898.4251 | 897.4178 | 897.4014 | 18.30 | 51 | 58 | 0 |  | R.ETFAAMGR.L + Oxidation (M) |
| 1159.6067 | 1158.5995 | 1158.5881 | 9.84 | 41 | 50 | 1 |  | R.DGIIDKDDLR.E |
| 1192.5918 | 1191.5845 | 1191.5884 | -3.23 | 31 | 40 | 0 |  | K.EAFTVIDQNR.D |
| 1448.7054 | 1447.6981 | 1447.6766 | 14.90 | 154 | 165 | 0 |  | K.NICYVITHGEDK.E |
| 1611.7584 | 1610.7511 | 1610.7610 | -6.16 | 117 | 129 | 0 |  | K.SFLEELLTTQCDR.F |
| **1611.8608** | **1610.8536** | **1610.7610** | **57.40** | **117** | **129** | **0** | **55** | **K.SFLEELLTTQCDR.F** |
| 1739.8602 | 1738.8529 | 1738.8560 | -1.76 | 116 | 129 | 1 |  | K.KSFLEELLTTQCDR.F |
| 1763.7953 | 1762.7880 | 1762.7832 | 2.71 | 154 | 168 | 1 |  | K.NICYVITHGEDKEGE.- |
| **1763.9026** | **1762.8953** | **1762.7832** | **63.60** | **154** | **168** | **1** | **98** | **K.NICYVITHGEDKEGE.-** |

**MS/MS Fragmentation of K.SFLEELLTTQCDR.F**

**MS/MS Fragmentation of K.NICYVITHGEDKEGE.-**

**Spot No.17 gi|45382875 M-CK [Gallus gallus]Creatine kinase M chain**

| **Observed Mr** | **Mr(expt)** | **Mr(calc)** | **ppm** | **Start Seq.** | **End Seq.** | **Miss** | **Ion Score** | **Peptide sequence** |
| --- | --- | --- | --- | --- | --- | --- | --- | --- |
| 759.3495 | 758.3422 | 758.3347 | 9.83 | 210 | 215 | 0 |  | R.DWPDAR.G |
| 879.4812 | 878.4739 | 878.4241 | 56.70 | 359 | 365 | 0 |  | K.LMVEMEK.K |
| 896.4989 | 895.4916 | 895.4916 | 0.03 | 171 | 177 | 1 |  | K.GRYYPLK.A |
| 934.5906 | 933.5833 | 933.5760 | 7.88 | 293 | 301 | 1 |  | R.GGVHVKLPK.L |
| 943.4984 | 942.4911 | 942.4923 | -1.26 | 308 | 314 | 0 |  | K.FEEILHR.L |
| 1037.5299 | 1036.5227 | 1036.5124 | 9.91 | 243 | 251 | 1 |  | K.GGNMKEVFR.R |
| 1118.6549 | 1117.6476 | 1117.6495 | -1.73 | 33 | 41 | 1 |  | K.VLTPELYKR.L |
| **1118.6871** | **1117.6799** | **1117.6495** | **27.10** | **33** | **41** | **1** | **67** | **K.VLTPELYKR.L** |
| 1182.6022 | 1181.5949 | 1181.5941 | 0.65 | 2 | 11 | 1 |  | M.PFSSTHNKHK.L |
| 1193.6137 | 1192.6064 | 1192.5944 | 10.10 | 237 | 247 | 1 |  | R.VISMEKGGNMK.E |
| 1212.6782 | 1211.6710 | 1211.6775 | -5.37 | 308 | 316 | 1 |  | K.FEEILHRLR.L |
| 1217.6065 | 1216.5992 | 1216.6088 | -7.92 | 87 | 96 | 0 |  | K.DLFDPVIQDR.H |
| 1295.6535 | 1294.6462 | 1294.6531 | -5.27 | 97 | 107 | 1 |  | R.HGGYKPTDKHR.T |
| 1521.7486 | 1520.7413 | 1520.7471 | -3.80 | 117 | 130 | 1 |  | K.GGDDLDPKYVLSSR.V |
| 1633.8820 | 1632.8748 | 1632.8736 | 0.71 | 302 | 314 | 1 |  | K.LSQHPKFEEILHR.L |
| **1633.9435** | **1632.9363** | **1632.8736** | **38.40** | **302** | **314** | **1** | **72** | **K.LSQHPKFEEILHR.L** |
| 1639.8043 | 1638.7971 | 1638.8141 | -10.38 | 12 | 25 | 1 |  | K.LKFSAEEEFPDLSK.H |
| 1748.9046 | 1747.8973 | 1747.9104 | -7.52 | 157 | 172 | 1 |  | K.LSVEALNSLEGEFKGR.Y |
| 1992.9501 | 1991.9429 | 1991.9549 | -6.03 | 321 | 341 | 0 |  | R.GTGGVDTAAVGAVFDISNADR.L |
| 2149.0718 | 2148.0645 | 2148.0560 | 3.96 | 320 | 341 | 1 |  | K.RGTGGVDTAAVGAVFDISNADR.L |
| 2231.0109 | 2230.0036 | 2230.0113 | -3.45 | 14 | 32 | 1 |  | K.FSAEEEFPDLSKHNNHMAK.V |
| 2622.2678 | 2621.2606 | 2621.2775 | -6.47 | 216 | 236 | 1 |  | R.GIWHNDNKTFLVWVNEEDHLR.V |

**MS/MS Fragmentation of K.VLTPELYKR.L**

**MS/MS Fragmentation of K.LSQHPKFEEILHR.L**
