# Supplementary material for: Proteomic analysis of chicken embryonic trachea and kidney tissues after infection in ovo by avian infectious bronchitis coronavirus
Source: Proteome Sci. 2011 Mar 8;9:11. doi: 10.1186/1477-5956-9-11 (PMC3060854; doi:10.1186/1477-5956-9-11)
Supplement: Additional file 2 — Additional_file_2.doc containing the PMF spectrum and Mascot database search results of differentially expressed protein spots in IBV-infected chicken embryo tracheal tissues. [file 1477-5956-9-11-S2.DOC]

**Additional file 2**

This includes PMF spectrum and Mascot database search results for 8 differentially expressed protein spots in IBV-infected chicken embryo tracheal tissues.

**Spot No. 1**

**A. PMF spectrum**

**B. PMF database search result**

Match to: **gi|55584150** Score: **81** Expect: **0.074**

**RecName: Full=Myosin light chain 3, skeletal muscle isoform; AltName: Full=A2 catalytic; AltName: Full=Alkali myosin light chain 3; Short=MLC-3; AltName: Full=Myosin light chain 3f; AltName: Full=Skeletal-muscle myosin L-4 light chain**

Number of mass values searched: **29**

Number of mass values matched: **6**

Sequence Coverage: **38%**

Matched peptides shown in **Bold Red**

**1** MSFSPDEIND FKEAFLLFDR **TGDAKITLSQ VGDIVRALGQ NPTNAEINK**I

**51** LGNPSKEEMN AKKITFEEFL PMLQAAANNK **DQGTFEDFVE GLRVFDKEGN**

**101 GTVMGAELR**H VLATLGEKMT EEEVEELMKG QEDSNGCINY EAFVKHIMSV

**151**

Matched peptides sorted by Residue Number

Start - End Observed Mr(expt) Mr(calc) ppm Miss Sequence

21 - 36 1672.9092 1671.9019 1671.9156 -8 1 R.TGDAKITLSQVGDIVR.A

26 - 36 1200.6902 1199.6829 1199.6874 -4 0 K.ITLSQVGDIVR.A

37 - 49 1369.7013 1368.6940 1368.6997 -4 0 R.ALGQNPTNAEINK.I

81 - 93 1512.6850 1511.6777 1511.6893 -8 0 K.DQGTFEDFVEGLR.V

94 - 109 1722.8303 1721.8231 1721.8407 -10 1 R.VFDKEGNGTVMGAELR.H

98 - 109 1233.5721 1232.5648 1232.5819 -14 0 K.EGNGTVMGAELR.H

**Spot No. 4**

**A. PMF spectrum**

**B. PMF database search result**

Match to: **gi|150247116** Score: **289** Expect: **1.3e-22**

**hypothetical protein LOC430359 [Gallus gallus]**

Number of mass values searched: **25**

Number of mass values matched: **20**

Sequence Coverage: **46%**

Matched peptides shown in **Bold Red**

**1** MAASNPTPLL PSEASCPICL EYFRDPVSIH CGHNFCRQCI TRCWEWSTGG

**51** FCCPQCKETA EERVLCPNRE LAR**VLEIARR** LSLQAAHR**DA AGQEGCEK**HR

**101** **EPLSIYCKDD EAFICVICR**E SRLHR**AHAML PVQDAVQEYK EQIQSHLQAL**

**151 K**EDRDKLLGF REAEMRR**NWE YLEK**TSAERQ K**ILGGFEGLR LFLEEQEHHL**

**201 LAQLENMER**D VEK**TQEENVT ILTKEISHLD TIIQEMEEK**C QQPASKFLQD

**251** IRSTLSRLGK ENFQQPTLLL PDLESNLSHF REK**NNALEEI LK**NFK**EILMF**

**301 ELPEKMSVTL DPSTAHPQLT VSEDGR**SVR**W EDTQRDAASE EFGTDPFVLG**

**351 HEGITSGRCC WEVEVTPKGS WAVGVAK**ESL KRREESGMSS EIELWSMGFC

**401** EGQFWALSSF ER**MTLPQIQV PRR**VRVTLDY ERGQVAFFDA DKRALIFIFP

**451** SASFKGESIH PWFLVWGEGS QITLCP

Matched peptides sorted by Residue Number

Start - End Observed Mr(expt) Mr(calc) ppm Miss Sequence

74 - 80 856.5249 855.5177 855.5290 -13 1 R.VLEIARR.L

89 - 98 1007.4350 1006.4277 1006.4026 25 0 R.DAAGQEGCEK.H

101 - 108 952.4450 951.4377 951.4735 -38 0 R.EPLSIYCK.D

109 - 119 1283.5400 1282.5327 1282.5686 -28 0 K.DDEAFICVICR.E

126 - 140 1699.8110 1698.8037 1698.8399 -21 0 R.AHAMLPVQDAVQEYK.E

141 - 151 1294.6750 1293.6677 1293.7041 -28 0 K.EQIQSHLQALK.E

168 - 174 981.5090 980.5017 980.4603 42 0 R.NWEYLEK.T

182 - 190 961.5880 960.5807 960.5393 43 0 K.ILGGFEGLR.L

191 - 209 2379.2170 2378.2097 2378.1688 17 0 R.LFLEEQEHHLLAQLENMER.D

214 - 224 1275.7200 1274.7127 1274.6718 32 0 K.TQEENVTILTK.E

225 - 239 1814.9250 1813.9177 1813.8767 23 0 K.EISHLDTIIQEMEEK.C

284 - 292 1043.5540 1042.5467 1042.5658 -18 0 K.NNALEEILK.N

296 - 305 1248.6350 1247.6277 1247.6471 -16 0 K.EILMFELPEK.M

306 - 326 2241.0630 2240.0557 2240.0743 -8 0 K.MSVTLDPSTAHPQLTVSEDGR.S

330 - 335 834.3550 833.3477 833.3668 -23 0 R.WEDTQR.D

336 - 358 2392.0860 2391.0787 2391.0979 -8 0 R.DAASEEFGTDPFVLGHEGITSGR.C

359 - 368 1193.5790 1192.5717 1192.5257 39 0 R.CCWEVEVTPK.G

369 - 377 874.5240 873.5167 873.4709 53 0 K.GSWAVGVAK.E

413 - 422 1182.7120 1181.7047 1181.6591 39 0 R.MTLPQIQVPR.R

413 - 423 1338.8240 1337.8167 1337.7602 42 1 R.MTLPQIQVPRR.V

**Spot No. 6**

**A. PMF spectrum**

**B. PMF database search result**

Match to: **gi|45382983** Score: **87** Expect: **0.019**

**replication factor C (activator 1) 2, 40kDa [Gallus gallus]**

Number of mass values searched: **95**

Number of mass values matched: **14**

Sequence Coverage: **45%**

Matched peptides shown in **Bold Red**

**1** MEEEEVLEVV EDEK**AGPAAA EKRGPTDTLG SAPAASGHYE LPWVEK**YRPL

**51** K**LCEVVGNED TVSRLEVFAK** EGNVPNIIIA GPPGTGK**TTS ILCLARALLG**

**101 PALKDAVLEL NASNDR**GIDV VRNKIKMFAQ QKVTLPKGRH K**IIILDEADS**

**151 MTDGAQQALR R**TMEIYSKTT R**FALACNASD KIIEPIQSR**C AVLRYTK**LTD**

**201 SQILAR**LLKI VEKEDVPYTD DGLEAIIFTA QGDMRQALNN LQSTYSGFGF

**251** INSENVFKVC DEPHPLLVKE MIQHCINANI DEAYK**ILAHL WRLGYSPEDV**

**301 IGNIFR**VCKT FQMPEYLKLE FIKEIGYTHM K**IAEGVNSLL QMAGLLAR**LC

**351** QKTAAPAAS

Matched peptides sorted by Residue Number

Start - End Observed Mr(expt) Mr(calc) ppm Miss Sequence

15 - 23 870.5415 869.5342 869.4719 72 1 K.AGPAAAEKR.G

23 - 46 2539.2649 2538.2576 2538.2503 3 1 K.RGPTDTLGSAPAASGHYELPWVEK.Y

52 - 64 1477.7243 1476.7170 1476.6879 20 0 K.LCEVVGNEDTVSR.L

52 - 70 2165.0812 2164.0739 2164.0834 -4 1 K.LCEVVGNEDTVSRLEVFAK.E

88 - 96 1034.5687 1033.5614 1033.5590 2 0 K.TTSILCLAR.A

97 - 116 2080.1342 2079.1269 2079.1324 -3 1 R.ALLGPALKDAVLELNASNDR.G

105 - 116 1316.7637 1315.7565 1315.6368 91 0 K.DAVLELNASNDR.G

142 - 161 2216.1181 2215.1108 2215.1266 -7 1 K.IIILDEADSMTDGAQQALRR.T

172 - 189 2033.0468 2032.0395 2032.0411 -1 1 R.FALACNASDKIIEPIQSR.C

182 - 189 955.5557 954.5485 954.5498 -1 0 K.IIEPIQSR.C

198 - 206 1016.5646 1015.5574 1015.5662 -9 0 K.LTDSQILAR.L

286 - 292 908.5502 907.5430 907.5392 4 0 K.ILAHLWR.L

293 - 306 1579.8114 1578.8041 1578.8042 -0 0 R.LGYSPEDVIGNIFR.V

332 - 348 1755.9535 1754.9462 1754.9713 -14 0 K.IAEGVNSLLQMAGLLAR.L

**Spot No. 7**

**A. PMF spectrum**

**B. PMF database search result**

Match to: **gi|71896049** Score: **142** Expect: **6.5e-08**

**cholinergic receptor, nicotinic, gamma polypeptide precursor [Gallus gallus]**

Number of mass values searched: **26**

Number of mass values matched: **13**

Sequence Coverage: **26%**

Matched peptides shown in **Bold Red**

**1** MRCSDLLLLF LLALCVLPGI SCRNQEEKLL QDLMTNYNR**H LRPALRGDQV**

**51 IDVTLKLTLT NLISLNEREE TLTTNVWIEM QWSDYR**LRWD PDK**YDDIQQL**

**101 R**VPSAMVWLP DIVLENNIDG TFEITLYTNV LVYPDGSIYW LPPAIYRSSC

**151** SIHVTYFPFD WQNCTMVFQS QTYSANEINL LLTVEEGQTI EWIFIDPEAF

**201** TENGEWAIKH RPARKIINSG RFTPDDIQYQ QVIFYLIIQR KPLFYIINII

**251** VPCVLISSMA VLVYFLPAKA GGQKCTVSIN VLLAQTVFLF LIAQK**VPETS**

**301 QAVPLIGK**YL TFLMVVTVVI VVNAVIVLNV SLR**TPNTHSM SQR**VR**QVWLH**

**351 LLPRYLGMHM PEEAPGPPQA TR**RR**SSLGLM VK**ADEYMLWK ARTELLFEKQ

**401** K**ERDGLMK**TV LEKIGRGLES NCAQDFCQSL EEASPEIRAC VEACNHIANA

**451** TR**EQNDFSSE NEEWILVGR**V IDRVCFFIMA SLFVCGTIGI FLMAHFNQAP

**501** ALPFPGDPKT YLPP

Matched peptides sorted by Residue Number

Start - End Observed Mr(expt) Mr(calc) ppm Miss Sequence

40 - 46 862.5620 861.5547 861.5297 29 0 R.HLRPALR.G

47 - 56 1087.6240 1086.6167 1086.5921 23 0 R.GDQVIDVTLK.L

57 - 68 1386.7590 1385.7517 1385.7878 -26 0 K.LTLTNLISLNER.E

69 - 86 2301.0130 2300.0057 2300.0419 -16 0 R.EETLTTNVWIEMQWSDYR.L

94 - 101 1050.4850 1049.4777 1049.5142 -35 0 K.YDDIQQLR.V

296 - 308 1338.8040 1337.7967 1337.7555 31 0 K.VPETSQAVPLIGK.Y

296 - 308 1338.8218 1337.8146 1337.7555 44 0 K.VPETSQAVPLIGK.Y

334 - 343 1158.5730 1157.5657 1157.5248 35 0 R.TPNTHSMSQR.V

346 - 354 1161.7300 1160.7227 1160.6818 35 0 R.QVWLHLLPR.Y

355 - 372 1981.9670 1980.9597 1980.9186 21 0 R.YLGMHMPEEAPGPPQATR.R

375 - 382 834.5160 833.5087 833.4681 49 0 R.SSLGLMVK.A

402 - 408 864.4922 863.4849 863.4171 79 1 K.ERDGLMK.T Oxidation (M)

453 - 469 2051.9110 2050.9037 2050.9232 -9 0 R.EQNDFSSENEEWILVGR.V

**Spot No. 8-1**

**A. PMF spectrum**

**B. PMF database search result**

Match to: **gi|45382569** Score: **157** Expect: **2e-09**

**ARP2 actin-related protein 2 homolog [Gallus gallus]**

Number of mass values searched: **92**

Number of mass values matched: **23**

Sequence Coverage: **51%**

Matched peptides shown in **Bold Red**

**1** MDTLGR**KVVV CDNGTGFVKC GYAGSNFPEH IFPALVGRPI IR**STAK**VGNI**

**51 EIKDLMVGDE ASELRSMLEV NYPMENGIVR NWDDMKHLWD YTFGPEK**LNI

**101** DTK**NCKILLT EPPMNPTK**NR EKIVEVMFET YQFSGVYVAI QAVLTLYAQG

**151** LLTGVVVDSG DGVTHICPVY EGFSLPHLTR **RLDIAGR**DIT RYLIKLLLLR

**201** **GYAFNHSADF ETVR**MIKEK**L CYVGYNIEQE QKLALETTVL VESYTLPDGR**

**251** IIKVGGERFE APEALFQPHL INVEGVGVAE LLFNTIQAAD IDTR**SEFYKH**

**301 IVLSGGSTMY PGLPSR**LER**E LKQLYLER**VL KGDVEKLSKF K**IRIEDPPR**R

**351** KHMVFLGGAV LADIMK**DKDN FWMTR**QEYQE KGVRVLEKLG VTVR

Matched peptides sorted by Residue Number

Start - End Observed Mr(expt) Mr(calc) ppm Miss Sequence

7 - 19 1422.7435 1421.7362 1421.7337 2 1 R.KVVVCDNGTGFVK.C

8 - 19 1294.6424 1293.6351 1293.6388 -3 0 K.VVVCDNGTGFVK.C

20 - 42 2571.3139 2570.3066 2570.3216 -6 0 K.CGYAGSNFPEHIFPALVGRPIIR.S

47 - 65 2088.0603 2087.0531 2087.0568 -2 1 K.VGNIEIKDLMVGDEASELR.S

47 - 65 2104.0642 2103.0569 2103.0518 2 1 K.VGNIEIKDLMVGDEASELR.S Oxidation (M)

54 - 65 1334.6319 1333.6246 1333.6184 5 0 K.DLMVGDEASELR.S

66 - 80 1751.8407 1750.8335 1750.8382 -3 0 R.SMLEVNYPMENGIVR.N

66 - 80 1767.8480 1766.8407 1766.8331 4 0 R.SMLEVNYPMENGIVR.N Oxidation (M)

81 - 97 2181.9800 2180.9727 2180.9626 5 1 R.NWDDMKHLWDYTFGPEK.L

87 - 97 1392.6688 1391.6615 1391.6510 8 0 K.HLWDYTFGPEK.L

104 - 118 1771.9098 1770.9025 1770.9008 1 1 K.NCKILLTEPPMNPTK.N Oxidation (M)

107 - 118 1353.7523 1352.7451 1352.7374 6 0 K.ILLTEPPMNPTK.N

181 - 187 800.4763 799.4691 799.4664 3 1 R.RLDIAGR.D

201 - 214 1613.7350 1612.7277 1612.7270 0 0 R.GYAFNHSADFETVR.M

220 - 232 1643.7823 1642.7750 1642.7661 5 0 K.LCYVGYNIEQEQK.L

233 - 250 1977.0541 1976.0468 1976.0466 0 0 K.LALETTVLVESYTLPDGR.I

295 - 316 2426.2218 2425.2145 2425.2100 2 1 R.SEFYKHIVLSGGSTMYPGLPSR.L

300 - 316 1787.9118 1786.9046 1786.9036 1 0 K.HIVLSGGSTMYPGLPSR.L Oxidation (M)

320 - 328 1191.6703 1190.6630 1190.6659 -2 1 R.ELKQLYLER.V

323 - 328 821.4542 820.4469 820.4443 3 0 K.QLYLER.V

342 - 349 995.5660 994.5588 994.5560 3 1 K.IRIEDPPR.R

367 - 375 1212.5498 1211.5426 1211.5393 3 1 K.DKDNFWMTR.Q

369 - 375 969.4283 968.4210 968.4174 4 0 K.DNFWMTR.Q

**Spot No. 8-2**

**A. PMF spectrum same as Spot No. 8-1**

**B. PMF database search result**

Match to: **gi|17942831** Score: **93** Expect: **0.0056**

**Chain A, Ovotransferrin, C-Terminal Lobe, Apo Form**

Number of mass values searched: **92**

Number of mass values matched: **14**

Sequence Coverage: **49%**

Matched peptides shown in **Bold Red**

**1** ENRIQWCAVG KDEKSKCDRW SVVSNGDVEC TVVDETK**DCI IKIMKGEADA**

**51 VALDGGLVYT AGVCGLVPVM AER**YDDESQC SK**TDERPASY FAVAVAR**KDS

**101** NVNWNNLKGK KSCHTAVGRT AGWVIPMGLI HNR**TGTCNFD EYFSEGCAPG**

**151 SPPNSRLCQL CQGSGGIPPE KCVASSHEKY FGYTGALR**CL VEKGDVAFIQ

**201** HSTVEENTGG KNKADWAK**NL QMDDFELLCT DGRR**ANVMDY R**ECNLAEVPT**

**251 HAVVVRPEK**A NK**IRDLLER**Q EKRFGVNGSE K**SKFMMFESQ NK**DLLFKDLT

**301** KCLFKVR**EGT TYKEFLGDK**F YTVISSLKTC NPSDILQMCS FLEGK

Matched peptides sorted by Residue Number

Start - End Observed Mr(expt) Mr(calc) ppm Miss Sequence

38 - 45 1036.5438 1035.5366 1035.5457 -9 1 K.DCIIKIMK.G Oxidation (M)

46 - 73 2790.3576 2789.3503 2789.3728 -8 0 K.GEADAVALDGGLVYTAGVCGLVPVMAER.Y

83 - 97 1652.8380 1651.8308 1651.8318 -1 0 K.TDERPASYFAVAVAR.K

134 - 156 2550.0198 2549.0125 2549.0224 -4 0 R.TGTCNFDEYFSEGCAPGSPPNSR.L

157 - 171 1643.7823 1642.7750 1642.7807 -3 0 R.LCQLCQGSGGIPPEK.C

172 - 188 1945.9229 1944.9156 1944.9152 0 1 K.CVASSHEKYFGYTGALR.C

180 - 188 1047.5279 1046.5207 1046.5185 2 0 K.YFGYTGALR.C

219 - 233 1826.8079 1825.8006 1825.7975 2 0 K.NLQMDDFELLCTDGR.R

219 - 234 1982.9110 1981.9037 1981.8986 3 1 K.NLQMDDFELLCTDGRR.A

219 - 234 1999.0345 1998.0272 1997.8935 67 1 K.NLQMDDFELLCTDGRR.A Oxidation (M)

242 - 259 2048.0529 2047.0457 2047.0520 -3 0 R.ECNLAEVPTHAVVVRPEK.A

263 - 269 914.5439 913.5366 913.5345 2 1 K.IRDLLER.Q

282 - 292 1392.6688 1391.6615 1391.6213 29 1 K.SKFMMFESQNK.D Oxidation (M)

308 - 319 1387.7503 1386.7430 1386.6667 55 1 R.EGTTYKEFLGDK.F

**Spot No. 9**

**A. PMF spectrum**

**B. PMF database search result**

Match to: **gi|45383758** Score: **159** Expect: **1.3e-09**

**cholinergic receptor, nicotinic, alpha 7 precursor [Gallus gallus]**

Number of mass values searched: **28**

Number of mass values matched: **14**

Sequence Coverage: **29%**

Matched peptides shown in **Bold Red**

**1** MGLR**ALMLWL LAAAGLVRES LQGEFQR**KLY KELLKNYNPL ERPVANDSQP

**51** LTVYFTLSLM QIMDVDEKNQ VLTTNIWLQM YWTDHYLQWN VSEYPGVKNV

**101** R**FPDGLIWKP DILLYNSADE R**FDATFHTNV LVNSSGHCQY LPPGIFK**SSC**

**151 YIDVRWFPFD VQK**CNLKFGS WTYGGWSLDL QMQEADISGY ISNGEWDLVG

**201** IPGKR**TESFY ECCKEPYPDI TFTVTMR**RRT LYYGLNLLIP CVLISALALL

**251** VFLLPADSGE KISLGITVLL SLTVFMLLVA EIMPATSDSV PLIAQYFAST

**301** MIIVGLSVVV TVIVLQYHHH DPDGGKMPKW TR**VILLNWCA WFLR**MKRPGE

**351** DK**VRPACQHK** QRRCSLSSME MNTVSGQQCS NGNMLYIGFR **GLDGVHCTPT**

**401 TDSGVICGR**M TCSPTEEENL LHSGHPSEGD PDLAKILEEV R**YIANRFRDQ**

**451 DEEEAICNEW KFAASVVDR**L CLMAFSVFTI ICTIGILMSA PNFVEAVSKD

**501** FA

Matched peptides sorted by Residue Number

Start - End Observed Mr(expt) Mr(calc) ppm Miss Sequence

5 - 18 1497.9220 1496.9147 1496.8901 16 0 R.ALMLWLLAAAGLVR.E

19 - 27 1093.5520 1092.5447 1092.5200 23 0 R.ESLQGEFQR.K

102 - 121 2362.2330 2361.2257 2361.2005 11 0 R.FPDGLIWKPDILLYNSADER.F

148 - 155 942.4600 941.4527 941.4277 27 0 K.SSCYIDVR.W

156 - 163 1066.5000 1065.4927 1065.5284 -33 0 R.WFPFDVQK.C

206 - 214 1109.3920 1108.3847 1108.4205 -32 0 R.TESFYECCK.E

215 - 227 1569.7260 1568.7187 1568.7545 -23 0 K.EPYPDITFTVTMR.R

333 - 344 1533.8040 1532.7967 1532.8326 -23 0 R.VILLNWCAWFLR.M

353 - 360 938.5400 937.5327 937.4916 44 0 K.VRPACQHK.Q

391 - 409 1887.9100 1886.9027 1886.8615 22 0 R.GLDGVHCTPTTDSGVICGR.M

442 - 448 939.5813 938.5740 938.5086 70 1 R.YIANRFR.D

449 - 461 1608.6890 1607.6817 1607.6409 25 0 R.DQDEEEAICNEWK.F

462 - 469 864.4951 863.4878 863.4501 44 0 K.FAASVVDR.L

462 - 469 864.4980 863.4907 863.4501 47 0 K.FAASVVDR.L

**Spot No. 13**

**A. PMF spectrum**

**B. PMF database search result**

Match to: **gi|71895337** Score: **128** Expect: **1.6e-06**

**ovoinhibitor precursor [Gallus gallus]**

Number of mass values searched: **93**

Number of mass values matched: **17**

Sequence Coverage: **39%**

Matched peptides shown in **Bold Red**

**1** MRTARQFVQV ALALCCFADI AFGIEVNCSL YASGIGK**DGT SWVACPRNLK**

**51 PVCGTDGSTY SNECGICLYN REHGANVEKE YDGECRPKHV MIDCSPYLQV**

**101 VRDGNTMVAC PR**ILKPVCGS DSFTYDNECG ICAYNAEHHT NISK**LHDGEC**

**151 KLEIGSVDCS KYPSTVSKDG RTLVACPRIL SPVCGTDGFT YDNECGICAH**

**201 NAEQR**THVSK KHDGK**CRQEI PEIDCDQYPT RK**TTGGKLLV RCPR**ILLPVC**

**251 GTDGFTYDNE CGICAHNAQH GTEVKK**SHDG RCKERSTPLD CTQYLSNTQN

**301** GEAITACPFI LQEVCGTDGV TYSNDCSLCA HNIELGTSVA KKHDGRCREE

**351** VPELDCSKYK TSTLKDGRQV VACTMIYDPV CATNGVTYAS ECTLCAHNLE

**401** QRTNLGKRKN GRCEEDITKE HCREFQKVSP ICTMEYVPHC GSDGVTYSNR

**451** CFFCNAYVQS NRTLNLVSMA AC

Matched peptides sorted by Residue Number

Start - End Observed Mr(expt) Mr(calc) ppm Miss Sequence

38 - 47 1148.5256 1147.5183 1147.5081 9 0 K.DGTSWVACPR.N

48 - 71 2778.1990 2777.1917 2777.2207 -10 0 R.NLKPVCGTDGSTYSNECGICLYNR.E

72 - 88 2017.8969 2016.8896 2016.8959 -3 1 R.EHGANVEKEYDGECRPK.H

80 - 88 1153.5056 1152.4984 1152.4870 10 0 K.EYDGECRPK.H

89 - 102 1716.8530 1715.8458 1715.8487 -2 0 K.HVMIDCSPYLQVVR.D

89 - 102 1732.8547 1731.8474 1731.8437 2 0 K.HVMIDCSPYLQVVR.D Oxidation (M)

103 - 112 1120.4952 1119.4879 1119.4801 7 0 R.DGNTMVACPR.I

103 - 112 1136.4934 1135.4862 1135.4750 10 0 R.DGNTMVACPR.I Oxidation (M)

145 - 161 1946.9161 1945.9088 1945.8874 11 1 K.LHDGECKLEIGSVDCSK.Y

152 - 168 1869.9410 1868.9337 1868.9190 8 1 K.LEIGSVDCSKYPSTVSK.D

162 - 171 1109.5744 1108.5671 1108.5513 14 1 K.YPSTVSKDGR.T

172 - 178 816.4415 815.4343 815.4324 2 0 R.TLVACPR.I

179 - 205 3084.2805 3083.2732 3083.3172 -14 0 R.ILSPVCGTDGFTYDNECGICAHNAEQR.T

216 - 231 2079.9210 2078.9138 2078.9150 -1 1 K.CRQEIPEIDCDQYPTR.K

218 - 231 1763.7931 1762.7858 1762.7832 1 0 R.QEIPEIDCDQYPTR.K

218 - 232 1891.8902 1890.8829 1890.8782 3 1 R.QEIPEIDCDQYPTRK.T

245 - 276 3604.5843 3603.5770 3603.6545 -21 1 R.ILLPVCGTDGFTYDNECGICAHNAQHGTEVKK.S

**Spot No. 18**

**A. PMF spectrum**

**B. PMF database search result**

Match to: **gi|46195459** Score: **146** Expect: **2.6e-08**

**annexin A1 [Gallus gallus]**

Number of mass values searched: **42**

Number of mass values matched: **15**

Sequence Coverage: **39%**

Matched peptides shown in **Bold Red**

**1** MAMVSEFLKQ AWFMDNQEQE CIKSSK**GGSS VQSRPNFDPS ADVSALDKAI**

**51 TVKGVDEATI IDILTKRTNA QRQQIK**AAYQ QAKGKSLEED LKKVLKSHLE

**101** DVVVALLK**TP AQFDAEELR**A SMK**GLGTDED TLIEILASRN NREIREASRY**

**151 YR**EVLKKDLT QDIISDTSGD FQKALVILAK GDRCEDPHVN DDLADNDAR**A**

**201 LYEAGEKRKG TDVNVFITIL TSRSYPHLRR** AFQKYAKYSK HDMNKVLDLE

**251** LKGDIENCLT ALVKCATSKP AFFAEKLHLA MKGSGTR**HKQ LIR**IMVSRHE

**301** IDLNEIKAYY K**SLYGISLR**Q AIMDELKGDY ETILVALCGS DK

Matched peptides sorted by Residue Number

Start - End Observed Mr(expt) Mr(calc) ppm Miss Sequence

27 - 53 2746.3898 2745.3826 2745.3933 -4 1 K.GGSSVQSRPNFDPSADVSALDKAITVK.G

54 - 67 1543.8672 1542.8599 1542.8617 -1 1 K.GVDEATIIDILTKR.T

68 - 76 1086.6148 1085.6075 1085.5941 12 1 R.TNAQRQQIK.A

109 - 119 1276.6476 1275.6403 1275.6095 24 0 K.TPAQFDAEELR.A

124 - 139 1702.8818 1701.8745 1701.8785 -2 0 K.GLGTDEDTLIEILASR.N

124 - 142 2087.0582 2086.0509 2086.0654 -7 1 K.GLGTDEDTLIEILASRNNR.E

140 - 145 801.4509 800.4436 800.4253 23 1 R.NNREIR.E

146 - 152 944.4860 943.4787 943.4511 29 1 R.EASRYYR.E

200 - 208 1036.5505 1035.5432 1035.5349 8 1 R.ALYEAGEKR.K

209 - 223 1663.9452 1662.9379 1662.9305 4 1 R.KGTDVNVFITILTSR.S

210 - 223 1535.8428 1534.8356 1534.8355 0 0 K.GTDVNVFITILTSR.S

224 - 229 772.4126 771.4053 771.4028 3 0 R.SYPHLR.R

224 - 230 928.5137 927.5064 927.5039 3 1 R.SYPHLRR.A

288 - 293 794.4987 793.4914 793.4922 -1 1 R.HKQLIR.I

312 - 319 908.5144 907.5071 907.5127 -6 0 K.SLYGISLR.Q
