# Supplementary material for: Proteomic analysis of chicken embryonic trachea and kidney tissues after infection in ovo by avian infectious bronchitis coronavirus
Source: Proteome Sci. 2011 Mar 8;9:11. doi: 10.1186/1477-5956-9-11 (PMC3060854; doi:10.1186/1477-5956-9-11)
Supplement: Additional file 3 — Additional_file_3.doc containing the MALDI-TOF spectrum and MALDI-TOF-TOF spectrum of differentially expressed protein spots in IBV-infected chicken embryo kidney tissues. [file 1477-5956-9-11-S3.DOC]

**Additional file 3**

This includes the PMF spectrum and confirmed MALDI-TOF-TOF spectrum of 20 differentially expressed protein spots in IBV-infected chicken embryo kidney tissues. The MALDI-TOF spectra were given by annotating with masses (blue), and their peptide assignments were tabulated with masses. In tables, the peptides with statistically significant ion score (red and bold) was considered to be confidently identified by MALDI-TOF-TOF, and their MS/MS spectra were shown with masses detected and fragment assignments (red). The precursor mass and charge of peptides are shown in bracket, e.g. (1111.5517).

**Spot No. 1 gi|45382533 annexin A2 [Gallus gallus]**

| **Observed Mr** | **Mr(expt)** | **Mr(calc)** | **ppm** | **Start Seq.** | **End Seq.** | **Miss** | **Ion Score** | **Peptide sequence** |
| --- | --- | --- | --- | --- | --- | --- | --- | --- |
| 743.4671 | 742.4598 | 742.4701 | -13.85 | 285 | 290 | 1 |  | R.DKVLIR.I |
| 912.4600 | 911.4527 | 911.4501 | 2.88 | 267 | 273 | 0 |  | K.QLYFADR.L |
| 988.5021 | 987.4948 | 987.4848 | 10.2 | 146 | 152 | 1 |  | R.VYREMYK.T |
| 1004.5000 | 1003.4927 | 1003.4644 | 28.2 | 330 | 339 | 0 |  | R.ALLNLCGGED.- |
| 1050.5571 | 1049.5498 | 1049.5505 | -0.67 | 197 | 205 | 1 |  | R.ELYDAGVKR.K |
| 1058.4674 | 1057.4601 | 1057.4465 | 12.9 | 29 | 37 | 0 |  | K.AYSNFDADR.D |
| 1062.5443 | 1061.5370 | 1061.5328 | 4 | 213 | 220 | 0 |  | K.WINIMTER.S |
| 1078.5514 | 1077.5441 | 1077.5562 | -11.26 | 296 | 304 | 1 |  | R.CEVDMLKIK.S |
| **1111.5517** | **1110.5444** | **1110.5458** | **-1.25** | **69** | **77** | **0** | **70** | **R.QDIAFAYQR.R** |
| 1111.5572 | 1110.5499 | 1110.5458 | 3.7 | 69 | 77 | 0 |  | R.QDIAFAYQR.R |
| 1230.6130 | 1229.6058 | 1229.6000 | 4.69 | 136 | 145 | 0 |  | R.TNQELNEINR.V |
| 1238.6098 | 1237.6025 | 1237.5826 | 16.1 | 105 | 115 | 0 |  | K.TPSQYDASELK.A |
| 1267.6578 | 1266.6505 | 1266.6469 | 2.84 | 69 | 78 | 1 |  | R.QDIAFAYQRR.T |
| 1405.7148 | 1404.7076 | 1404.6925 | 10.7 | 314 | 324 | 0 |  | K.SLYYFIQQDTK.G |
| 1556.8660 | 1555.8587 | 1555.8570 | 1.11 | 50 | 63 | 0 |  | K.GVDEVTIINILTNR.S |
| 1560.7596 | 1559.7523 | 1559.7541 | -1.13 | 234 | 246 | 1 |  | K.SYSPYDMLESIKK.E |
| 1620.9773 | 1619.9701 | 1619.9610 | 5.58 | 89 | 104 | 0 |  | K.SALSGHLEAVILGLLK.T |
| 1648.8403 | 1647.8331 | 1647.8328 | 0.14 | 136 | 148 | 1 |  | R.TNQELNEINRVYR.E |
| 1659.8523 | 1658.8450 | 1658.8450 | -0.01 | 207 | 220 | 1 |  | K.GTDVPKWINIMTER.S |
| **1725.8269** | **1724.8196** | **1724.8230** | **-1.95** | **64** | **77** | **1** | **76** | **R.SNEQRQDIAFAYQR.R** |
| 1725.8398 | 1724.8325 | 1724.8230 | 5.5 | 64 | 77 | 1 |  | R.SNEQRQDIAFAYQR.R |
| 1786.0083 | 1785.0010 | 1784.9996 | 0.79 | 48 | 63 | 1 |  | K.TKGVDEVTIINILTNR.S |
| 1825.8887 | 1824.8814 | 1824.8741 | 3.97 | 153 | 168 | 1 |  | K.TELEKDIISDTSGDFR.K |
| 1884.9882 | 1883.9809 | 1883.9629 | 9.58 | 11 | 28 | 0 |  | K.LSLEGDHSLPPSAYATVK.A |
| 1918.9484 | 1917.9411 | 1917.9618 | -10.79 | 250 | 266 | 0 |  | K.GDLENAFLNLVQCIQNK.Q |
| 2011.9753 | 2010.9680 | 2010.9646 | 1.7 | 29 | 47 | 1 |  | K.AYSNFDADRDAAALEAAIK.T |
| 2024.9700 | 2023.9627 | 2023.9639 | -0.62 | 314 | 329 | 1 |  | K.SLYYFIQQDTKGDYQR.A |

**MS/MS Fragmentation of R.QDIAFAYQR.R**

**MS/MS Fragmentation of R.SNEQRQDIAFAYQR.R**

**Spot No. 2 gi|515694 Tropomyosin beta chain[Gallus gallus]**

| **Observed Mr** | **Mr(expt)** | **Mr(calc)** | **ppm** | **Start Seq.** | **End Seq.** | **Miss** | **Ion Score**  2413.2 | **Peptide sequence** |
| --- | --- | --- | --- | --- | --- | --- | --- | --- |
| 846.4740 | 845.4667 | 845.4606 | 7.18 | 196 | 202 | 1 |  | K.LKEAETR.A |
| 916.4721 | 915.4648 | 915.4661 | -1.4 | 156 | 162 | 0 |  | R.QLEEELR.T |
| 1156.6382 | 1155.6309 | 1155.6499 | -16.47 | 133 | 142 | 0 |  | K.LVVLEGELER.S |
| 1171.6438 | 1170.6365 | 1170.6357 | 0.76 | 154 | 162 | 1 |  | R.VRQLEEELR.T |
| 1243.6490 | 1242.6417 | 1242.6456 | -3.08 | 56 | 65 | 0 |  | R.IQLVEEELDR.A |
| 1262.6100 | 1261.6027 | 1261.5898 | 10.2 | 143 | 153 | 1 |  | R.SEERAEVAESR.V |
| 1284.7455 | 1283.7382 | 1283.7449 | -5.17 | 132 | 142 | 1 |  | R.KLVVLEGELER.S |
| 1308.6375 | 1307.6302 | 1307.6106 | 15 | 198 | 208 | 1 |  | K.EAETRAEFAER.S |
| 1443.7793 | 1442.7720 | 1442.7980 | -18.01 | 70 | 82 | 1 |  | R.LATALQKLEEAEK.A |
| 1719.8423 | 1718.8351 | 1718.8509 | -9.19 | 156 | 169 | 1 |  | R.QLEEELRTMDQSLK.S |
| 1727.8884 | 1726.8811 | 1726.8849 | -2.22 | 56 | 69 | 1 |  | R.IQLVEEELDRAQER.L |
| **1727.9810** | **1726.9737** | **1726.8849** | **51.4** | **56** | **69** | **1** | **55** | **R.IQLVEEELDRAQER.L** |

**MS/MS Fragmentation of R.IQLVEEELDRAQER.L**

**Spot No. 3 gi|45382323 tropomyosin 1 alpha [Gallus gallus]**

| **Observed Mr** | **Mr(expt)** | **Mr(calc)** | **ppm** | **Start Seq.** | **End Seq.** | **Miss** | **Ion Score**  2413.2 | **Peptide sequence** |
| --- | --- | --- | --- | --- | --- | --- | --- | --- |
| 810.4130 | 809.4057 | 809.3807 | 31 | 221 | 226 | 0 |  | K.YEEEIK.V |
| 848.4230 | 847.4157 | 847.4109 | 5.67 | 206 | 213 | 0 |  | K.ALMAAEDK.Y |
| 861.3630 | 860.3557 | 860.3875 | -36.98 | 22 | 29 | 0 |  | R.AEQAEADK.K |
| 906.3730 | 905.3657 | 905.3978 | -35.42 | 52 | 59 | 0 |  | K.GTEDELDK.Y |
| 915.4570 | 914.4497 | 914.4821 | -35.42 | 192 | 198 | 0 |  | R.QLEEQLR.I |
| 940.4890 | 939.4817 | 939.4410 | 43.4 | 153 | 160 | 0 |  | K.HIAEEADR.K |
| 989.5310 | 988.5237 | 988.4825 | 41.7 | 22 | 30 | 1 |  | R.AEQAEADKK.A |
| 1018.5570 | 1017.5497 | 1017.5091 | 40 | 183 | 191 | 0 |  | R.AELSESQVR.Q |
| 1131.6490 | 1130.6417 | 1130.6005 | 36.5 | 141 | 149 | 0 |  | K.MEIQEIQLK.E |
| 1147.5460 | 1146.5387 | 1146.5768 | -33.22 | 50 | 59 | 1 |  | K.LKGTEDELDK.Y |
| 1156.6515 | 1155.6442 | 1155.6499 | -4.95 | 169 | 178 | 0 |  | K.LVIIEGDLER.A |
| 1243.6150 | 1242.6077 | 1242.6456 | -30.44 | 92 | 101 | 0 |  | R.IQLVEEELDR.A |
| 1268.5850 | 1267.5777 | 1267.6156 | -29.9 | 150 | 160 | 1 |  | K.EAKHIAEEADR.K |
| 1284.7504 | 1283.7431 | 1283.7449 | -1.36 | 168 | 178 | 1 |  | R.KLVIIEGDLER.A |
| 1285.7280 | 1284.7207 | 1284.6925 | 22 | 38 | 48 | 0 |  | K.QLEDELVALQK.K |
| 1308.6188 | 1307.6115 | 1307.6106 | 0.71 | 234 | 244 | 1 |  | K.EAETRAEFAER.S |
| 1362.6780 | 1361.6707 | 1361.6423 | 20.9 | 78 | 90 | 0 |  | K.ATDAESEVASLNR.R |
| 1399.7820 | 1398.7747 | 1398.7467 | 20.1 | 91 | 101 | 1 |  | R.RIQLVEEELDR.A |
| 1413.8230 | 1412.8157 | 1412.7875 | 20 | 38 | 49 | 1 |  | K.QLEDELVALQKK.L |
| 1459.7630 | 1458.7557 | 1458.7752 | -13.32 | 141 | 152 | 1 |  | K.MEIQEIQLKEAK.H |
| 1500.8080 | 1499.8007 | 1499.8195 | -12.51 | 36 | 48 | 1 |  | R.SKQLEDELVALQK.K |
| 1518.7320 | 1517.7247 | 1517.7434 | -12.28 | 78 | 91 | 1 |  | K.ATDAESEVASLNRR.I |
| 1641.8724 | 1640.8651 | 1640.8733 | -5.02 | 169 | 182 | 1 |  | K.LVIIEGDLERAEER.A |
| 1677.8360 | 1676.8287 | 1676.8477 | -11.31 | 199 | 213 | 1 |  | R.IMDQTLKALMAAEDK.Y |
| 1727.9129 | 1726.9056 | 1726.8849 | 12 | 92 | 105 | 1 |  | R.IQLVEEELDRAQER.L |
| **1727.9313** | **1726.9240** | **1726.8849** | **22.6** | **92** | **105** | **1** | **56** | **R.IQLVEEELDRAQER.L** |
| 1744.9700 | 1743.9627 | 1743.9189 | 25.1 | 192 | 205 | 1 |  | R.QLEEQLRIMDQTLK.A |
| 1915.0320 | 1914.0247 | 1913.9806 | 23 | 183 | 198 | 1 |  | R.AELSESQVRQLEEQLR.I |
| 2430.1540 | 2429.1467 | 2429.1025 | 18.2 | 265 | 284 | 0 |  | K.EENLNMHQMLDQTLLELNNM.- |

**MS/MS Fragmentation of R.IQLVEEELDRAQER.L**

**Spot No. 4 gi|71895873 annexin 5 [Gallus gallus]**

| **Observed Mr** | **Mr(expt)** | **Mr(calc)** | **ppm** | **Start Seq.** | **End Seq.** | **Miss** | **Ion Score**  2413.2 | **Peptide sequence** |
| --- | --- | --- | --- | --- | --- | --- | --- | --- |
| 755.5466 | 754.5394 | 754.5316 | 10.2 | 236 | 242 | 0 |  | K.LLLAVVK.C |
| 848.4638 | 847.4566 | 847.4552 | 1.62 | 175 | 181 | 0 |  | K.DAQVLFR.A |
| 854.5013 | 853.4941 | 853.4882 | 6.86 | 202 | 208 | 1 |  | R.SVSHLRR.V |
| 873.4790 | 872.4717 | 872.4715 | 0.15 | 19 | 26 | 1 |  | R.ADAEALRK.A |
| 914.5154 | 913.5081 | 913.5021 | 6.57 | 90 | 97 | 0 |  | R.IFDAHALK.H |
| 1001.6042 | 1000.5969 | 1000.5917 | 5.22 | 109 | 117 | 0 |  | K.VLTEILASR.T |
| **1001.6287** | **1000.6214** | **1000.5917** | **29.7** | **109** | **117** | **0** | **75** | **K.VLTEILASR.T** |
| 1073.5924 | 1072.5851 | 1072.5764 | 8.11 | 277 | 285 | 0 |  | R.SEIDLLDIR.H |
| 1133.5546 | 1132.5473 | 1132.5360 | 9.95 | 261 | 271 | 0 |  | K.GAGTDDDTLIR.V |
| 1138.7391 | 1137.7318 | 1137.7234 | 7.42 | 152 | 161 | 0 |  | R.LLVVLLQANR.D |
| 1246.6522 | 1245.6449 | 1245.6275 | 14 | 310 | 321 | 1 |  | R.KALLLLCGGDDE.- |
| 1268.6415 | 1267.6342 | 1267.6197 | 11.4 | 7 | 18 | 0 |  | R.GTVTAFSPFDAR.A |
| **1268.6724** | **1267.6652** | **1267.6197** | **35.9** | **7** | **18** | **0** | **67** | **R.GTVTAFSPFDAR.A** |
| 1476.7592 | 1475.7519 | 1475.7480 | 2.61 | 46 | 58 | 1 |  | R.NNAQRQEIASAFK.T |
| 1614.8233 | 1613.8160 | 1613.9240 | -66.91 | 228 | 242 | 1 |  | R.ETSGDLEKLLLAVVK.C |
| 1642.8716 | 1641.8643 | 1641.8474 | 10.3 | 277 | 289 | 1 |  | R.SEIDLLDIRHEFR.K |
| 1658.9334 | 1657.9261 | 1657.8999 | 15.8 | 102 | 117 | 1 |  | K.GAGTNEKVLTEILASR.T |
| 1678.9744 | 1677.9671 | 1677.9526 | 8.68 | 152 | 166 | 1 |  | R.LLVVLLQANRDPDGR.V |
| 1731.9476 | 1730.9403 | 1730.9203 | 11.6 | 167 | 181 | 1 |  | R.VDEALVEKDAQVLFR.A |
| 1738.8796 | 1737.8723 | 1737.8574 | 8.59 | 187 | 201 | 0 |  | K.WGTDEETFITILGTR.S |
| 1802.8900 | 1801.8828 | 1801.8556 | 15.1 | 213 | 227 | 0 |  | K.YMTISGFQIEETIDR.E |
| 1995.0286 | 1994.0213 | 1993.9857 | 17.9 | 7 | 25 | 1 |  | R.GTVTAFSPFDARADAEALR.K |
| 2166.1186 | 2165.1113 | 2165.1337 | -10.32 | 71 | 89 | 1 |  | K.SELTGKFETLMVSLMRPAR.I |
| 2237.1640 | 2236.1567 | 2236.1376 | 8.57 | 182 | 201 | 1 |  | R.AGELKWGTDEETFITILGTR.S |
| 2292.1574 | 2291.1501 | 2291.1144 | 15.6 | 209 | 227 | 1 |  | R.VFDKYMTISGFQIEETIDR.E |
| 2662.3035 | 2661.2962 | 2661.2480 | 18.1 | 213 | 235 | 1 |  | K.YMTISGFQIEETIDRETSGDLEK.L |
| 2973.3922 | 2972.3850 | 2972.3610 | 8.06 | 127 | 151 | 1 |  | K.QVYMQEYEANLEDKITGETSGHFQR.L |

**MS/MS Fragmentation of K.VLTEILASR.T**

**MS/MS Fragmentation of R.GTVTAFSPFDAR.A**

**Spot No.5 gi|46048696 carbonic anhydrase II [Gallus gallus]**

| **Observed Mr** | **Mr(expt)** | **Mr(calc)** | **ppm** | **Start Seq.** | **End Seq.** | **Miss** | **Ion Score**  2413.2 | **Peptide sequence** |
| --- | --- | --- | --- | --- | --- | --- | --- | --- |
| 1083.6308 | 1082.6235 | 1082.6084 | 14 | 149 | 158 | 0 |  | K.VGNAKPEIQK.V |
| 1187.6775 | 1186.6702 | 1186.6557 | 12.2 | 159 | 169 | 0 |  | K.VVDALNSIQTK.G |
| 1334.7129 | 1333.7056 | 1333.6990 | 4.92 | 77 | 89 | 0 |  | K.SVLQGGALDGVYR.L |
| **1334.8152** | **1333.8079** | **1333.6990** | **81.7** | **77** | **89** | **0** | **80** | **K.SVLQGGALDGVYR.L** |
| 1383.7639 | 1382.7566 | 1382.7381 | 13.4 | 136 | 148 | 0 |  | K.HPDGLAVVGIFMK.V |
| 1399.7687 | 1398.7614 | 1398.7330 | 20.3 | 136 | 148 | 0 |  | K.HPDGLAVVGIFMK.V + Oxidation (M) |
| 1538.6873 | 1537.6800 | 1537.6653 | 9.54 | 227 | 239 | 0 |  | R.GLCFSAENEPVCR.M |
| 1543.7529 | 1542.7456 | 1542.7435 | 1.33 | 240 | 251 | 0 |  | R.MVDNWRPCQPLK.S |
| 1559.7828 | 1558.7756 | 1558.7384 | 23.8 | 240 | 251 | 0 |  | R.MVDNWRPCQPLK.S + Oxidation (M) |
| **1559.8953** | **1558.8880** | **1558.7384** | **95.9** | **240** | **251** | **0** | **23** | **R.MVDNWRPCQPLK.S + Oxidation (M)** |
| 1623.8542 | 1622.8469 | 1622.8205 | 16.3 | 114 | 126 | 0 |  | K.YDAELHIVHWNVK.Y |
| 1807.8732 | 1806.8659 | 1806.8505 | 8.53 | 225 | 239 | 1 |  | K.LRGLCFSAENEPVCR.M |
| 1920.9584 | 1919.9511 | 1919.9200 | 16.2 | 172 | 188 | 0 |  | K.QASFTNFDPTGLLPPCR.D |
| **1921.1044** | **1920.0972** | **1919.9200** | **92.3** | **172** | **188** | **0** | **105** | **K.QASFTNFDPTGLLPPCR.D** |
| 1944.0133 | 1943.0060 | 1942.9676 | 19.8 | 40 | 57 | 0 |  | R.YDPALKPLSFSYDAGTAK.A |
| 2043.1262 | 2042.1189 | 2042.1023 | 8.15 | 130 | 148 | 1 |  | K.FAEALKHPDGLAVVGIFMK.V |
| 2059.1264 | 2058.1191 | 2058.0972 | 10.6 | 130 | 148 | 1 |  | K.FAEALKHPDGLAVVGIFMK.V + Oxidation (M) |
| 2094.9645 | 2093.9573 | 2093.9290 | 13.5 | 58 | 76 | 0 |  | K.AIVNNGHSFNVEFDDSSDK.S |
| 2707.2680 | 2706.2607 | 2706.2609 | -0.07 | 90 | 113 | 0 |  | R.LVQFHIHWGSCEGQGSEHTVDGVK.Y |
| 2912.2357 | 2911.2284 | 2911.4367 | -71.57 | 189 | 212 | 0 |  | R.DYWTYPGSLTTPPLHECVIWHVLK.E |
| 3410.6203 | 3409.6130 | 3409.6175 | -1.32 | 58 | 89 | 1 |  | K.AIVNNGHSFNVEFDDSSDKSVLQGGALDGVYR.L |

**MS/MS Fragmentation of K.SVLQGGALDGVYR.L**

**MS/MS Fragmentation of R.MVDNWRPCQPLK.S**

**MS/MS Fragmentation of K.QASFTNFDPTGLLPPCR.D**

**Spot No. 6 gi|2981970 glutathione S-transferases 2 [Gallus gallus]**

| **Observed Mr** | **Mr(expt)** | **Mr(calc)** | **ppm** | **Start Seq.** | **End Seq.** | **Miss** | **Ion Score**  2413.2 | **Peptide sequence** |
| --- | --- | --- | --- | --- | --- | --- | --- | --- |
| 737.4465 | 736.4392 | 736.4344 | 6.54 | 11 | 17 | 0 |  | R.GLAHAIR.L |
| 740.3754 | 739.3681 | 739.3687 | -0.7 | 193 | 198 | 0 |  | K.ISAYMR.S |
| 1015.5944 | 1014.5872 | 1014.5822 | 4.93 | 69 | 77 | 0 |  | K.LTQSNAILR.Y |
| **1015.6035** | **1014.5962** | **1014.5822** | **13.8** | **69** | **77** | **0** | **58** | **K.LTQSNAILR.Y** |
| 1221.6705 | 1220.6632 | 1220.6554 | 6.4 | 1 | 10 | 0 |  | -.VVTLGYWDIR.G |
| **1221.6885** | **1220.6812** | **1220.6554** | **21.2** | **1** | **10** | **0** | **73** | **.VVTLGYWDIR.G** |
| 1356.8099 | 1355.8027 | 1355.7812 | 15.8 | 122 | 133 | 0 |  | K.LKPAYLEQLPGK.L |
| 1453.7491 | 1452.7418 | 1452.7395 | 1.59 | 96 | 107 | 0 |  | R.VDVLENHLMDLR.M |
| 1457.7419 | 1456.7346 | 1456.7384 | -2.59 | 187 | 198 | 1 |  | R.FEALEKISAYMR.S |
| 1623.8376 | 1622.8303 | 1622.8245 | 3.59 | 205 | 217 | 0 |  | K.APIFWYTALWNNK.K |
| 1654.8473 | 1653.8400 | 1653.8250 | 9.09 | 18 | 30 | 0 |  | R.LLLEYTETPYQER.R |
| **1654.8848** | **1653.8776** | **1653.8250** | **31.8** | **18** | **30** | **0** | **99** | **R.LLLEYTETPYQER.R** |
| 1737.9147 | 1736.9074 | 1736.8992 | 4.75 | 94 | 107 | 1 |  | K.QRVDVLENHLMDLR.M |
| 1746.7992 | 1745.7919 | 1745.7533 | 22.1 | 34 | 49 | 0 |  | K.AGPAPDFDPSDWTNEK.E |
| 1810.9374 | 1809.9301 | 1809.9261 | 2.24 | 18 | 31 | 1 |  | R.LLLEYTETPYQERR.Y |
| 1889.0196 | 1888.0123 | 1887.9982 | 7.48 | 52 | 68 | 0 |  | K.LGLDFPNLPYLIDGDVK.L |
| 1940.0791 | 1939.0719 | 1939.0792 | -3.78 | 1 | 17 | 1 |  | -.VVTLGYWDIRGLAHAIR.L |
| 1942.9967 | 1941.9894 | 1941.9836 | 2.99 | 152 | 167 | 0 |  | K.LTFVDFLAYDVLDQQR.M |
| 2003.9124 | 2002.9051 | 2002.8908 | 7.14 | 34 | 51 | 1 |  | K.AGPAPDFDPSDWTNEKEK.L |
| 2762.3601 | 2761.3529 | 2761.3752 | -8.09 | 145 | 167 | 1 |  | R.SWFVGDKLTFVDFLAYDVLDQQR.M |
| 2885.5753 | 2884.5680 | 2884.5698 | -0.64 | 52 | 77 | 1 |  | K.LGLDFPNLPYLIDGDVKLTQSNAILR.Y |

**MS/MS Fragmentation of K.LTQSNAILR.Y**

**MS/MS Fragmentation of .VVTLGYWDIR.G**

**MS/MS Fragmentation of R.LLLEYTETPYQER.R**

**Spot No. 7 gi|50751518 PREDICTED: similar to natural killer cell enhancing factor isoform 4**

| **Observed Mr** | **Mr(expt)** | **Mr(calc)** | **ppm** | **Start Seq.** | **End Seq.** | **Miss** | **Ion Score**  2413.2 | **Peptide sequence** |
| --- | --- | --- | --- | --- | --- | --- | --- | --- |
| 819.4249 | 818.4176 | 818.4134 | 5.17 | 152 | 158 | 0 |  | R.SVDETLR.L |
| 934.5300 | 933.5227 | 933.5171 | 6.01 | 129 | 136 | 0 |  | R.GLFIIDEK.G |
| 952.4507 | 951.4434 | 951.4297 | 14.4 | 121 | 128 | 0 |  | K.EDEGIAYR.G |
| 1028.6145 | 1027.6072 | 1027.6026 | 4.47 | 102 | 110 | 1 |  | K.IPLVSDTKR.V |
| 1196.6329 | 1195.6256 | 1195.6237 | 1.6 | 159 | 168 | 0 |  | R.LVQAFQFTDK.H |
| 1225.7051 | 1224.6978 | 1224.6826 | 12.4 | 141 | 151 | 0 |  | R.QITINDLPVGR.S |
| **1225.7191** | **1224.7119** | **1224.6826** | **23.9** | **141** | **151** | **0** | **85** | **R.QITINDLPVGR.S** |
| 1627.8269 | 1626.8197 | 1626.7889 | 18.9 | 115 | 128 | 1 |  | K.DYGVLKEDEGIAYR.G |
| **1627.8285** | **1626.8212** | **1626.7889** | **19.9** | **115** | **128** | **1** | **99** | **K.DYGVLKEDEGIAYR.G** |
| 2309.1838 | 2308.1765 | 2308.1562 | 8.81 | 6 | 27 | 0 |  | K.AFIGKPAPDFTATAVMPDGQFK.D |
| 2665.3931 | 2664.3858 | 2664.3622 | 8.87 | 6 | 30 | 1 |  | K.AFIGKPAPDFTATAVMPDGQFKDIK.L |

**MS/MS Fragmentation of R.QITINDLPVGR.S**

**MS/MS Fragmentation of K.DYGVLKEDEGIAYR.G**

**Spot No.8 gi|71895267 carbonyl reductase 1 [Gallus gallus]**

| **Observed Mr** | **Mr(expt)** | **Mr(calc)** | **ppm** | **Start Seq.** | **End Seq.** | **Miss** | **Ion Score**  2413.2 | **Peptide sequence** |
| --- | --- | --- | --- | --- | --- | --- | --- | --- |
| 790.4695 | 789.4622 | 789.4167 | 57.7 | 210 | 215 | 1 |  | R.MLNEKR.K |
| 798.5823 | 797.5750 | 797.5123 | 78.6 | 15 | 22 | 0 |  | K.GIGLAIVR.D |
| 844.5902 | 843.5829 | 843.5178 | 77.2 | 198 | 205 | 0 |  | K.IGVTVLSR.I |
| 890.6057 | 889.5985 | 889.5385 | 67.4 | 71 | 77 | 1 |  | K.VLRDFLK.E |
| 1297.7360 | 1296.7287 | 1296.6826 | 35.6 | 27 | 37 | 1 |  | K.QFKGDVYLTAR.D |
| 1303.7726 | 1302.7653 | 1302.7184 | 36.1 | 100 | 111 | 0 |  | R.TPFAVQAEVTLK.T |
| 1308.6799 | 1307.6726 | 1307.6146 | 44.4 | 186 | 197 | 0 |  | K.EGWPNTAYGVSK.I |
| 1314.8226 | 1313.8153 | 1313.7489 | 50.5 | 15 | 26 | 1 |  | K.GIGLAIVRDLCK.Q |
| 1333.7287 | 1332.7215 | 1332.6786 | 32.2 | 30 | 41 | 1 |  | K.GDVYLTARDPAR.G |
| 1649.9281 | 1648.9208 | 1648.8937 | 16.5 | 80 | 95 | 0 |  | K.YGGLNVLVNNAGIAFK.V |
| 1723.8619 | 1722.8547 | 1722.8233 | 18.2 | 159 | 173 | 0 |  | R.SDTITEDELVELMTK.F |
| 1760.9786 | 1759.9713 | 1759.9469 | 13.9 | 96 | 111 | 1 |  | K.VSDRTPFAVQAEVTLK.T |
| 1767.8547 | 1766.8474 | 1766.8345 | 7.32 | 217 | 231 | 0 |  | K.GDHILLNACCPGWVR.T |
| **1786.9971** | **1785.9899** | **1785.9811** | **4.92** | **119** | **133** | **0** | **67** | **R.NICTELLPLIKPYGR.V** |
| 1787.0186 | 1786.0113 | 1785.9811 | 16.9 | 119 | 133 | 0 |  | R.NICTELLPLIKPYGR.V |
| 1888.9427 | 1887.9354 | 1887.9115 | 12.7 | 181 | 197 | 1 |  | K.SVHEKEGWPNTAYGVSK.I |
| **1895.9391** | **1894.9318** | **1894.9294** | **1.26** | **216** | **231** | **1** | **57** | **R.KGDHILLNACCPGWVR.T** |
| 1895.9533 | 1894.9460 | 1894.9294 | 8.74 | 216 | 231 | 1 |  | R.KGDHILLNACCPGWVR.T |
| 1907.0494 | 1906.0421 | 1906.0312 | 5.7 | 78 | 95 | 1 |  | K.EKYGGLNVLVNNAGIAFK.V |
| 2027.0085 | 2026.0013 | 2025.9929 | 4.14 | 157 | 173 | 1 |  | K.FRSDTITEDELVELMTK.F |
| 2043.0068 | 2041.9995 | 2041.9878 | 5.76 | 157 | 173 | 1 |  | K.FRSDTITEDELVELMTK.F + Oxidation (M) |
| 2127.1226 | 2126.1154 | 2126.1161 | -0.33 | 100 | 118 | 1 |  | R.TPFAVQAEVTLKTNFFGTR.N |
| 2293.1447 | 2292.1375 | 2292.1454 | -3.45 | 134 | 155 | 0 |  | R.VVNVSSMVSISALGGCSQELQK.K |
| 2309.1437 | 2308.1364 | 2308.1403 | -1.68 | 134 | 155 | 0 |  | R.VVNVSSMVSISALGGCSQELQK.K + Oxidation (M) |
| 2588.3018 | 2587.2946 | 2587.3282 | -13 | 49 | 70 | 0 |  | K.LQEEGLHPLFHQLDIDDLQSIK.V |
| 3140.4208 | 3139.4135 | 3139.4986 | -27.11 | 242 | 271 | 0 |  | K.SPEEGAETPVYLALLPSDADGPHGQFVSEK.T |

**MS/MS Fragmentation of R.NICTELLPLIKPYGR.V**

**MS/MS Fragmentation of R.KGDHILLNACCPGWVR.T**

**Spot No.9 gi|45382969 sulfotransferase family, cytosolic, 1C, member 3 [Gallus gallus]**

| **Observed Mr** | **Mr(expt)** | **Mr(calc)** | **ppm** | **Start Seq.** | **End Seq.** | **Miss** | **Ion Score**  2413.2 | **Peptide sequence** |
| --- | --- | --- | --- | --- | --- | --- | --- | --- |
| 748.3667 | 747.3594 | 747.3552 | 5.71 | 271 | 277 | 0 |  | K.GSVGDWK.N |
| 943.4727 | 942.4654 | 942.4447 | 22 | 288 | 294 | 1 |  | K.FDQDYKK.K |
| 971.4972 | 970.4899 | 970.4906 | -0.68 | 107 | 115 | 0 |  | K.LAEAMPSPR.T |
| 1039.5317 | 1038.5244 | 1038.5168 | 7.31 | 296 | 304 | 0 |  | K.MADTSLVFR.M |
| 1069.5682 | 1068.5609 | 1068.5604 | 0.49 | 278 | 286 | 0 |  | K.NYFTVALNK.K |
| 1432.7760 | 1431.7688 | 1431.7722 | -2.27 | 44 | 56 | 0 |  | K.ARPDDLLVATYAK.A |
| **1432.8114** | **1431.8041** | **1431.7722** | **22.3** | **44** | **56** | **0** | **47** | **K.ARPDDLLVATYAK.A** |
| 1450.6928 | 1449.6856 | 1449.6789 | 4.58 | 146 | 156 | 0 |  | K.DNLVSYYHFHR.M |
| **1450.7288** | **1449.7216** | **1449.6789** | **29.4** | **146** | **156** | **0** | **86** | **K.DNLVSYYHFHR.M** |
| 1574.8081 | 1573.8008 | 1573.7876 | 8.41 | 160 | 173 | 0 |  | K.VLPDPGTIEEFTEK.F |
| 1591.8254 | 1590.8181 | 1590.8141 | 2.53 | 222 | 234 | 1 |  | K.FLEKDLDEEVLNK.I |
| 1723.7851 | 1722.7778 | 1722.7858 | -4.67 | 255 | 269 | 0 |  | K.DFVGVMDHSVSPFMR.K |
| 1802.8962 | 1801.8889 | 1801.8960 | -3.93 | 200 | 213 | 1 |  | R.ILYLFYEDMKENPK.R |
| 1947.9381 | 1946.9308 | 1946.9659 | -18.02 | 157 | 173 | 1 |  | R.MNKVLPDPGTIEEFTEK.F |
| 2306.1368 | 2305.1296 | 2305.1419 | -5.35 | 87 | 106 | 0 |  | R.HPFLEWYIPDSSPLGYSGLK.L |
| 2462.2292 | 2461.2219 | 2461.2430 | -8.58 | 86 | 106 | 1 |  | K.RHPFLEWYIPDSSPLGYSGLK.L |

**MS/MS Fragmentation of K.ARPDDLLVATYAK.A**

**MS/MS Fragmentation of K.DNLVSYYHFHR.M**

**Spot No. 10 gi|45383766 L-lactate dehydrogenase B [Gallus gallus]**

| **Observed Mr** | **Mr(expt)** | **Mr(calc)** | **ppm** | **Start Seq.** | **End Seq.** | **Miss** | **Ion Score**  2413.2 | **Peptide sequence** |
| --- | --- | --- | --- | --- | --- | --- | --- | --- |
| 742.4527 | 741.4454 | 741.4497 | -5.82 | 107 | 112 | 0 |  | R.LNLVQR.N |
| 782.3849 | 781.3776 | 781.3792 | -2.06 | 172 | 177 | 0 |  | R.YLMAER.L |
| 833.3711 | 832.3638 | 832.3675 | -4.38 | 100 | 106 | 0 |  | R.QQEGESR.L |
| 913.5801 | 912.5729 | 912.5757 | -3.08 | 91 | 99 | 0 |  | K.IVVVTAGVR.Q |
| **913.6326** | **912.6253** | **912.5757** | **54.4** | **91** | **99** | **0** | **40** | **K.IVVVTAGVR.Q** |
| 957.6140 | 956.6068 | 956.6059 | 0.91 | 119 | 126 | 0 |  | K.FIIPQIVK.Y |
| 969.5883 | 968.5810 | 968.5655 | 16 | 270 | 278 | 0 |  | R.VHSVSTLVK.G |
| 1085.5588 | 1084.5515 | 1084.5488 | 2.57 | 170 | 177 | 1 |  | R.FRYLMAER.L |
| 1148.5972 | 1147.5899 | 1147.5873 | 2.25 | 319 | 328 | 0 |  | K.SADTLWSIQK.D |
| 1158.6449 | 1157.6376 | 1157.6292 | 7.3 | 308 | 317 | 1 |  | K.LKDDEVAQLK.K |
| 1276.7310 | 1275.7238 | 1275.6823 | 32.5 | 318 | 328 | 1 |  | K.KSADTLWSIQK.D |
| 1292.6888 | 1291.6815 | 1291.6772 | 3.33 | 233 | 243 | 0 |  | K.QVVESAYEVIR.L |
| **1292.7661** | **1291.7588** | **1291.6772** | **63.2** | **233** | **243** | **0** | **58** | **K.QVVESAYEVIR.L** |
| 1454.8403 | 1453.8330 | 1453.8140 | 13 | 8 | 22 | 0 |  | K.LITPVAAGSTVPSNK.I |
| 1494.7975 | 1493.7902 | 1493.7726 | 11.8 | 77 | 90 | 1 |  | K.IVADKDYAVTANSK.I |
| 1504.7806 | 1503.7733 | 1503.7933 | -13.28 | 319 | 331 | 1 |  | K.SADTLWSIQKDLK.D |
| 1556.8183 | 1555.8110 | 1555.8066 | 2.82 | 100 | 112 | 1 |  | R.QQEGESRLNLVQR.N |
| 1711.9503 | 1710.9431 | 1710.9516 | -4.98 | 6 | 22 | 1 |  | K.EKLITPVAAGSTVPSNK.I |
| 1727.9509 | 1726.9436 | 1726.9326 | 6.38 | 91 | 106 | 1 |  | K.IVVVTAGVRQQEGESR.L |
| 1785.9508 | 1784.9435 | 1784.9421 | 0.82 | 229 | 243 | 1 |  | K.EVHKQVVESAYEVIR.L |
| 1972.0029 | 1970.9956 | 1970.9343 | 31.1 | 60 | 76 | 0 |  | K.GEMMDLQHGSLFLQTHK.I |
| 2213.1191 | 2212.1118 | 2212.1133 | -0.66 | 58 | 76 | 1 |  | K.LKGEMMDLQHGSLFLQTHK.I |

**MS/MS Fragmentation of K.IVVVTAGVR.Q**

**MS/MS Fragmentation of K.QVVESAYEVIR.L**

**Spot No.11 gi|118090053 similar to L-3-hydroxyacyl-Coenzyme A dehydrogenase, short chain [Gallus gallus]**

| **Observed Mr** | **Mr(expt)** | **Mr(calc)** | **ppm** | **Start Seq.** | **End Seq.** | **Miss** | **Ion Score**  2413.2 | **Peptide sequence** |
| --- | --- | --- | --- | --- | --- | --- | --- | --- |
| 929.5613 | 928.5541 | 928.4654 | 95.5 | 306 | 313 | 1 |  | R.KTGEGFYK.Y |
| 931.5205 | 930.5132 | 930.5134 | -0.23 | 70 | 77 | 1 |  | K.GIEESLKR.V |
| 1018.5401 | 1017.5329 | 1017.5244 | 8.37 | 214 | 222 | 0 |  | K.DTPGFIVNR.L |
| 1078.4833 | 1077.4760 | 1077.4767 | -0.62 | 307 | 315 | 1 |  | K.TGEGFYKYN.- |
| 1195.5592 | 1194.5520 | 1194.6332 | -67.99 | 1 | 10 | 0 |  | -.MAFATRPFVR.A |
| 1220.5422 | 1219.5350 | 1219.5431 | -6.55 | 194 | 203 | 0 |  | K.TYESLMDFSK.A |
| 1321.7057 | 1320.6984 | 1320.6934 | 3.81 | 223 | 233 | 0 |  | R.LLVPYMMEAVR.L |
| **1321.7723** | **1320.7650** | **1320.6934** | **54.3** | **223** | **233** | **0** | **38** | **R.LLVPYMMEAVR.L** |
| 1551.7591 | 1550.7518 | 1550.7616 | -6.34 | 83 | 96 | 0 |  | K.FADKPEAGAEFIEK.T |
| 1621.8013 | 1620.7941 | 1620.7945 | -0.28 | 167 | 180 | 0 |  | R.FGGLHFFNPVPMMK.L |
| 1866.8551 | 1865.8479 | 1865.9895 | -75.93 | 223 | 237 | 1 |  | R.LLVPYMMEAVRLFER.G |
| 2289.0172 | 2288.0099 | 2288.2214 | -92.43 | 167 | 186 | 1 |  | R.FGGLHFFNPVPMMKLVEVVK.T |
| 2865.4237 | 2864.4164 | 2864.4304 | -4.91 | 138 | 163 | 0 |  | K.FAPEHTIFTSNTSSLQITQLANSTTR.Q |
| 3221.6421 | 3220.6348 | 3220.6364 | -0.5 | 135 | 163 | 1 |  | R.LDKFAPEHTIFTSNTSSLQITQLANSTTR.Q |

**MS/MS Fragmentation of  R.LLVPYMMEAVR.L**

**Spot No.12 gi|45382533 annexin A2 [Gallus gallus]**

| **Observed Mr** | **Mr(expt)** | **Mr(calc)** | **ppm** | **Start Seq.** | **End Seq.** | **Miss** | **Ion Score**  2413.2 | **Peptide sequence** |
| --- | --- | --- | --- | --- | --- | --- | --- | --- |
| 743.4768 | 742.4696 | 742.4701 | -0.75 | 285 | 290 | 1 |  | R.DKVLIR.I |
| 756.3716 | 755.3643 | 755.3524 | 15.9 | 274 | 279 | 0 |  | R.LYDSMK.G |
| 912.4536 | 911.4463 | 911.4501 | -4.14 | 267 | 273 | 0 |  | K.QLYFADR.L |
| 988.4679 | 987.4606 | 987.4848 | -24.45 | 146 | 152 | 1 |  | R.VYREMYK.T |
| 1004.4902 | 1003.4830 | 1003.4644 | 18.5 | 330 | 339 | 0 |  | R.ALLNLCGGED.- |
| 1050.5506 | 1049.5434 | 1049.5505 | -6.83 | 197 | 205 | 1 |  | R.ELYDAGVKR.K |
| 1058.4471 | 1057.4398 | 1057.4465 | -6.27 | 29 | 37 | 0 |  | K.AYSNFDADR.D |
| 1062.5324 | 1061.5251 | 1061.5328 | -7.18 | 213 | 220 | 0 |  | K.WINIMTER.S |
| 1078.5373 | 1077.5300 | 1077.5562 | -24.33 | 296 | 304 | 1 |  | R.CEVDMLKIK.S |
| 1111.5475 | 1110.5402 | 1110.5458 | -5.02 | 69 | 77 | 0 |  | R.QDIAFAYQR.R |
| **1111.5547** | **1110.5474** | **1110.5458** | **1.43** | **69** | **77** | **0** | **74** | **R.QDIAFAYQR.R** |
| 1230.6007 | 1229.5934 | 1229.6000 | -5.38 | 136 | 145 | 0 |  | R.TNQELNEINR.V |
| 1238.5858 | 1237.5785 | 1237.5826 | -3.3 | 105 | 115 | 0 |  | K.TPSQYDASELK.A |
| 1267.6453 | 1266.6380 | 1266.6469 | -7.01 | 69 | 78 | 1 |  | R.QDIAFAYQRR.T |
| 1405.6965 | 1404.6892 | 1404.6925 | -2.36 | 314 | 324 | 0 |  | K.SLYYFIQQDTK.G |
| 1556.8536 | 1555.8463 | 1555.8570 | -6.86 | 50 | 63 | 0 |  | K.GVDEVTIINILTNR.S |
| **1556.8837** | **1555.8765** | **1555.8570** | **12.5** | **50** | **63** | **0** | **108** | **K.GVDEVTIINILTNR.S** |
| 1560.7321 | 1559.7248 | 1559.7541 | -18.76 | 234 | 246 | 1 |  | K.SYSPYDMLESIKK.E |
| 1620.9610 | 1619.9537 | 1619.9610 | -4.52 | 89 | 104 | 0 |  | K.SALSGHLEAVILGLLK.T |
| 1648.8213 | 1647.8140 | 1647.8328 | -11.44 | 136 | 148 | 1 |  | R.TNQELNEINRVYR.E |
| 1659.8348 | 1658.8275 | 1658.8450 | -10.54 | 207 | 220 | 1 |  | K.GTDVPKWINIMTER.S |
| 1725.8337 | 1724.8264 | 1724.8230 | 1.97 | 64 | 77 | 1 |  | R.SNEQRQDIAFAYQR.R |
| **1725.8773** | **1724.8701** | **1724.8230** | **27.3** | **64** | **77** | **1** | **74** | **R.SNEQRQDIAFAYQR.R** |
| 1785.9985 | 1784.9912 | 1784.9996 | -4.7 | 48 | 63 | 1 |  | K.TKGVDEVTIINILTNR.S |
| 1825.8698 | 1824.8626 | 1824.8741 | -6.34 | 153 | 168 | 1 |  | K.TELEKDIISDTSGDFR.K |
| 1884.9649 | 1883.9576 | 1883.9629 | -2.8 | 11 | 28 | 0 |  | K.LSLEGDHSLPPSAYATVK.A |
| 1918.9441 | 1917.9369 | 1917.9618 | -13.02 | 250 | 266 | 0 |  | K.GDLENAFLNLVQCIQNK.Q |
| 2011.9669 | 2010.9596 | 2010.9646 | -2.48 | 29 | 47 | 1 |  | K.AYSNFDADRDAAALEAAIK.T |
| 2024.9579 | 2023.9506 | 2023.9639 | -6.6 | 314 | 329 | 1 |  | K.SLYYFIQQDTKGDYQR.A |

**MS/MS Fragmentation of R.QDIAFAYQR.R**

**MS/MS Fragmentation of K.GVDEVTIINILTNR.S**

**MS/MS Fragmentation of R.SNEQRQDIAFAYQR.R**

**Spot No. 13 gi|118094764 PREDICTED: similar to cystathionase [Gallus gallus]**

| **Observed Mr** | **Mr(expt)** | **Mr(calc)** | **ppm** | **Start Seq.** | **End Seq.** | **Miss** | **Ion Score**  2413.2 | **Peptide sequence** |
| --- | --- | --- | --- | --- | --- | --- | --- | --- |
| 796.3548 | 795.3475 | 795.3399 | 9.61 | 224 | 229 | 0 |  | R.DDVYER.L |
| 829.5034 | 828.4961 | 828.4858 | 12.5 | 318 | 324 | 0 |  | K.HATIFLK.S |
| 832.3924 | 831.3851 | 831.3810 | 4.97 | 260 | 265 | 0 |  | R.MNQHFR.N |
| **862.4283** | **861.4210** | **861.4344** | **-15.59** | **273** | **279** | **0** | **48** | **R.FLESNPR.V** |
| 862.4454 | 861.4381 | 861.4344 | 4.28 | 273 | 279 | 0 |  | R.FLESNPR.V |
| 1187.6647 | 1186.6574 | 1186.6557 | 1.44 | 359 | 369 | 0 |  | R.EALGISDTLIR.L |
| **1187.6802** | **1186.6729** | **1186.6557** | **14.5** | **359** | **369** | **0** | **109** | **R.EALGISDTLIR.L** |
| 1218.6374 | 1217.6302 | 1217.6404 | -8.42 | 273 | 282 | 1 |  | R.FLESNPRVEK.V |
| 1417.6995 | 1416.6923 | 1416.7646 | -51.08 | 63 | 76 | 1 |  | R.TCLEKAVAVLDGAK.Y |
| 1503.8511 | 1502.8438 | 1502.8345 | 6.2 | 28 | 42 | 0 |  | R.SGALVPPVSLSTTFK.Q |
| 1511.8531 | 1510.8458 | 1510.8395 | 4.14 | 147 | 159 | 0 |  | K.LVWIETPTNPTLK.V |
| 1543.8262 | 1542.8189 | 1542.8266 | -5.01 | 266 | 279 | 1 |  | R.NALAVGRFLESNPR.V |
| 1578.6988 | 1577.6915 | 1577.6859 | 3.58 | 43 | 56 | 0 |  | K.QQAPGEHAGYDYSR.C |
| **1578.7069** | **1577.6996** | **1577.6859** | **8.69** | **43** | **56** | **0** | **120** | **K.QQAPGEHAGYDYSR.C** |
| 1601.8338 | 1600.8265 | 1600.8420 | -9.66 | 356 | 369 | 1 |  | K.EEREALGISDTLIR.L |
| 1785.9737 | 1784.9665 | 1784.9573 | 5.11 | 283 | 298 | 0 |  | K.VIYPGLPSHPQHELAK.R |
| 1941.9958 | 1940.9885 | 1941.0584 | -36.04 | 283 | 299 | 1 |  | K.VIYPGLPSHPQHELAKR.Q |
| 2244.1183 | 2243.1111 | 2243.1056 | 2.43 | 370 | 389 | 0 |  | R.LSVGLEDEEDLLEDLDQALK.A |

**MS/MS Fragmentation of R.FLESNPR.V**

**MS/MS Fragmentation of R.EALGISDTLIR.L**

**MS/MS Fragmentation of K.QQAPGEHAGYDYSR.C**

**Spot No. 14 gi|118093509 PREDICTED: similar to cytosolic NADP-dependent isocitrate dehydrogenase [Gallus gallus]**

| **Observed Mr** | **Mr(expt)** | **Mr(calc)** | **ppm** | **Start Seq.** | **End Seq.** | **Miss** | **Ion Score**  2413.2 | **Peptide sequence** |
| --- | --- | --- | --- | --- | --- | --- | --- | --- |
| 744.4266 | 743.4193 | 743.3926 | 35.9 | 94 | 100 | 0 |  | K.SPNGTIR.N |
| 756.4330 | 755.4257 | 755.4290 | -4.38 | 382 | 388 | 0 |  | K.GLPNVTR.S |
| 892.4643 | 891.4571 | 891.5065 | -55.5 | 83 | 89 | 1 |  | R.VEEFKLK.Q |
| 900.5570 | 899.5497 | 899.5480 | 1.91 | 21 | 27 | 0 |  | R.VIWELIK.E |
| 903.4484 | 902.4411 | 902.4399 | 1.42 | 244 | 249 | 0 |  | K.IWYEHR.L |
| 976.5570 | 975.5498 | 975.5502 | -0.42 | 101 | 109 | 0 |  | R.NILGGTVFR.E |
| **976.5909** | **975.5837** | **975.5502** | **34.3** | **101** | **109** | **0** | **71** | **R.NILGGTVFR.E** |
| 1009.4484 | 1008.4412 | 1008.4413 | -0.17 | 133 | 140 | 0 |  | R.HAYGDQYR.A |
| 1031.5431 | 1030.5358 | 1030.5348 | 0.98 | 243 | 249 | 1 |  | K.KIWYEHR.L |
| **1031.5520** | **1030.5447** | **1030.5348** | **9.62** | **243** | **249** | **1** | **43** | **K.KIWYEHR.L** |
| 1087.5875 | 1086.5802 | 1086.5710 | 8.48 | 141 | 151 | 0 |  | R.ATDFVVPGPGK.V |
| 1103.5765 | 1102.5692 | 1102.5982 | -26.32 | 344 | 353 | 1 |  | R.AKLDNNTSLK.T |
| 1198.5925 | 1197.5852 | 1197.5666 | 15.6 | 225 | 233 | 0 |  | K.DIFQEIYDR.E |
| 1216.6566 | 1215.6493 | 1215.6533 | -3.28 | 250 | 260 | 0 |  | R.LIDDMVAQALK.S |
| 1341.6820 | 1340.6747 | 1340.6684 | 4.66 | 302 | 314 | 0 |  | K.TVEAEAAHGTVTR.H |
| 1423.8801 | 1422.8729 | 1422.8711 | 1.24 | 120 | 132 | 0 |  | R.LVSGWVKPIVIGR.H |
| **1423.9378** | **1422.9305** | **1422.8711** | **41.7** | **120** | **132** | **0** | **106** | **R.LVSGWVKPIVIGR.H** |
| 1473.7398 | 1472.7325 | 1472.7300 | 1.75 | 223 | 233 | 1 |  | R.FKDIFQEIYDR.E |
| 1509.6787 | 1508.6714 | 1508.6493 | 14.6 | 389 | 400 | 0 |  | R.SDYLNTFEFMDK.L |
| 1618.7788 | 1617.7715 | 1617.7675 | 2.51 | 225 | 236 | 1 |  | K.DIFQEIYDREYK.S |
| 1745.7973 | 1744.7901 | 1744.7873 | 1.61 | 5 | 20 | 0 |  | K.IHGGSVVEMQGDEMTR.V |
| 1873.8781 | 1872.8708 | 1872.8822 | -6.09 | 4 | 20 | 1 |  | K.KIHGGSVVEMQGDEMTR.V |
| 1878.9332 | 1877.9260 | 1877.9272 | -0.64 | 322 | 338 | 0 |  | K.GQETSTNPIASIFAWTR.G |
| 2120.0302 | 2119.0229 | 2119.0296 | -3.12 | 389 | 406 | 1 |  | R.SDYLNTFEFMDKLAANLK.G |
| 2415.2088 | 2414.2015 | 2414.2270 | -10.57 | 30 | 49 | 0 |  | K.LIFPYVDLDLHSYDLGIEHR.D |

**MS/MS Fragmentation of R.NILGGTVFR.E**

**MS/MS Fragmentation of K.KIWYEHR.L**

**MS/MS Fragmentation of R.LVSGWVKPIVIGR.H**

**Spot No. 15 gi|46048768 enolase 1 [Gallus gallus]**

| **Observed Mr** | **Mr(expt)** | **Mr(calc)** | **ppm** | **Start Seq.** | **End Seq.** | **Miss** | **Ion Score** | **Peptide sequence** |
| --- | --- | --- | --- | --- | --- | --- | --- | --- |
| 748.3541 | 747.3468 | 747.3511 | -5.76 | 51 | 56 | 1 |  | R.DNDKTR.Y |
| 766.3693 | 765.3620 | 765.3657 | -4.83 | 10 | 15 | 0 |  | R.EIFDSR.G |
| 796.3986 | 795.3913 | 795.4239 | -40.93 | 65 | 71 | 0 |  | K.AVEHVNK.T |
| 803.4273 | 802.4200 | 802.4198 | 0.31 | 427 | 432 | 1 |  | R.NFRNPR.I |
| 806.4522 | 805.4449 | 805.4446 | 0.37 | 407 | 412 | 0 |  | K.YNQLLR.I |
| 913.5600 | 912.5527 | 912.5644 | -12.79 | 72 | 80 | 0 |  | K.TIAPALISK.N |
| 1100.5169 | 1099.5096 | 1099.5186 | -8.16 | 254 | 262 | 1 |  | R.DGKYDLDFK.S |
| 1143.6188 | 1142.6116 | 1142.6084 | 2.78 | 184 | 193 | 0 |  | R.IGAEVYHNLK.N |
| 1414.7526 | 1413.7453 | 1413.7463 | -0.72 | 81 | 92 | 1 |  | K.NVNVVEQEKIDK.L |
| 1449.7193 | 1448.7120 | 1448.7147 | -1.87 | 16 | 28 | 0 |  | R.GNPTVEVDLYTNK.G |
| 1541.7569 | 1540.7497 | 1540.7569 | -4.68 | 359 | 372 | 0 |  | K.LAQSNGWGVMVSHR.S |
| 1556.7781 | 1555.7708 | 1555.7705 | 0.23 | 240 | 253 | 0 |  | K.VVIGMDVAASEFYR.D |
| 1597.8180 | 1596.8107 | 1596.8987 | -55.13 | 184 | 197 | 1 |  | R.IGAEVYHNLKNVIK.E |
| 1691.8781 | 1690.8708 | 1690.8889 | -10.74 | 407 | 420 | 1 |  | K.YNQLLRIEEELGSK.A |
| 1804.9518 | 1803.9445 | 1803.9366 | 4.35 | 33 | 50 | 0 |  | R.AAVPSGASTGIYEALELR.D |
| **1805.0013** | **1803.9941** | **1803.9366** | **31.8** | **33** | **50** | **0** | **160** | **R.AAVPSGASTGIYEALELR.D** |
| 1960.9320 | 1959.9248 | 1959.9174 | 3.76 | 203 | 221 | 0 |  | K.DATNVGDEGGFAPNILENK.E |
| 2061.0900 | 2060.0827 | 2060.0790 | 1.81 | 307 | 326 | 0 |  | K.FTASVGIQVVGDDLTVTNPK.R |
| 2178.05 | 2177.0428 | 2177.0463 | -1.63 | 234 | 253 | 1 |  | K.AGYSDKVVIGMDVAASEFYR.D |
| 2197.0883 | 2196.0810 | 2196.0699 | 5.06 | 10 | 28 | 1 |  | R.EIFDSRGNPTVEVDLYTNK.G |
| 2217.1890 | 2216.1818 | 2216.1801 | 0.73 | 307 | 327 | 1 |  | K.FTASVGIQVVGDDLTVTNPKR.I |
| 2277.1394 | 2276.1321 | 2276.1284 | 1.62 | 33 | 54 | 1 |  | R.AAVPSGASTGIYEALELRDNDK.T |
| 2309.1339 | 2308.1266 | 2308.0971 | 12.8 | 200 | 221 | 1 |  | K.YGKDATNVGDEGGFAPNILENK.E |
| 2523.1879 | 2522.1806 | 2522.1754 | 2.07 | 286 | 306 | 1 |  | K.NYPVVSIEDPFDQDDWAAWKK.F |
| 2757.3993 | 2756.3920 | 2756.3868 | 1.88 | 203 | 228 | 1 |  | K.DATNVGDEGGFAPNILENKEALELLK.T |
| 3021.5824 | 3020.5751 | 3020.5832 | -2.69 | 133 | 162 | 0 |  | R.HIADLAGNPEVILPVPAFNVINGGSHAGNK.L |

**MS/MS Fragmentation of R.AAVPSGASTGIYEALELR.D**

**Spot No.16 gi|57530409 CNDP dipeptidase 2 [Gallus gallus]**

| **Observed Mr** | **Mr(expt)** | **Mr(calc)** | **ppm** | **Start Seq.** | **End Seq.** | **Miss** | **Ion Score**  2413.2 | **Peptide sequence** |
| --- | --- | --- | --- | --- | --- | --- | --- | --- |
| 784.4123 | 783.4050 | 783.4061 | -1.4 | 303 | 308 | 0 |  | R.DILMHR.W |
| 808.4305 | 807.4232 | 807.4239 | -0.87 | 122 | 128 | 1 |  | R.DGKLYGR.G |
| 882.5103 | 881.5030 | 881.5083 | -5.97 | 296 | 302 | 1 |  | K.LLHDTKR.D |
| 984.5217 | 983.5144 | 983.5076 | 6.88 | 454 | 461 | 0 |  | R.YNYIQGVK.M |
| 1161.6133 | 1160.6060 | 1160.6189 | -11.17 | 364 | 373 | 1 |  | K.KFAELQSPNK.F |
| 1164.5908 | 1163.5835 | 1163.6121 | -24.55 | 202 | 211 | 0 |  | K.NKPCITYGLR.G |
| 1189.6224 | 1188.6151 | 1188.6139 | 1.02 | 150 | 159 | 0 |  | K.TNQEFPVNIK.F |
| 1233.6482 | 1232.6409 | 1232.6401 | 0.67 | 403 | 413 | 0 |  | K.TVFGVEPDLTR.E |
| 1241.6435 | 1240.6362 | 1240.6339 | 1.85 | 280 | 289 | 1 |  | K.IDFDLKEYAK.D |
| 1308.6888 | 1307.6816 | 1307.6874 | -4.43 | 365 | 375 | 1 |  | K.FAELQSPNKFK.V |
| 1367.7317 | 1366.7244 | 1366.7357 | -8.25 | 451 | 461 | 1 |  | K.LNRYNYIQGVK.M |
| 1422.6897 | 1421.6825 | 1421.6827 | -0.15 | 10 | 20 | 0 |  | K.YIDEHQDLYVK.R |
| 1563.7521 | 1562.7448 | 1562.8127 | -43.41 | 400 | 413 | 1 |  | K.AMKTVFGVEPDLTR.E |
| 1578.7950 | 1577.7878 | 1577.7838 | 2.52 | 10 | 21 | 1 |  | K.YIDEHQDLYVKR.L |
| **1578.8302** | **1577.8229** | **1577.7838** | **24.8** | **10** | **21** | **1** | **112** | **K.YIDEHQDLYVKR.L** |
| 1629.8265 | 1628.8192 | 1628.8120 | 4.46 | 462 | 475 | 1 |  | K.MLGAYLYEVSQLKD.- |
| 1734.8899 | 1733.8827 | 1733.8836 | -0.53 | 414 | 430 | 0 |  | R.EGGSIPVTLTFQEATGK.N |
| 1891.9488 | 1890.9415 | 1890.9476 | -3.19 | 311 | 329 | 0 |  | R.YPSLSLHGIEGAFSASGAK.T |
| 1915.1062 | 1914.0989 | 1914.1190 | -10.46 | 67 | 84 | 1 |  | K.QKLPDGSEIPLPPIILGK.L |
| 1970.0387 | 1969.0315 | 1969.0421 | -5.41 | 22 | 38 | 1 |  | R.LAEWVAIQSVSAWPEKR.A |
| **1970.0923** | **1969.0850** | **1969.0421** | **21.8** | **22** | **38** | **1** | **92** | **R.LAEWVAIQSVSAWPEKR.A** |
| 2066.0038 | 2064.9965 | 2064.9898 | 3.22 | 431 | 450 | 0 |  | K.NVMLLPVGAADDGAHSQNEK.L |
| 2234.1239 | 2233.1166 | 2233.128 | -5.07 | 309 | 329 | 1 |  | R.WRYPSLSLHGIEGAFSASGAK.T |
| 2276.1553 | 2275.1481 | 2275.1484 | -0.16 | 129 | 149 | 1 |  | R.GSTDDKGPVLAWLNALEAYQK.T |
| 2449.2160 | 2448.2087 | 2448.2179 | -3.77 | 431 | 453 | 1 |  | K.NVMLLPVGAADDGAHSQNEKLNR.Y |
| 2626.2413 | 2625.2340 | 2625.2336 | 0.16 | 376 | 398 | 0 |  | K.VYLGHGGKPWVSDFDHPHYMAGR.K |

**MS/MS Fragmentation of K.YIDEHQDLYVKR.L**

**MS/MS Fragmentation of R.LAEWVAIQSVSAWPEKR.A**

**Spot No. 17 gi|50982399 annexin A6 [Gallus gallus]**

| **Observed Mr** | **Mr(expt)** | **Mr(calc)** | **ppm** | **Start Seq.** | **End Seq.** | **Miss** | **Ion Score**  2413.2 | **Peptide sequence** |
| --- | --- | --- | --- | --- | --- | --- | --- | --- |
| 772.4067 | 771.3994 | 771.4028 | -4.31 | 554 | 559 | 0 |  | R.SYPHLR.R |
| 890.5046 | 889.4974 | 889.5022 | -5.38 | 587 | 594 | 0 |  | R.DAFVAIVR.S |
| 896.4976 | 895.4903 | 895.4804 | 11.1 | 561 | 567 | 0 |  | R.VFQEFVK.M |
| 899.5266 | 898.5194 | 898.5236 | -4.72 | 219 | 226 | 0 |  | K.ISGKPIER.S |
| 928.5156 | 927.5084 | 927.5039 | 4.86 | 554 | 560 | 1 |  | R.SYPHLRR.V |
| 1037.5480 | 1036.5407 | 1036.5342 | 6.33 | 598 | 606 | 0 |  | K.NKPAFFADK.L |
| 1044.5200 | 1043.5127 | 1043.4998 | 12.4 | 211 | 218 | 0 |  | R.MVFDEYLK.I |
| 1052.5872 | 1051.5799 | 1051.5815 | -1.48 | 560 | 567 | 1 |  | R.RVFQEFVK.M |
| 1055.6520 | 1054.6448 | 1054.6499 | -4.83 | 498 | 507 | 0 |  | R.ILVSLALGNR.D |
| 1072.5994 | 1071.5921 | 1071.5924 | -0.28 | 629 | 637 | 0 |  | R.SEIDLLNIR.G |
| 1073.5064 | 1072.4991 | 1072.4825 | 15.5 | 249 | 257 | 0 |  | R.STAEYFAER.L |
| 1091.5362 | 1090.5290 | 1090.5328 | -3.55 | 280 | 288 | 0 |  | R.SEIDMLDIR.E |
| 1099.6626 | 1098.6554 | 1098.6583 | -2.7 | 155 | 164 | 0 |  | K.MLVVLLQGAR.E |
| 1142.5860 | 1141.5787 | 1141.5768 | 1.75 | 74 | 82 | 1 |  | K.YELTGKFER.L |
| 1322.6232 | 1321.6159 | 1321.6197 | -2.9 | 568 | 578 | 0 |  | K.MTNHDVEHAIR.K |
| 1535.7873 | 1534.7801 | 1534.7926 | -8.17 | 581 | 594 | 1 |  | R.MSGDVRDAFVAIVR.S |
| 1622.8182 | 1621.8109 | 1621.8134 | -1.51 | 280 | 292 | 1 |  | R.SEIDMLDIREVFR.T |
| 1660.9106 | 1659.9033 | 1659.9018 | 0.92 | 83 | 97 | 0 |  | R.LIVSLMRPPAYSDAK.E |
| 1668.8399 | 1667.8326 | 1667.8308 | 1.1 | 190 | 203 | 0 |  | K.WGTDEAQFIYILGR.R |
| 1671.8946 | 1670.8873 | 1670.8839 | 2.03 | 376 | 391 | 0 |  | K.GLGTDEGAIIEVLTQR.S |
| 1679.7683 | 1678.7610 | 1678.7646 | -2.11 | 530 | 545 | 0 |  | K.LADVASNDSSDSLETR.F |
| **1707.9170** | **1706.9097** | **1706.8839** | **15.1** | **33** | **48** | **1** | **145** | **K.GFGSDKDAILDLITSR.S** |
| 1793.8351 | 1792.8279 | 1792.8228 | 2.84 | 482 | 497 | 1 |  | K.SLEDDLSSDTSGHFKR.I |
| 1963.9655 | 1962.9582 | 1962.9568 | 0.73 | 445 | 463 | 0 |  | K.AVEGAGTDESTLIEIMATR.N |
| 2003.9527 | 2002.9454 | 2002.9345 | 5.45 | 353 | 371 | 0 |  | R.GTVQPASNFNDDGDAQVLR.K |
| 2092.0586 | 2091.0513 | 2091.0518 | -0.2 | 444 | 463 | 1 |  | R.KAVEGAGTDESTLIEIMATR.N |
| 2105.0190 | 2104.0117 | 2103.9973 | 6.86 | 464 | 481 | 0 |  | R.NNQEIAAINEAYQQAYHK.S |
| 2132.0512 | 2131.0439 | 2131.0294 | 6.81 | 353 | 372 | 1 |  | R.GTVQPASNFNDDGDAQVLRK.A |
| 2136.0567 | 2135.0495 | 2135.0283 | 9.91 | 121 | 138 | 1 |  | R.TNQEIHDLVAAYKDAYER.D |
| 2500.3763 | 2499.3690 | 2499.3155 | 21.4 | 112 | 133 | 1 |  | K.CLIEILASRTNQEIHDLVAAYK.D |
| 2690.3834 | 2689.3761 | 2689.3671 | 3.37 | 498 | 522 | 1 |  | R.ILVSLALGNRDEGPENLTQAHEDAK.V |
| 2900.4609 | 2899.4536 | 2899.4485 | 1.77 | 155 | 180 | 1 |  | K.MLVVLLQGAREEDDVVSEDLVEQDAK.D |

**MS/MS Fragmentation of K.GFGSDKDAILDLITSR.S**

**Spot No.18 gi|110591367 Chain A, The Structure Of Chicken Mitochondrial Pepck**

| Observed Mr | Mr(expt) | Mr(calc) | ppm | Start Seq. | End Seq. | Miss | Ion Score  2413.2 | Peptide sequence |
| --- | --- | --- | --- | --- | --- | --- | --- | --- |
| 839.5623 | 838.5550 | 838.5025 | 62.6 | 163 | 170 | 0 |  | R.VGPAVLQR.L |
| 879.4837 | 878.4764 | 878.4134 | 71.8 | 171 | 177 | 0 |  | R.LDDDFVR.C |
| 961.6246 | 960.6173 | 960.5545 | 65.4 | 522 | 529 | 0 |  | R.VLAWIFGR.I |
| 964.5387 | 963.5314 | 963.4662 | 67.8 | 14 | 21 | 0 |  | R.DFVEEAVR.L |
| 1007.5631 | 1006.5558 | 1006.4832 | 72.2 | 303 | 310 | 1 |  | K.FDDEGRLR.A |
| 1092.5791 | 1091.5718 | 1091.5005 | 65.4 | 438 | 447 | 0 |  | R.HGVFMGSAMR.S |
| 1120.7213 | 1119.7140 | 1119.6400 | 66.1 | 203 | 212 | 0 |  | R.VLVAHIPSER.R |
| **1134.6956** | **1133.6884** | **1133.6193** | **60.9** | **340** | **349** | **0** | **50** | **R.NTIFTNVGLR.S** |
| 1134.7037 | 1133.6964 | 1133.6193 | 68 | 340 | 349 | 0 |  | R.NTIFTNVGLR.S |
| 1159.6675 | 1158.6602 | 1158.5815 | 67.9 | 329 | 339 | 0 |  | R.TNPNAMATIAR.N |
| 1182.6696 | 1181.6623 | 1181.5829 | 67.2 | 317 | 328 | 0 |  | R.GFFGVAPGTSSR.T |
| 1231.7517 | 1230.7444 | 1230.6662 | 63.6 | 497 | 505 | 0 |  | R.LFHVNWFLR.D |
| 1257.7940 | 1256.7868 | 1256.7088 | 62 | 1 | 13 | 0 |  | -.LSTSLSALPAAAR.D |
| 1276.8341 | 1275.8268 | 1275.7411 | 67.2 | 203 | 213 | 1 |  | R.VLVAHIPSERR.I |
| 1287.7270 | 1286.7198 | 1286.6309 | 69.1 | 511 | 521 | 0 |  | R.FVWPGFGHNAR.V |
| **1329.8091** | **1328.8019** | **1328.7241** | **58.5** | **426** | **437** | **0** | **84** | **R.GVPLVVEAFGWR.H** |
| 1329.8183 | 1328.8110 | 1328.7241 | 65.4 | 426 | 437 | 0 |  | R.GVPLVVEAFGWR.H |
| **1374.7981** | **1373.7908** | **1373.7092** | **59.4** | **479** | **489** | **0** | **61** | **R.YLEHWLSTGLR.S** |
| 1374.8079 | 1373.8007 | 1373.7092 | 66.6 | 479 | 489 | 0 |  | R.YLEHWLSTGLR.S |
| 1388.8525 | 1387.8452 | 1387.7460 | 71.5 | 44 | 56 | 0 |  | R.GLQDDGVLHPLPK.Y |
| 1390.7668 | 1389.7596 | 1389.6446 | 82.8 | 593 | 604 | 0 |  | R.DVMAELEGLEER.V |
| 1437.8802 | 1436.8729 | 1436.7776 | 66.3 | 534 | 546 | 0 |  | R.DTARPTPIGWVPK.E |
| 1509.8003 | 1508.7930 | 1508.6830 | 72.9 | 57 | 68 | 1 |  | K.YDNCWLARTDPR.D |
| 1530.7864 | 1529.7791 | 1529.6787 | 65.7 | 580 | 592 | 0 |  | R.EYYGENFGADLPR.D |
| 1555.9209 | 1554.9137 | 1554.8042 | 70.4 | 214 | 229 | 0 |  | R.IVSFGSGYGGNSLLGK.K |
| 1574.8857 | 1573.8784 | 1573.7745 | 66 | 277 | 290 | 0 |  | K.TNLAMMTPSLPGWR.I |
| 1645.9043 | 1644.8970 | 1644.8141 | 50.4 | 593 | 606 | 1 |  | R.DVMAELEGLEERVR.K |
| 1700.0177 | 1699.0104 | 1698.9053 | 61.9 | 163 | 177 | 1 |  | R.VGPAVLQRLDDDFVR.C |
| 1764.0434 | 1763.0361 | 1762.9288 | 60.9 | 143 | 158 | 0 |  | K.LGVQVTDSPYVVLSMR.I |
| 1857.0385 | 1856.0312 | 1855.9105 | 65 | 406 | 422 | 0 |  | R.WDDPEGVPIDAIIFGGR.R |
| 1928.0604 | 1927.0531 | 1926.9224 | 67.8 | 577 | 592 | 1 |  | R.QLREYYGENFGADLPR.D |
| 2127.1723 | 2126.1650 | 2125.9978 | 78.6 | 461 | 478 | 0 |  | R.LMHDPFAMRPFFGYNAGR.Y |
| 2203.3124 | 2202.3051 | 2202.1644 | 63.9 | 1 | 21 | 1 |  | -.LSTSLSALPAAARDFVEEAVR.L |

**MS/MS Fragmentation of R.NTIFTNVGLR.S**

**MS/MS Fragmentation of R.GVPLVVEAFGWR.H**

**MS/MS Fragmentation of R.YLEHWLSTGLR.S**

**Spot No. 19 gi|45382893 calbindin 1, 28kDa [Gallus gallus]**

| **Observed Mr** | **Mr(expt)** | **Mr(calc)** | **ppm** | **Start Seq.** | **End Seq.** | **Miss** | **Ion Score**  2413.2 | **Peptide sequence** |
| --- | --- | --- | --- | --- | --- | --- | --- | --- |
| 927.4630 | 926.4557 | 926.4498 | 6.39 | 61 | 68 | 0 |  | K.AFVDQYGK.A |
| 944.5168 | 943.5095 | 943.5338 | -25.76 | 163 | 170 | 0 |  | K.LELTELAR.L |
| 1091.6433 | 1090.6360 | 1090.6386 | -2.37 | 126 | 134 | 1 |  | K.SFLKDLLQK.A |
| 1268.6490 | 1267.6417 | 1267.6482 | -5.1 | 144 | 153 | 0 |  | K.LTEYTEIMLR.M |
| 1284.6572 | 1283.6499 | 1283.6431 | 5.31 | 144 | 153 | 0 |  | K.LTEYTEIMLR.M + Oxidation (M) |
| 1286.5448 | 1285.5375 | 1285.5397 | -1.75 | 100 | 109 | 0 |  | K.SSEDFMQTWR.K |
| 1302.5484 | 1301.5412 | 1301.5347 | 5 | 100 | 109 | 0 |  | K.SSEDFMQTWR.K + Oxidation (M) |
| 1313.7896 | 1312.7823 | 1312.7755 | 5.24 | 171 | 181 | 0 |  | R.LLPVQENFLIK.F |
| 1410.7383 | 1409.7310 | 1409.7337 | -1.86 | 238 | 250 | 1 |  | K.SIMALSDGGKLYR.A |
| 1414.6415 | 1413.6342 | 1413.6347 | -0.35 | 100 | 110 | 1 |  | K.SSEDFMQTWRK.Y |
| 1421.7737 | 1420.7665 | 1420.7561 | 7.26 | 226 | 237 | 1 |  | K.ELDINNLATYKK.S |
| 1426.7424 | 1425.7351 | 1425.7286 | 4.57 | 238 | 250 | 1 |  | K.SIMALSDGGKLYR.A + Oxidation (M) |
| 1430.6340 | 1429.6267 | 1429.6296 | -2.01 | 100 | 110 | 1 |  | K.SSEDFMQTWRK.Y + Oxidation (M) |
| 1616.8389 | 1615.8317 | 1615.8318 | -0.08 | 36 | 48 | 0 |  | K.ELQNFIQELQQAR.K |
| **1616.8858** | **1615.8785** | **1615.8318** | **28.9** | **36** | **48** | **0** | **101** | **K.ELQNFIQELQQAR.K** |
| 1777.8958 | 1776.8886 | 1776.9080 | -10.92 | 248 | 262 | 1 |  | K.LYRAELALILCAEEN.- |
| 1869.8529 | 1868.8456 | 1868.8428 | 1.49 | 110 | 125 | 1 |  | R.KYDSDHSGFIDSEELK.S |
| 1936.9426 | 1935.9353 | 1935.9360 | -0.36 | 154 | 170 | 1 |  | R.MFDANNDGKLELTELAR.L |
| 1952.9593 | 1951.9520 | 1951.9309 | 10.8 | 154 | 170 | 1 |  | R.MFDANNDGKLELTELAR.L + Oxidation (M) |
| 1968.9851 | 1967.9778 | 1967.9874 | -4.84 | 138 | 153 | 1 |  | K.QIEDSKLTEYTEIMLR.M |
| 1985.0078 | 1984.0005 | 1983.9823 | 9.2 | 138 | 153 | 1 |  | K.QIEDSKLTEYTEIMLR.M + Oxidation (M) |
| 2448.3371 | 2447.3298 | 2447.3464 | -6.79 | 74 | 94 | 0 |  | K.IGIVELAQVLPTEENFLLFFR.C |

**MS/MS Fragmentation of K.ELQNFIQELQQAR.K**

**Spot No. 20-1 gi|45382893 calbindin 1, 28kDa [Gallus gallus]**

| **Observed Mr** | **Mr(expt)** | **Mr(calc)** | **ppm** | **Start Seq.** | **End Seq.** | **Miss** | **Ion Score**  2413.2 | **Peptide sequence** |
| --- | --- | --- | --- | --- | --- | --- | --- | --- |
| 927.4612 | 926.4539 | 926.4498 | 4.46 | 61 | 68 | 0 |  | K.AFVDQYGK.A |
| **927.5066** | **926.4993** | **926.4498** | **53.5** | **61** | **68** | **0** | **54** | **K.AFVDQYGK.A** |
| 1091.6199 | 1090.6126 | 1090.6386 | -23.88 | 126 | 134 | 1 |  | K.SFLKDLLQK.A |
| 1268.6333 | 1267.6260 | 1267.6482 | -17.52 | 144 | 153 | 0 |  | K.LTEYTEIMLR.M |
| 1286.5387 | 1285.5314 | 1285.5397 | -6.45 | 100 | 109 | 0 |  | K.SSEDFMQTWR.K |
| 1313.7798 | 1312.7726 | 1312.7755 | -2.21 | 171 | 181 | 0 |  | R.LLPVQENFLIK.F |
| 1421.7523 | 1420.7450 | 1420.7561 | -7.86 | 226 | 237 | 1 |  | K.ELDINNLATYKK.S |
| 1616.8292 | 1615.8219 | 1615.8318 | -6.09 | 36 | 48 | 0 |  | K.ELQNFIQELQQAR.K |
| **1616.8911** | **1615.8838** | **1615.8318** | **32.2** | **36** | **48** | **0** | **106** | **K.ELQNFIQELQQAR.K** |
| 1741.7443 | 1740.7370 | 1740.7479 | -6.25 | 111 | 125 | 0 |  | K.YDSDHSGFIDSEELK.S |
| 1869.8440 | 1868.8367 | 1868.8428 | -3.28 | 110 | 125 | 1 |  | R.KYDSDHSGFIDSEELK.S |
| 1936.9314 | 1935.9241 | 1935.9360 | -6.15 | 154 | 170 | 1 |  | R.MFDANNDGKLELTELAR.L |
| 1968.9641 | 1967.9568 | 1967.9874 | -15.5 | 138 | 153 | 1 |  | K.QIEDSKLTEYTEIMLR.M |
| 2448.3382 | 2447.3309 | 2447.3464 | -6.35 | 74 | 94 | 0 |  | K.IGIVELAQVLPTEENFLLFFR.C |

**MS/MS Fragmentation of K.AFVDQYGK.A**

**MS/MS Fragmentation of K.ELQNFIQELQQAR.K**
