# Supplementary material for: Proteomic analysis of chicken embryonic trachea and kidney tissues after infection in ovo by avian infectious bronchitis coronavirus
Source: Proteome Sci. 2011 Mar 8;9:11. doi: 10.1186/1477-5956-9-11 (PMC3060854; doi:10.1186/1477-5956-9-11)
Supplement: Additional file 4 — Additional_file_4.doc containing the PMF spectrum and Mascot database search results of differentially expressed protein spots in IBV-infected chicken embryo kidney tissues. [file 1477-5956-9-11-S4.DOC]

**Additional file 4**

This includes PMF spectrum and Mascot database search results for 1 differentially expressed protein spots in IBV-infected chicken embryo kidney tissues.

**Spot No.20-2**

**A. PMF spectrum**

**B. PMF database search result**

Match to: **gi|54606655** Score: **385** Expect: **3.3e-32**

**MHC class I antigen [Gallus gallus]**

Number of mass values searched: **52**

Number of mass values matched: **27**

Sequence Coverage: **71%**

Matched peptides shown in **Bold Red**

**1** **MGPCGALGLG LLLAAVCGAA AELHTLR**YIF TAMTDPGPGQ PWFVTVGYVD

**51** GELFVHYNST ARRVVPR**TEW MAANTDQQYL NGQTQIVQGH EQIDRENLGI**

**101 LQRRYNQTGG SHTVQLMYCC DILEDGTIRG YSQDAYDGRD FIALDKGTMT**

**151 FTAPVPEAVP AKR**K**WEEGGV AEGLKQYLEE TCVEWLRRYV EYGKAELGRR**

**201 ERPEVRVWGK EADGILTLPC RAHGFYPRPI AVSWLKDGAV RGQDAQSGGI**

**251 VPNGDGTYHT WVTIDAQPGD GDK**YQCRVEH ASLPQPGLYS WEPPQPNLVP

**301** IVAGVAVAIV AIAIVVGVGF IIYRRHAGKK **GKGYNVAPGS NPAI**

Matched peptides sorted by Residue Number

Start - End Observed Mr(expt) Mr(calc) ppm Miss Sequence

1 - 27 2578.3920 2577.3847 2577.3593 10 0 -.MGPCGALGLGLLLAAVCGAAAELHTLR.Y

68 - 95 3274.5430 3273.5357 3273.5109 8 0 R.TEWMAANTDQQYLNGQTQIVQGHEQIDR.E

96 - 103 942.5620 941.5547 941.5294 27 0 R.ENLGILQR.R

96 - 104 1098.6060 1097.5987 1097.6305 -29 1 R.ENLGILQRR.Y

104 - 129 2973.3330 2972.3257 2972.3579 -11 1 R.RYNQTGGSHTVQLMYCCDILEDGTIR.G

105 - 129 2817.2320 2816.2247 2816.2568 -11 0 R.YNQTGGSHTVQLMYCCDILEDGTIR.G

130 - 139 1131.4380 1130.4307 1130.4629 -28 0 R.GYSQDAYDGR.D

130 - 146 1933.9340 1932.9267 1932.8854 21 1 R.GYSQDAYDGRDFIALDK.G

140 - 146 821.4810 820.4737 820.4331 50 0 R.DFIALDK.G

140 - 162 2419.2990 2418.2917 2418.2505 17 1 R.DFIALDKGTMTFTAPVPEAVPAK.R

147 - 162 1616.8292 1615.8219 1615.8280 -4 0 K.GTMTFTAPVPEAVPAK.R

147 - 163 1772.8980 1771.8907 1771.9291 -22 1 K.GTMTFTAPVPEAVPAKR.K

165 - 175 1174.5360 1173.5287 1173.5666 -32 0 K.WEEGGVAEGLK.Q

165 - 187 2724.2590 2723.2517 2723.2901 -14 1 K.WEEGGVAEGLKQYLEETCVEWLR.R

176 - 187 1568.7690 1567.7617 1567.7341 18 0 K.QYLEETCVEWLR.R

176 - 188 1724.8700 1723.8627 1723.8352 16 1 K.QYLEETCVEWLRR.Y

188 - 194 914.5010 913.4937 913.4657 31 1 R.RYVEYGK.A

189 - 199 1284.6363 1283.6290 1283.6510 -17 1 R.YVEYGKAELGR.R

200 - 206 941.5090 940.5017 940.5202 -20 1 R.RERPEVR.V

201 - 210 1255.6720 1254.6647 1254.6833 -15 1 R.ERPEVRVWGK.E

207 - 221 1657.8540 1656.8467 1656.8658 -11 1 R.VWGKEADGILTLPCR.A

211 - 221 1187.5900 1186.5827 1186.6016 -16 0 K.EADGILTLPCR.A

211 - 236 2910.5890 2909.5817 2909.5374 15 1 K.EADGILTLPCRAHGFYPRPIAVSWLK.D

222 - 241 2240.2530 2239.2457 2239.2014 20 1 R.AHGFYPRPIAVSWLKDGAVR.G

242 - 273 3256.5220 3255.5147 3255.4705 14 0 R.GQDAQSGGIVPNGDGTYHTWVTIDAQPGDGDK.Y

331 - 344 1344.6700 1343.6627 1343.6834 -15 1 K.GKGYNVAPGSNPAI.-

333 - 344 1159.5530 1158.5457 1158.5669 -18 0 K.GYNVAPGSNPAI.-
